# Serological evidence of human infections with highly pathogenic avian influenza A(H5N1) virus: a systematic review and meta-analysis

**DOI:** 10.1186/s12916-020-01836-y

**Published:** 2020-12-02

**Authors:** Xinhua Chen, Wei Wang, Yan Wang, Shengjie Lai, Juan Yang, Benjamin J. Cowling, Peter W. Horby, Timothy M. Uyeki, Hongjie Yu

**Affiliations:** 1 School of Public Health, Fudan University, Key Laboratory of Public Health Safety, Ministry of Education, Shanghai, 200032 China; 2 WorldPop, School of Geography and Environmental Science, University of Southampton, Southampton, SO17 1BJ UK; 3 WHO Collaborating Centre for Infectious Disease Epidemiology and Control, School of Public Health, Li Ka Shing Faculty of Medicine, The University of Hong Kong, Hong Kong, Special Administrative Region China; 4 Centre for Tropical Medicine and Global Health, Nuffield Department of Clinical Medicine, University of Oxford, Oxford, UK; 5 Influenza Division, National Center for Immunization and Respiratory Diseases, Centers for Disease Control and Prevention, Atlanta, USA

**Keywords:** Influenza in humans, Influenza A(H5N1), Serological evidence

## Abstract

**Background:**

Highly pathogenic avian influenza A(H5N1) virus poses a global public health threat given severe and fatal zoonotic infections since 1997 and ongoing A(H5N1) virus circulation among poultry in several countries. A comprehensive assessment of the seroprevalence of A(H5N1) virus antibodies remains a gap and limits understanding of the true risk of A(H5N1) virus infection.

**Methods:**

We conducted a systematic review and meta-analysis of published serosurveys to assess the risk of subclinical and clinically mild A(H5N1) virus infections. We assessed A(H5N1) virus antibody titers and changes in titers among populations with variable exposures to different A(H5N1) viruses.

**Results:**

Across studies using the World Health Organization-recommended seropositive definition, the point estimates of the seroprevalence of A(H5N1) virus-specific antibodies were higher in poultry-exposed populations (range 0–0.6%) and persons exposed to both human A(H5N1) cases and infected birds (range 0.4–1.8%) than in close contacts of A(H5N1) cases or the general population (none to very low frequencies). Seroprevalence was higher in persons exposed to A(H5N1) clade 0 virus (1.9%, range 0.7–3.2%) than in participants exposed to other clades of A(H5N1) virus (range 0–0.5%) (*p* < 0.05). Seroprevalence was higher in poultry-exposed populations (range 0–1.9%) if such studies utilized antigenically similar A(H5N1) virus antigens in assays to A(H5N1) viruses circulating among poultry.

**Conclusions:**

These low seroprevalences suggest that subclinical and clinically mild human A(H5N1) virus infections are uncommon. Standardized serological survey and laboratory methods are needed to fully understand the extent and risk of human A(H5N1) virus infections.

**Supplementary Information:**

The online version contains supplementary material available at 10.1186/s12916-020-01836-y.

## Background

Human infections with highly pathogenic avian influenza (HPAI) A(H5N1) virus were first confirmed in Hong Kong, China, in 1997 [1], in parallel with large outbreaks in domestic poultry [2]. Since the re-emergence of human infections with A(H5N1) virus in Vietnam and China in 2003, the viruses have become entrenched in poultry and continue to evolve in parts of Asia, Africa, and the Middle East [3]. A total of 861 human cases have been reported to the World Health Organization (WHO) from 17 countries since November 2003, including 455 deaths (case fatality proportion of 53% among laboratory-confirmed cases) [4]. On the basis of current knowledge of A(H5N1) epidemics, sporadic poultry-to-human A(H5N1) virus transmission has occurred, accompanied by limited and non-sustained human-to-human transmission [5]. Ongoing A(H5N1) virus circulation among poultry is associated with genetic divergence and emergence of antigenically distinct clades and subclades of A(H5N1) virus in different geographic areas [6, 7]. These evolving genetic and antigenic features of A(H5N1) virus, as well as potential human adaptative mutations [8], continue to pose a pandemic threat.

Despite epidemiological studies of the fatality risk of A(H5N1) virus infections in humans, estimates of the clinical severity remain biased because case-finding has focused upon hospitalized patients with severe pneumonia. Therefore, persons with subclinical or clinically mild A(H5N1) virus infections are rarely ascertained and are not included in the denominator of A(H5N1) virus infections [5]. This under ascertainment bias can lead to substantial underestimates of the infection risk and the case fatality proportion in hospitalized patients overestimates the overall severity of A(H5N1) virus infections [9–11]. Population-based serological studies can be helpful to assess the extent of human A(H5N1) virus infections [12]. Since 1997, many serological studies were conducted in endemic countries. However, these studies enrolled people with different kinds of potential exposures to A(H5N1) viruses, e.g., poultry exposures or case contacts of symptomatic A(H5N1) cases, and have reported a wide range of antibody seroprevalence estimates, such as 0–12.7% among poultry workers and 0–1.9% among the general population [13].

To evaluate the serological evidence for A(H5N1) virus infections in humans, a previous systematic review and meta-analysis reported a pooled seroprevalence of antibodies against A(H5N1) virus of 1–2% through assessment of published studies before 2012 [14]. However, the seroprevalence in this study was criticized for not considering many underlying uncertainties [15]. First, the quality of serological testing methods [e.g., selection of antibody titer to define a seropositive result and antigenic similarity between A(H5N1) virus antigens in assays and circulating A(H5N1) viruses in poultry] was not considered, which could affect the validity of the estimates [16]. Second, the overall seroprevalence of A(H5N1) virus-specific antibodies may be overestimated if the overall denominator among studies did not include all exposed individuals. In addition, these studies neglected the characteristics [e.g., level and different types of exposures to A(H5N1) virus] of participants. Given these limitations, there is a need for improved estimates of the risk of A(H5N1) virus infection among populations with different exposures to A(H5N1) virus.

In this study, we aimed to systematically evaluate the risk of subclinical and clinically mild A(H5N1) virus infections in humans during 1997–2020 by comparing A(H5N1) virus antibody titers, and changes in titers, among different populations, accounting for factors such as the level and type of exposure to A(H5N1) virus, specific virus clade, and methodological quality in reported studies.

## Methods

### Search strategy and selection criteria

In this systematic review and meta-analysis, we searched MEDLINE/PubMed, Embase, CENTRAL, and Web of Science databases for articles published between January 1, 1997, and September 1, 2020, using predefined search terms. Citations from MEDLINE were not presented alone as PubMed comprises all citations that come from MEDLINE-indexed journals. Details of the search strategy are presented in Additional File 1: Table S1. To identify more relevant gray literatures, we also searched the following authoritative databases/websites: Open Grey, Grey Matters, Grey Literature Report, ClinicalTrials.gov, and The British Library. Abstracts of research articles and gray literatures (i.e., dissertation, conference paper/abstract, or technical/other reports) were included if they reported data on the seroprevalence of A(H5N1) virus-specific antibodies, despite full text was not available or peer-reviewed. Studies were excluded if they were reviews, published study protocols without reports on the results of serological tests, or case reports; referred to study subjects previously reported in publications; only assessed the seroprevalence of A(H5N1) virus antibodies in animals; or reported laboratory-confirmed cases regardless of clinical severity. In addition, we checked reference lists of relevant studies to identify potentially eligible studies for this systematic review. This systematic review was conducted according to guidance from the Cochrane handbook of interventions and reported per PRISMA guidelines (http://www.prisma-statement.org/). The review protocol is available in PROSPERO (ID: CRD42020147759). Quality assessment of individual studies was done using the scoring system developed by Sikkema et al. [16]. Based on their overall score, each study’s quality was classified into one of four categories: A, B, C, or D. Category A spanned studies with scores ranging from 15 to 18, category B from 10 to 14, category C from 5 to 9, and category D from 0 to 4. Two researchers independently reviewed and identified eligible articles based on title and abstract, and then based upon the review of full-text articles. Where the two reviewers disagreed on inclusion, a third researcher was consulted and consensus was attained before recording an entry in the database. A full study protocol outlining case definitions, data extraction, variable list, and grades for study quality is available in the Supplementary material (Additional File 1: Table S2-S5). Publication bias was investigated by constructing funnel plots and formally tested using Egger’s line regression test when ten or more studies were included in the primary analyses.

### Data analysis

From eligible studies, we extracted data for three predefined A(H5N1) virus antibody outcomes in humans: (i) seroprevalence, (ii) seroconversion, and (iii) seroincidence. Seroprevalence was defined as the prevalence of A(H5N1) virus-specific antibodies at or above a particular titer to define a seropositive result in cross-sectional studies. Seroconversion was defined as at least a fourfold increase in A(H5N1) virus-specific antibody titers in serum collected at multiple time points. Seroincidence was defined as the number of individuals with serologic evidence of A(H5N1) virus infection divided by total person-time during follow-up visits. For multi-year studies, to reduce variations in seroprevalence caused by differences in the follow-up period among studies, only baseline seroprevalence data were included to estimate overall A(H5N1) virus antibody seroprevalence among different populations. Once extracted, the study populations were reclassified into eleven groups according to differences in occupational and behavioral exposures to A(H5N1) virus (e.g., poultry workers, poultry cullers, poultry-exposed residents, household contacts, social contacts, healthcare workers, mixed poultry and human exposures, and the general population) (Additional File 1: Table S6). The onset date for all laboratory-confirmed human A(H5N1) cases and HPAI A(H5N1) outbreaks in domestic/wild birds were extracted from the World Health Organization (WHO) and Global Animal Disease Information System data (http://empres-i.fao.org/eipws3g/).

Although WHO published recommended laboratory procedures (Additional File 1: Text 1) [6, 16–21] for serologic confirmation of A(H5N1) cases with acute febrile illness and respiratory symptoms, this guidance did not apply to serological studies when A(H5N1) virus infection was not suspected. Because of the lack of a standardized definition of a seropositive result for serologic studies of A(H5N1) virus infection among non-ill persons, random effects models were performed using three different antibody titer thresholds (i.e., WHO recommended, modified WHO recommended, and non-standardized) to estimate overall A(H5N1) virus antibody seroprevalence, seroconversion, and seroincidence rates, and corresponding 95% confidence intervals (CIs). WHO-recommended antibody titers to define a seropositive result in ill persons are a neutralizing (NT) antibody titer ≥ 1:80 with a positive result using a 2nd confirmatory assay [i.e., hemagglutination inhibition test (HAI) (HAI antibody titer ≥ 1:160), enzyme-linked immunosorbent assay, or western blot assay]. The modified WHO seropositive definition refers to an NT antibody titer ≥ 1:80 with a positive result using a 2nd confirmatory assay (i.e., HAI antibody titer ≥ 1:40, ELISA, or western blot assay). The non-standardized seropositive definition refers to criteria used to define a seropositive result other than the WHO or modified WHO definitions.

In three studies conducted during or soon after the 1997 A(H5N1) outbreak in Hong Kong, target populations were assessed for serologic evidence of infection with a distinct A(H5N1) virus genotype Gs/Gd that differed from studies conducted after 2003. Higher A(H5N1) virus antibody seroprevalence was found in exposed persons in the 1997 Hong Kong studies (3.3%, 95% CI 0.9–5.6%) compared with those conducted during 2001–2017 (0.1%, 95% CI 0.02–0.2%) (*Z* = 3.38, *p* < 0.001) using random effects models. Therefore, we analyzed the findings from these 1997 Hong Kong studies separately from those conducted after 1997. Additionally, we analyzed the impact of virus antigen used in laboratory assays on serological results in a sensitivity analysis according to whether included studies reported the antigenic similarity between circulating virus and virus antigen used in laboratory assay.

To assess the true risk of asymptomatic and symptomatic A(H5N1) virus infections among different populations, studies were screened according to whether they reported any acute respiratory illness (i.e., fever or respiratory symptoms) among participants identified with A(H5N1) virus antibodies shortly before the time of serum collection. The proportion of asymptomatic (${p}_{\mathrm{asym},i}=\frac{\mathrm{Number}\ \mathrm{of}\ \mathrm{asymptomatic}\ \mathrm{infections}}{\mathrm{Total}\ \mathrm{number}\ \mathrm{of}\ \mathrm{infections}}$) and symptomatic (${p}_{\operatorname{sym},i}=\frac{\mathrm{Number}\ \mathrm{of}\ \mathrm{symptomatic}\ \mathrm{infections}}{\mathrm{Total}\ \mathrm{number}\ \mathrm{of}\ \mathrm{infections}}$) infections in study population *i* were calculated in studies that ascertained acute respiratory illness in participants. In sensitivity analyses, these proportions were applied to estimate the number of asymptomatic (*n*_1_) and symptomatic (*n*_2_) A(H5N1) virus infections in studies that did not ascertain acute respiratory illness in participants (Additional File 1: Tables S12-S13). The *n*_1, *i*_ and *n*_2, *i*_ are defined as *n*_1, *i*_ = *p*_sym, *i*_ ∙ *P*_*i*_ and *n*_2, *i*_ = *p*_sym, *i*_ ∙ *P*_*i*_, where *P*_*i*_ is the total number of A(H5N1) virus infections detected in serologic study population *i* at a particular antibody titer threshold. Random effects models were then performed to estimate the mean prevalence of asymptomatic and symptomatic A(H5N1) virus infections and corresponding 95% CIs using the estimated number of asymptomatic and symptomatic A(H5N1) virus infections.

The extent to which study-level variables were associated with A(H5N1) virus antibody seroprevalence was examined by the fitting of multivariable meta-regression models using restricted maximum likelihood. To determine the extent of variation between the studies, heterogeneity tests (chi-squared test) with Higgins’ *I*^2^ statistic were used to measure the proportion of the variation. A higher *I*^2^ statistic and a low *p* value (or a large *χ*^2^ statistic relative to its degree of freedom) provide evidence of heterogeneity of A(H5N1) virus antibody seroprevalence among different studies. Subgroup analyses were performed when appropriate to assess A(H5N1) virus antibody seroprevalence by epidemic period, geographical region, virus clade, and potential risk factors. All statistical analyses were performed using statistical software R (version 3.6.0).

## Results

**Fig. 1:**
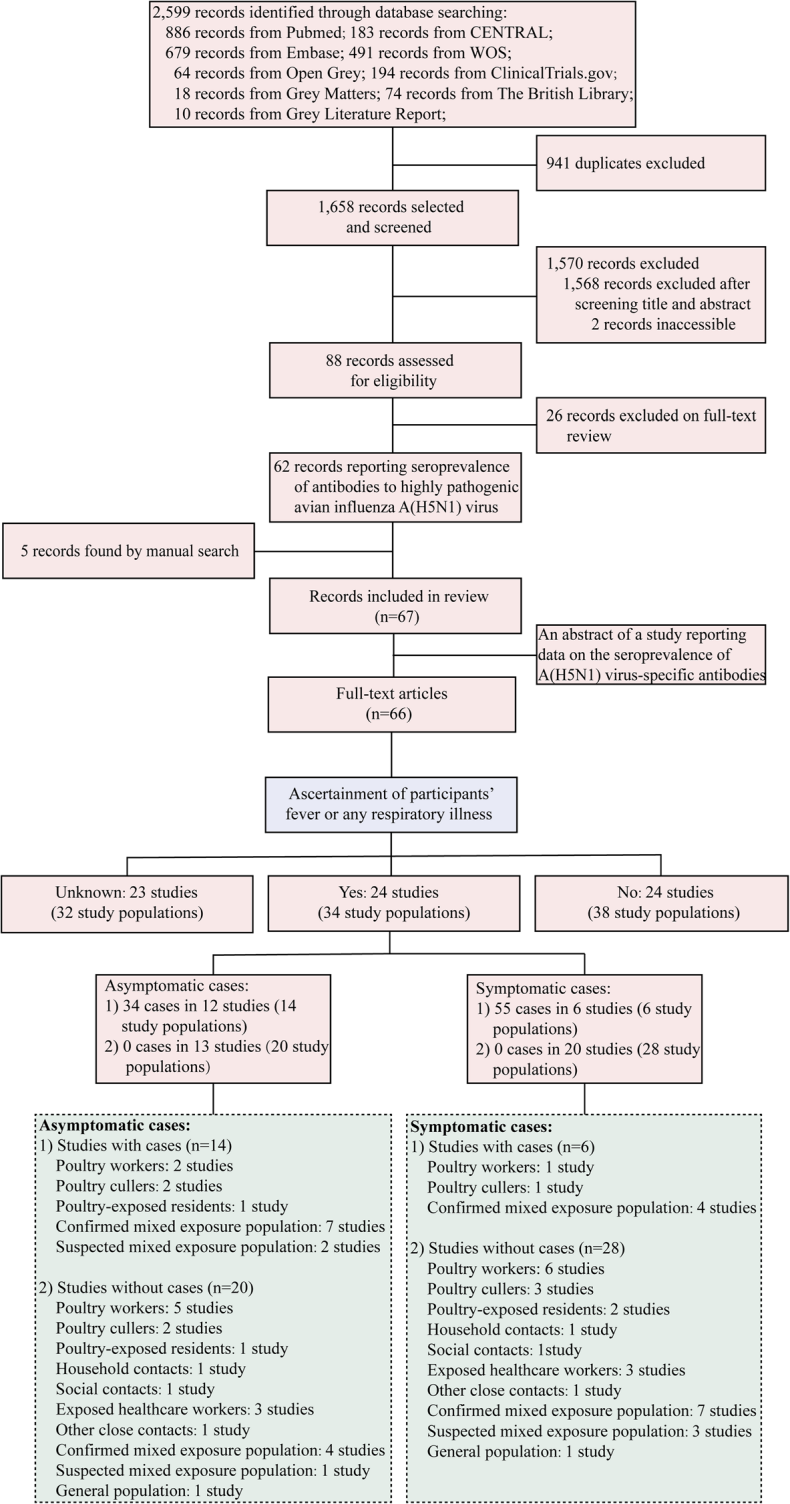
Flowchart of the selection of A(H5N1) serological studies, 1997–2020

A total of 2599 articles/reports/theses were identified, of which 941 were duplicates (Fig. 1). The remaining 1658 articles were screened, of which 61 met the inclusion criteria in the main analysis. An additional five eligible studies identified by screening of reference lists in previous reports [13, 14, 22, 23] were included in this systematic review and meta-analysis. Of the 66 included studies [24–89], twenty-four studies involving 34 study populations ascertained influenza-like illness among participants with A(H5N1) virus antibodies (Fig. 1). When reviewing the study quality (Additional File 1: Table S7-S8), most studies (48/66, 72.7%) were graded as C (Additional File 1: Fig. S1 and Table S9).

**Fig. 2:**
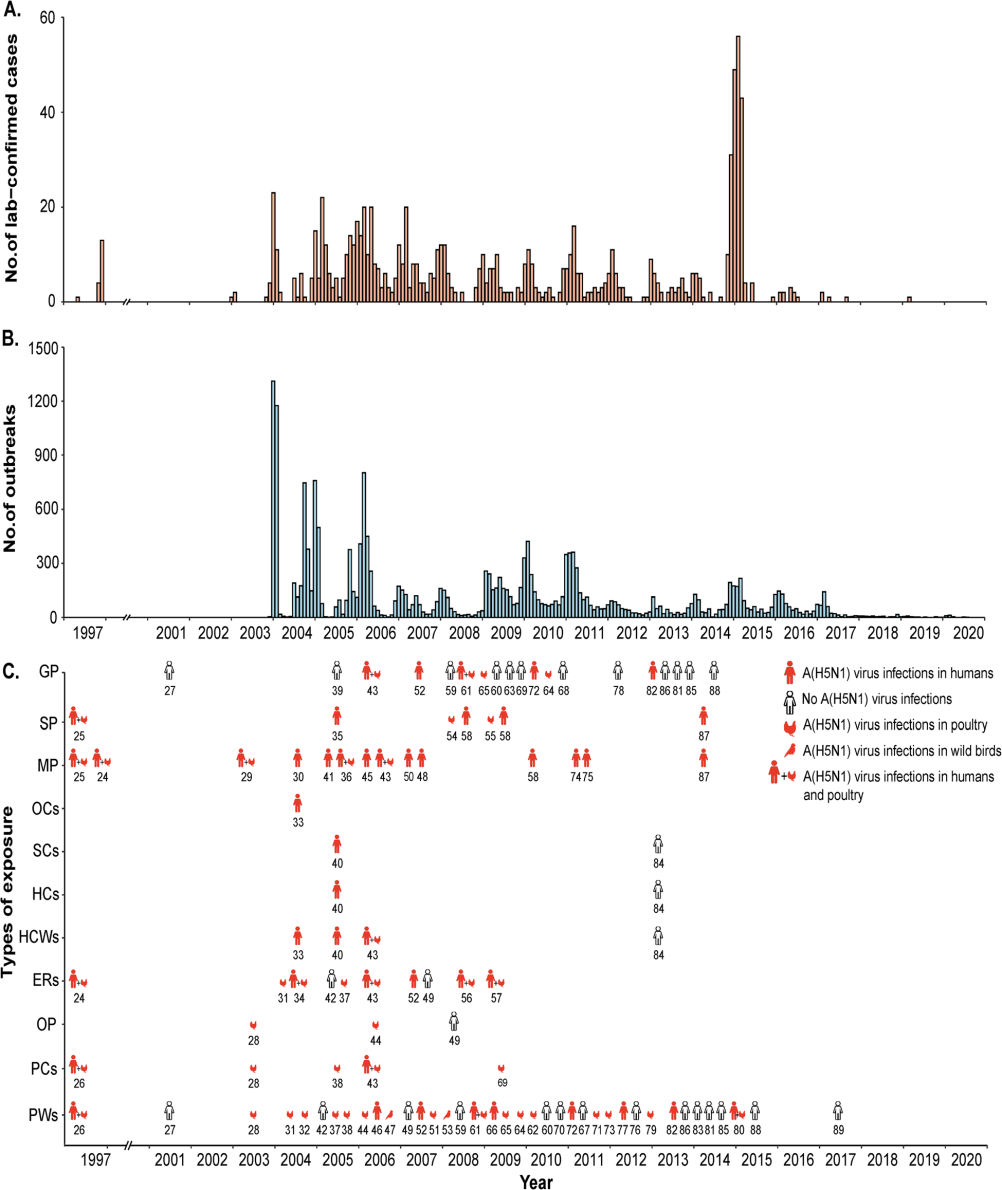
Epidemic curves of highly pathogenic avian influenza A(H5N1) virus infections in humans and animal reservoirs by country and temporal distribution of sixty-six A(H5N1) serosurveys in human by type of exposure, 1997–2020. **a** Epidemic curve of lab-confirmed human infections with highly pathogenic avian influenza A(H5N1) viruses. **b** Epidemic curve of lab-confirmed A(H5N1) outbreaks in poultry and wild birds. **c** Temporal distribution of the implementation of sixty-six A(H5N1) serological studies in poultry workers (PW), poultry cullers (PC), other occupationally exposed populations (OP), poultry-exposed residents (ER), exposed healthcare workers (HCW), household contacts (HC), social contacts (SC), other close contacts (OC), persons with both poultry and human exposures (MP), suspected mixed exposure population (SP), and general population (GP). In panel **c**, the color represents whether A(H5N1) virus infection occurred in humans, poultry, or wild birds occurred (red) or not (white) before or during the implementation of each study. The number below each symbol is the study reference number. The symbols in red color refer to A(H5N1) outbreaks that occurred in one of three species (i.e., humans, domestic poultry, or wild birds), while the human+chicken symbols together refer to A(H5N1) outbreaks that occurred in both humans and domestic poultry. Note that three multi-year cohort studies were classified as independent studies in panel **c**

**Fig. 3:**
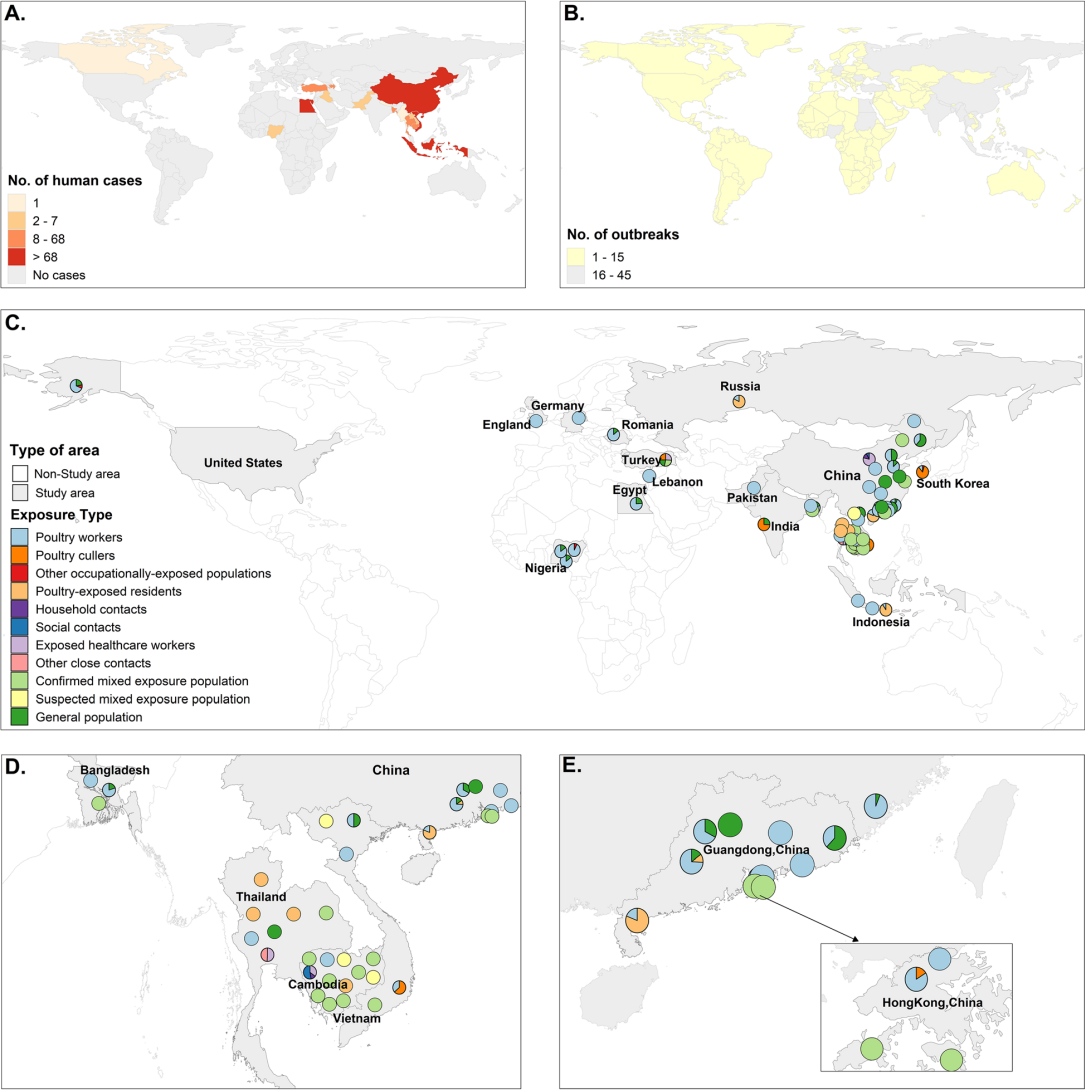
Geographical distribution of highly pathogenic avian influenza A(H5N1) virus infections in human and animal reservoirs by country and distribution of sixty-six A(H5N1) virus antibody serosurveys in humans by type of exposure, 1997–2020. **a** The geographical distribution of laboratory-confirmed human cases of highly pathogenic avian influenza A(H5N1) virus infection. **b** The geographical distribution of A(H5N1) outbreaks in domestic poultry and wild birds. **c**–**e** The geographical distribution of 66 A(H5N1) serosurveys in humans by type of exposure. Note that one human A(H5N1) case reported in Canada was in a returned traveler from China

Figures 2 and 3 show the temporal and spatial distribution of lab-confirmed A(H5N1) virus outbreaks in humans and animal reservoirs and of sixty-six A(H5N1) virus antibody serosurveys. A(H5N1) virus antibody serosurveys were conducted in humans in association with A(H5N1) human or animal outbreaks, except for four studies that aimed to investigate A(H5N1) virus antibody seroprevalence in the general population. The number of A(H5N1) virus antibody serosurveys conducted in humans increased during 2003–2017 when the number of laboratory-confirmed human cases and the number of affected countries increased in parts of Asia, Africa, and Middle East (Spearman’s correlation *r*_s_ = 0.428, *p* = 0.037). Among the 66 studies, a majority (39/66, 59.1%) focused on occupationally exposed populations, and 17 studies were conducted among close contacts of confirmed human A(H5N1) cases.

**Fig. 4:**
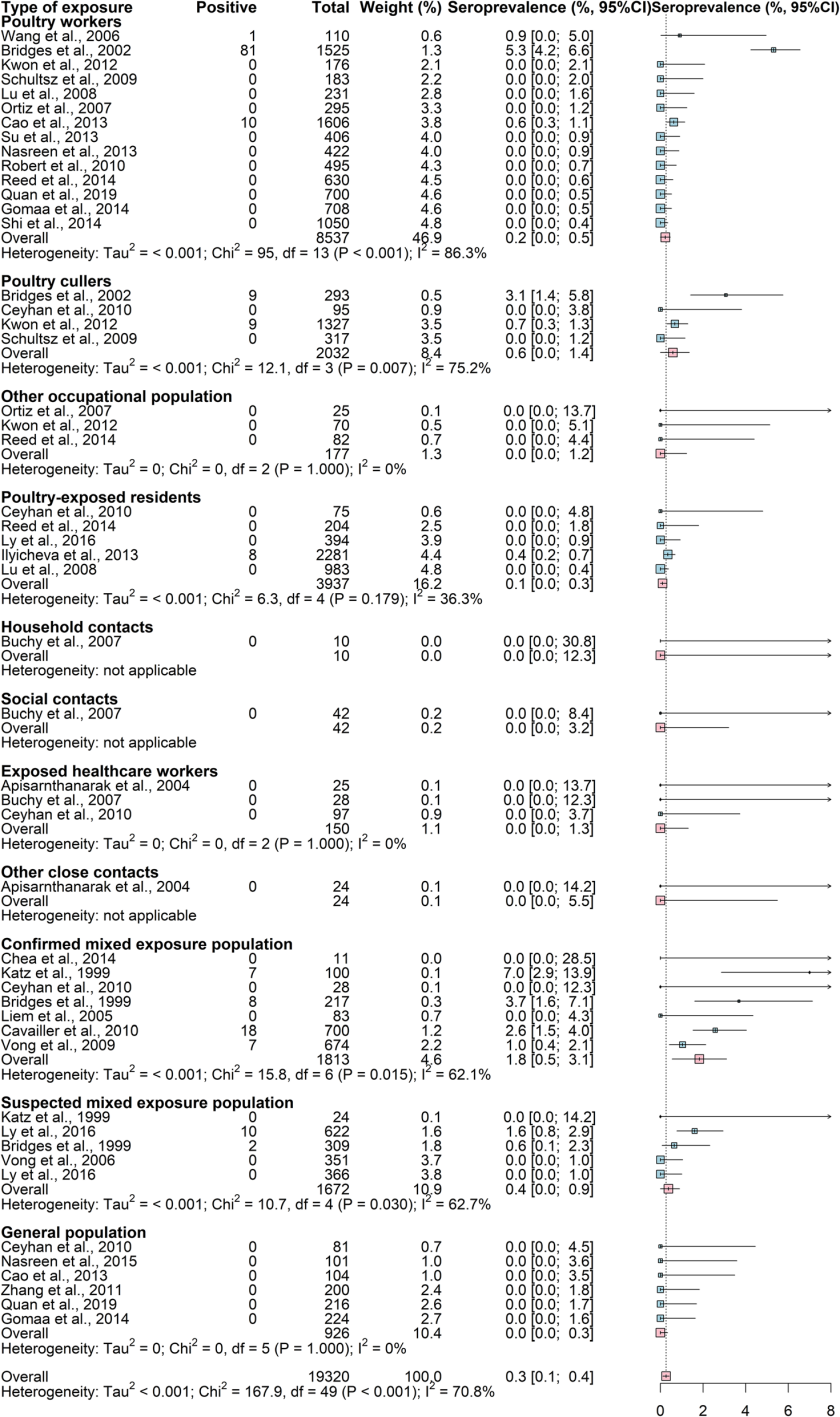
Seroprevalence of antibodies to highly pathogenic avian influenza A(H5N1) virus estimated through random effects models, using standard antibody titers for seropositivity recommended by the World Health Organization. The World Health Organization (WHO) recommendations refer to a neutralizing (NT) antibody titer ≥ 1:80 with a positive result using a 2nd assay method, i.e., hemagglutination inhibition (HAI) assay (HAI antibody titer ≥ 1:160), enzyme-linked immunosorbent assay, or western blot assay

We excluded three studies [55, 57, 60] that presented data previously reported in three other included studies [54, 56, 59]. Of the remaining 63 studies, 27 were conducted among a total of 19,320 participants during 1997 to 2016 in five countries and utilized the WHO-recommended seropositive antibody titer threshold (Fig. 4). These studies reported A(H5N1) virus antibody seroprevalence ranging from 0 to 7.0% (median 0%). Of the remaining studies, two studies in four countries utilized the modified WHO criteria to define a seropositive result. A(H5N1) virus antibody seroprevalence was reported as 0% in more than half of the studies that used the WHO or the modified WHO seropositive definition (Additional File 1: Fig. S2). A(H5N1) virus antibody seroprevalence ranged from 0 to 12.7% in 35 studies that applied a non-standardized seropositive definition, resulting in a pooled seroprevalence of 0.2% (95% CI 0.1–0.3%) among 22,920 participants (Additional File 1: Fig. S3).

**Fig. 5:**
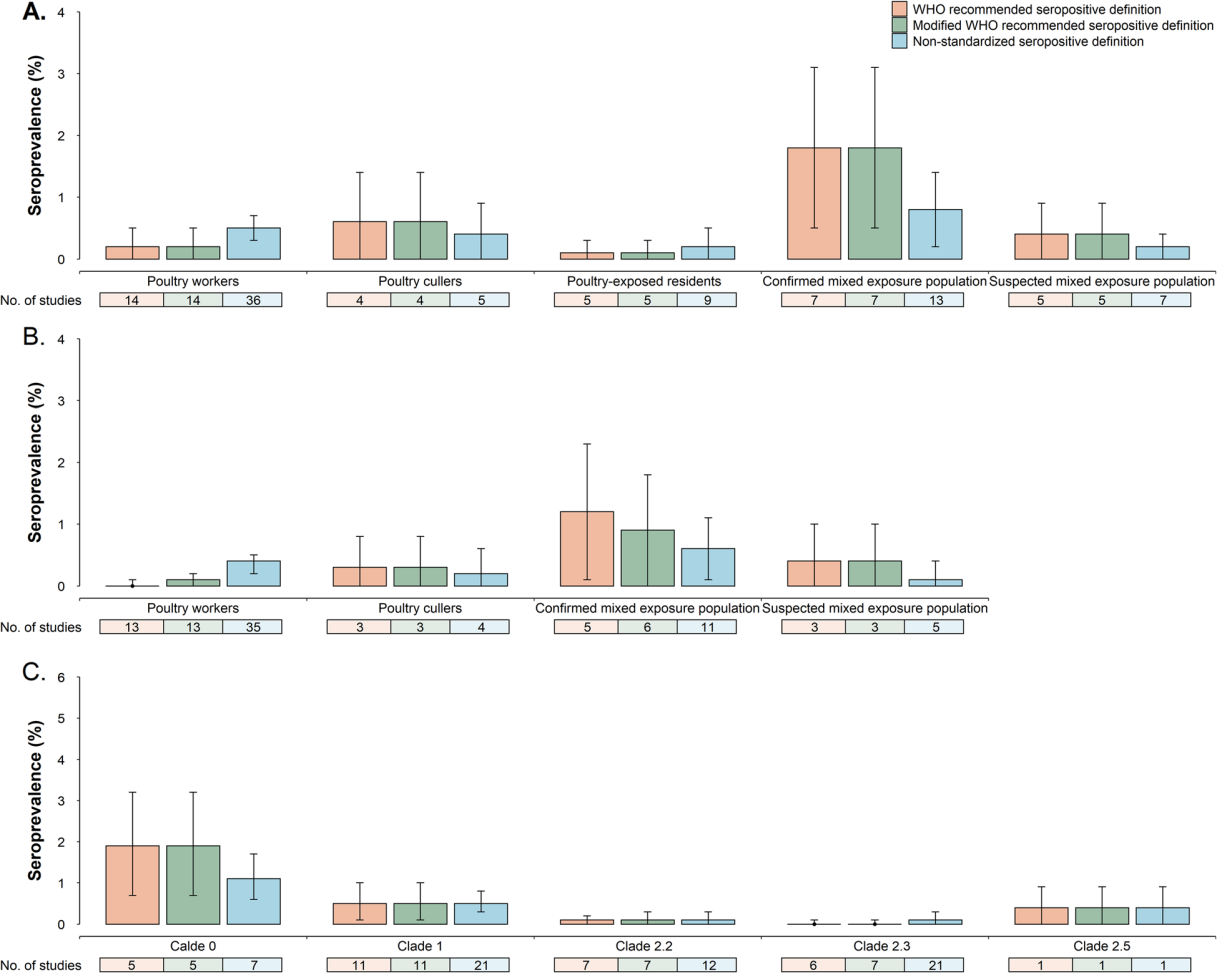
Seroprevalence of antibodies to avian influenza A (H5N1) virus estimated through random effects models by type of exposure and virus clade, using three antibody titer thresholds (World Health Organization recommended, modified World Health Organization recommended, and non-standardized antibody titer thresholds to define a seropositive result). World Health Organization (WHO) recommendations refer to a neutralizing (NT) antibody titer ≥ 1:80 with a positive result using a 2nd assay method, i.e., hemagglutination inhibition test (HAI) (HAI antibody titer ≥ 1:160), enzyme-linked immunosorbent assay, or western blot assay. The modified WHO definition of a seropositive result refers to an NT antibody titer ≥ 1:80 with a positive result using a 2nd confirmatory assay (i.e., HAI antibody titer ≥ 1:40, ELISA, or western blot assay). The non-standardized seropositive definition refers to criteria used to define a seropositive result other than the WHO or modified WHO definitions. Data are presented if A(H5N1) virus-specific antibodies were detected. **a** A(H5N1) virus antibody seroprevalence by type of exposure. **b** Changes in A(H5N1) virus antibody seroprevalence by type of exposure after excluding studies related to A(H5N1) outbreaks in Hong Kong in 1997. **c** A(H5N1) virus antibody seroprevalence by virus clade

Across studies that utilized the WHO seropositivity criteria, the mean seroprevalence was 0.2, 0.6, and 1.8% for poultry workers, poultry cullers, and persons with both poultry and human exposures, respectively, while the mean seroprevalence was 0% among the general population and close contacts of confirmed A(H5N1) cases using random effects models (Fig. 5a, Additional File 1: Table S10). Similarly, poultry workers (0.5%, 95% CI 0.3–0.7%), poultry cullers (0.4%,95% CI 0.0–0.9%), and persons with poultry and human exposures (0.8%, 95% CI 0.2–1.4%) had relatively higher A(H5N1) virus antibody seroprevalence than those without poultry exposures, using the non-standardized seropositive definition. There was high heterogeneity of A(H5N1) virus antibody seroprevalence in poultry workers (*I*^2^ = 86.3%, *p* < 0.001), poultry-exposed residents (*I*^2^ = 85.1%, *p* < 0.001), poultry cullers (*I*^2^ = 73.8%, *p* = 0.004), and persons with both poultry and human exposures (*I*^2^ = 71.7%, *p* < 0.001), while the other study populations did not demonstrate heterogeneity for A(H5N1) virus antibody seroprevalence. After excluding studies conducted during the 1997 Hong Kong outbreak, using the WHO-recommended definition, mean seroprevalence was higher in confirmed mixed exposure population (1.2%) than in poultry workers (0%, *p* = 0.015) or poultry-exposed residents (0.1%, *p* = 0.022). Similar non-significant trends were also present when considering modified definitions (*p* > 0.05) (Fig. 5b). However, with the exception of poultry workers, there were no significant differences in A(H5N1) virus antibody seroprevalence among different exposed populations when the three seropositive criteria were applied (*p* > 0.05). The seroprevalence of A(H5N1) virus-specific antibody was higher among occupationally exposed populations in studies that have antigenically similar A(H5N1) virus antigens in assays to circulating A(H5N1) viruses (range 0.3–0.8), and in studies that used hemagglutination inhibition assay with horse erythrocytes (range 0.5–0.7) when the WHO or modified WHO antibody titer threshold to define seropositive results was applied (Additional File 1: Table S11).

Overall, persons with poultry exposures experienced relatively higher seroprevalence of A(H5N1) virus-specific antibodies than non-poultry-exposed participants in sixty serological studies (Additional File 1: Fig. S4). Additional File 1: Figs. S5-S6 and Tables S12-S13 also indicate a higher seroprevalence of asymptomatic or symptomatic A(H5N1) virus infections among poultry-exposed participants than those without poultry exposures. Poultry-exposed participants who were in areas with A(H5N1) outbreaks among poultry had significantly higher levels of A(H5N1) virus-specific antibodies (0.6%, 95% CI 0.3–0.9%) than those in non-epidemic areas (0.1%, 95% CI 0–0.2%) (Z = 2.92, *p* = 0.004). With the exception of participants exposed to A(H5N1) clade 1 and clade 2.5 viruses in studies that utilized the non-standardized seropositive definition, study participants exposed to A(H5N1) clade 0 virus had relatively higher seroprevalence (range 1.1–1.9%) of A(H5N1) virus-specific antibodies than participants exposed to other A(H5N1) virus clades (range 0–0.5%) (*p* < 0.05) (Fig. 5c). Meta-regression and subgroup analyses (Additional File 1: Fig. S7) revealed that populations with poultry exposures only experienced an increased risk of A(H5N1) virus infection (*β* = 0.6, 95% CI 0–1.2, *p* = 0.037) (Table 1), compared to those without any exposure to A(H5N1) virus.

**Table 1:** Multivariable meta-regression for change in seroprevalence of antibodies to highly pathogenic avian influenza A(H5N1) virus by different factors

Study characteristics	All studies (***β*** coefficient^**¶**^, 95% CI)	All studies excluding reports related to A(H5N1) outbreaks in Hong Kong in 1997 (***β*** coefficient, 95% CI)
Year of study		
1997–2002	1.0	–
2003–2017	− 1.7 (− 3.2, − 0.2)*	–
Epidemic region		
Southeast Asia^a^	1.0	1.0
Hong Kong, China	1.8 (− 0.0, 3.7)	–
Mainland China	− 0.4 (− 1.2, 0.4)	− 0.8 (− 1.5, − 0.1)*
Middle East and Africa^b^	− 0.4 (− 1.4, 0.6)	− 0.6 (− 1.6, 0.4)
Other countries^c^	− 0.7 (− 1.7, 0.3)	− 1.0 (− 2.0, − 0.1)*
A(H5N1) outbreaks in poultry		
No	1.0	1.0
Yes	0.2 (− 0.5, 0.8)	− 0.1 (− 0.7, 0.5)
Virus clade		
Clade 0	1.0	1.0
Clade 1	0.2 (− 0.8, 1.1)	− 0.0 (− 0.9, 0.9)
Clade 2	0.2 (− 0.6, 1.1)	0.0 (− 0.8, 0.8)
Study quality		
Category B	1.0	1.0
Category C	− 0.2 (− 0.9, 0.6)	− 0.1 (− 0.8, 0.6)
Category D	0.1 (− 1.1, 1.4)	0.2 (− 1.0, 1.4)
Level of exposure		
Without any exposure	1.0	1.0
Human case contact only	− 0.1 (− 1.5, 1.3)	− 0.3 (− 1.7, 1.1)
Both poultry exposure and human case contact	0.5 (− 0.4, 1.5)	0.2 (− 0.8, 1.2)
Poultry exposure only	0.6 (0.0, 1.2)*	0.5 (− 0.0, 1.1)

*p < 0.05

^¶^The regression coefficient *β* refers to the change in the seroprevalence of A(H5N1) virus-specific antibodies. A negative sign for the coefficient *β* corresponds to a reduction in the seroprevalence of A(H5N1) virus-specific antibodies for given changes in the covariate, while a positive sign corresponds to an increase in the seroprevalence of A(H5N1) virus-specific antibodies

^a^Including Vietnam, Indonesia, Cambodia, Thailand, and Bangladesh

^b^Including Egypt, Turkey, Pakistan, and Nigeria

^c^Including Romania, Russia, South Korea, the USA, England, and Germany

**Fig. 6:**
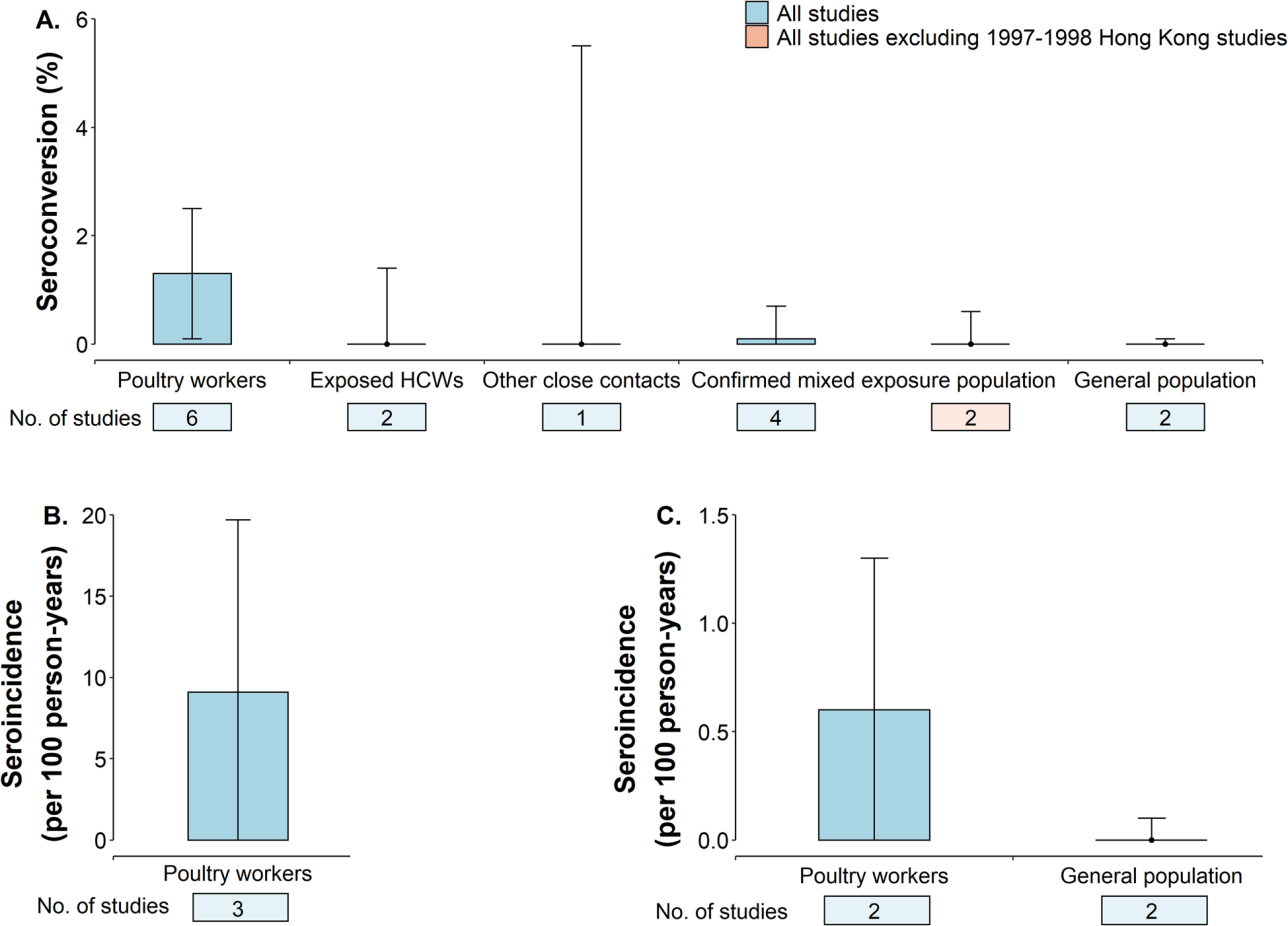
Comparison of seroconversion rate and seroincidence for human infection with highly pathogenic avian influenza A(H5N1) virus estimated through random effects models by type of exposure, using a non-standardized antibody titer threshold. The non-standardized antibody titer threshold refers to criteria to define seropositive results in each study rather than the World Health Organization-recommended or modified World Health Organization-recommended criteria [i.e., a neutralizing (NT) antibody titer ≥ 1:80 with a positive result confirmed by a 2nd assay (i.e., HAI antibody titer ≥ 1:40, ELISA or western blot assay)]. Data are presented for seroconversion rate for human infection with A(H5N1) virus (**a**), and seroincidence of human infection with A(H5N1) virus considering whether A(H5N1) outbreaks in humans or animal reservoirs occurred (**b**) or not (**c**). The red color in panel **a** represents the estimates of the pooled seroconversion rate are based on all thirteen studies excluding reports related to A(H5N1) outbreaks in Hong Kong in 1997

Seroconversion data were available in twelve studies. The median A(H5N1) virus antibody seroconversion rate in these studies was 0% (range 0–44.0%) (Additional File 1: Fig. S8 and Table S14). Poultry workers had the highest A(H5N1) virus antibody seroconversion rate of 1.3% (Fig. 6a). Of the twelve studies, follow-up duration was available in five, allowing estimation of seroincidence. The median follow-up duration was 12 months (range 2.0–40.2 months). Seroincidence rate was much higher in three studies conducted during A(H5N1) outbreaks (9.1 per 100 person-years) (Fig. 6b, Additional File 1: Fig. S9) than in two studies conducted when A(H5N1) outbreaks were not occurring (0.6 per 100 person-years) (Fig. 6c, Additional File 1: Fig. S10). The general population consistently had the lowest mean seroconversion (0.0% 95% CI 0.0–0.1) and seroincidence (0.0, 95% CI 0.0–0.1) rates, regardless of the presence of symptoms (Fig. 6a and c and Additional File 1: Fig. S11).

Observed and tested funnel plot asymmetry revealed evidence of publication bias in studies of the seroprevalence of A(H5N1) virus-specific antibody using three different criteria to define a seropositive result (*p* < 0.001) (Additional File 1: Fig. S12). However, between-study heterogeneity and small-study effects (i.e., the tendency for smaller studies to show greater effects than larger studies) are also possible explanations. After including an abstract of one study that reported data on the seroprevalence of A(H5N1) virus-specific antibody, the estimation of the seroprevalence did not change among poultry workers (Additional File 1: Fig. S13) in a sensitive analysis.

## Discussion

Our findings provide the most comprehensive assessment of the seroprevalence of A(H5N1) virus infections to date and reveal differences in seroprevalence of A(H5N1) virus antibody titers among populations with different occupational and behavioral exposures to A(H5N1) virus. We have shown that higher estimates of A(H5N1) virus antibody seroprevalence have been observed in occupationally and behaviorally exposed populations than those with very low levels of exposures to A(H5N1) virus. In comparison, none or very low frequencies of antibodies against A(H5N1) virus have been detected among all close contacts of confirmed A(H5N1) cases. Our results also show variations in A(H5N1) virus antibody titers among populations exposed to different clades of A(H5N1) virus, with clade 0, related to the Hong Kong A(H5N1) outbreak in 1997, being associated with higher rates of seropositivity than other clades. Importantly, however, there was a lack of standardization of study design and laboratory assay methods among the 66 published studies. This led to generally low overall study quality and inconsistent conclusions about asymptomatic human A(H5N1) virus infections among studies.

Variations in seroprevalence of A(H5N1) virus-specific antibodies seem to be consistent with the extent of reported exposures to A(H5N1) virus and are highest among occupationally and behaviorally exposed populations. Among occupationally exposed populations, persons who worked in live poultry markets had higher frequencies of A(H5N1) virus-specific antibodies than poultry farmers and veterinarians, probably because they were typically involved in more than one high-risk operation (e.g., butchering and processing poultry). Despite prolonged and concentrated poultry exposures among occupational populations, these populations likely had a very low risk of A(H5N1) virus infection when A(H5N1) outbreaks among poultry had not occurred in the study region. Although very few or no seropositive results have been detected among close contacts of A(H5N1) cases, we cannot exclude the potential higher risk among blood-related individuals. As demonstrated in previous studies, the preponderance of familial clustering of cases (50/54, 92.6%) [90] and an increased relative risk of secondary infections in blood relatives of A(H5N1) index cases (RR = 8.96, 95% CI 1.30–61.86) [91] suggests that there may be familial susceptibility risk factors for A(H5N1) virus infection.

Heterogeneity in the seroprevalence of A(H5N1) virus-specific antibodies among included serological studies may in part be due to a lack of standardization of survey and laboratory methods for testing A(H5N1) virus-specific antibodies. Generally, the design and conduct of such studies can affect the quality and interpretability of the serological evidence, and the measurement of A(H5N1) virus-specific antibody titers has been problematic due to the presence of cross-subtype antibodies and natural antibody kinetics. For example, Shimizu et al. [79] reported a very high seroconversion rate of 44% based on a relatively low HAI antibody titer threshold that was likely a consequence of detection of false-positive, cross-subtype antibodies and therefore may have substantially overestimated the true number of A(H5N1) virus infections that had occurred [20, 92]. Also, an overly long interval between exposures and serum sampling will possibly lead to uninterpretable results, since antibody titers may have decayed below the antibody titer to define a seropositive result due to the kinetics of antibody titers following mild or asymptomatic A(H5N1) virus infection [93].

To accurately and reliably estimate subclinical A(H5N1) virus infections that have occurred, standardized methods for study procedures and laboratory assays should be used for sero-epidemiological investigations. Although the Consortium for the Standardization of Influenza Seroepidemiology (CONSISE) developed protocols to standardize sero-epidemiological investigation [12, 94], no antibody titer to confirm asymptomatic or clinically mild A(H5N1) virus infections was recommended. Echoing the recommendations of WHO, performing a 2nd confirmatory assay after a screening serological assay can be helpful in reliably detecting subclinical A(H5N1) virus infections. A(H5N1) virus-specific T cell responses, which have low or no cross-reactivity with H3 or H1 HA peptides and are most likely to have been generated as a result of prior infection with or exposure to a low level of A(H5N1) virus, should also be assessed [95].

Our study has multiple limitations. First, our classification of populations with different types of exposures to A(H5N1) virus was based upon limited information available from published studies, and thus, some participants’ exposures might be misclassified. Second, because of the inaccessibility of data on the frequency of exposure among different populations, we cannot quantify the risk of A(H5N1) virus infections among populations with different kinds of exposures. Third, the estimates of A(H5N1) virus antibody seroprevalence among these populations cannot be extrapolated to non-epidemic populations, due to variations in levels of exposure to A(H5N1) virus among study populations involved in these studies and the variability in infectivity and transmissibility of A(H5N1) virus strains.

## Conclusion

In conclusion, this study provides the best available estimates to date of the overall seroprevalence of A(H5N1) virus-specific antibodies among populations with different occupational and behavioral exposures to A(H5N1) virus. A lack of standardization of survey and laboratory methods to test for A(H5N1) virus-specific antibodies has resulted in heterogeneities in the reported seroprevalence of A(H5N1) virus-specific antibodies among different sero-epidemiological investigations. In particular, inappropriate antibody titer thresholds to define seropositive results and antigenic dissimilarity between A(H5N1) virus antigens used in assays and A(H5N1) virus strains circulating among poultry can cause challenges in interpreting the risk of human infection with A(H5N1) viruses. Therefore, it is essential that future serological studies adhere to strict antibody titer thresholds (e.g., WHO-recommended criteria) to define seropositive results, and apply standardized survey and laboratory methods as recommended by the CONSISE statement [12].

## Supplementary Information

**Additional file 1: Text 1.** Materials and Methods. **Table S1.** Search strategy used in this systematic review. **Table S2**. Characteristics of eligible studies. **Table S3**. Summary of antibody detection assays in eligible studies. **Table S4**. Data describing seroprevalence of antibodies to A(H5N1) virus, prior seasonal influenza vaccination and infections, risk factors for A(H5N1) virus infections. **Table S5**. Summary of studies reporting seroconversion rate and seroincidence of human A(H5N1) infections. **Table S6**. Definition of subjects. **Table S7**. Scoring system used for evaluation of eligible studies. **Table S8**. Scores for antibody detection assays. **Table S9**. Quality assessment of eligible studies. **Table S10**. Seroprevalence of antibodies to A(H5N1) virus by type of exposure. **Table S11**. Seroprevalence of antibodies to A(H5N1) virus, considering antigenic similarity between virus strains circulating among poultry and antigens used in laboratory assays. **Table S12 and S13**. Seroprevalence of antibodies to A(H5N1) virus by type of exposure and virus clade in studies without ascertainment of influenza-like illness in participants. **Table S14**. Seroconversion rate and seroincidence estimates of human A(H5N1) infections by type of exposure. **Fig. S1**. Quality score by type of exposure. **Fig. S2 and S3**. Seroprevalence of antibodies to A(H5N1) virus by type of exposure, using modified WHO recommended and non-standardized antibody titer threshold. **Fig. S4**. Relative risk of human A(H5N1) infections by type of exposure. **Figs. S5 and S6**. Estimated seroprevalence of antibodies to A(H5N1) virus in asymptomatic or symptomatic persons by type of exposure or virus clade. **Fig. S7**. Subgroup analysis of seroprevalence of antibodies to A(H5N1) virus. **Fig. S8**. Estimated seroconversion rates of human A(H5N1) infections by type of exposure. **Fig S9 and S10**. Estimated seroincidence of human A(H5N1) infections among studies with and without A(H5N1) outbreaks. **Fig S11.** Estimated seroconversion rate and seroincidence of asymptomatic human A(H5N1) infections by type of exposure. **Fig S12.** Funnel plot with pseudo 95% confidence limits. **Fig S13.** Estimated seroprevalence of antibodies to A(H5N1) virus in all studies, regardless of the availability of full-text.

## Data Availability

The datasets used and analyzed during the current study are available in Additional file 1.
