# Supplementary material for: Serological evidence of human infections with highly pathogenic avian influenza A(H5N1) virus: a systematic review and meta-analysis
Source: BMC Med. 2020 Dec 2;18:377. doi: 10.1186/s12916-020-01836-y (PMC7709391; doi:10.1186/s12916-020-01836-y)
Supplement: Supplementary file 1 — Additional file 1: Text 1. Materials and Methods. Table S1. Search strategy used in this systematic review. Table S2. Characteristics of eligible studies. Table S3. Summary of antibody detection assays in eligible studies. Table S4. Data describing seroprevalence of antibodies to A(H5N1) virus, prior seasonal influenza vaccination and infections, risk factors for A(H5N1) virus infections. Table S5. Summary of studies reporting seroconversion rate and seroincidence of human A(H5N1) infections. Table S6. Definition of subjects. Table S7. Scoring system used for evaluation of eligible studies. Table S8. Scores for antibody detection assays. Table S9. Quality assessment of eligible studies. Table S10. Seroprevalence of antibodies to A(H5N1) virus by type of exposure. Table S11. Seroprevalence of antibodies to A(H5N1) virus, considering antigenic similarity between virus strains circulating among poultry and antigens used in laboratory assays. Table S12 and S13. Seroprevalence of antibodies to A(H5N1) virus by type of exposure and virus clade in studies without ascertainment of influenza-like illness in participants. Table S14. Seroconversion rate and seroincidence estimates of human A(H5N1) infections by type of exposure. Fig. S1. Quality score by type of exposure. Fig. S2 and S3. Seroprevalence of antibodies to A(H5N1) virus by type of exposure, using modified WHO recommended and non-standardized antibody titer threshold. Fig. S4. Relative risk of human A(H5N1) infections by type of exposure. Figs. S5 and S6. Estimated seroprevalence of antibodies to A(H5N1) virus in asymptomatic or symptomatic persons by type of exposure or virus clade. Fig. S7. Subgroup analysis of seroprevalence of antibodies to A(H5N1) virus. Fig. S8. Estimated seroconversion rates of human A(H5N1) infections by type of exposure. Fig S9 and S10. Estimated seroincidence of human A(H5N1) infections among studies with and without A(H5N1) outbreaks. Fig S11. Estimated seroconversion rate and seroincide [file 12916_2020_1836_MOESM1_ESM.docx]

**Supplementary material**

**Contents**

[Text 1-Materials and Methods 4](#_Toc53166863)

[Appendix Tables 9](#_Toc53166864)

[Table S1. Search strategy for PubMed, CENTRAL, Embase, Web of science and other databases/websites. 9](#_Toc53166865)

[Table S2. Descriptive characteristics of serological studies included in the systematic review 14](#_Toc53166866)

[Table S3. Summary of antibody detection assays to identify human infection with highly pathogenic avian influenza A(H5N1) virus among sixty-five serological studies included in systematic review 37](#_Toc53166867)

[Table S4. Data describing seroprevalence of antibodies to highly pathogenic avian influenza A(H5N1) virus, prior seasonal influenza vaccination and infections, risk factors for A(H5N1) virus infections 53](#_Toc53166868)

[Table S5. Summary of studies reporting seroconversion rate and seroincidence of human infections with highly pathogenic avian influenza A(H5N1) virus included in systematic review 66](#_Toc53166869)

[Table S6. Definition of subjects included in meta-analysis 69](#_Toc53166870)

[Table S7. Scoring system used for evaluation of published reports describing seroevidence of human infection with highly pathogenic avian influenza A(H5N1) virus 72](#_Toc53166871)

[Table S8. Scores for antibody detection assays assigned to published studies on human infection with highly pathogenic avian influenza A(H5N1) virus 73](#_Toc53166872)

[Table S9. Quality assessment of 66 serological studies describing subclinical and clinically mild human infection with highly pathogenic avian influenza A(H5N1) virus, 1997-2019 74](#_Toc53166873)

[Table S10. Apparent and estimated seroprevalence of antibodies to highly pathogenic avian influenza A(H5N1) virus by type of exposure, using three antibody titer thresholds (World Health Organization recommended, modified World Health Organization recommended, and non-standardized) 85](#_Toc53166874)

[Table S11. Sensitivity analysis of seroprevalence of antibodies to highly pathogenic avian influenza A(H5N1) virus, considering antigenic similarity between virus strains circulating among poultry and antigens used in laboratory assays 89](#_Toc53166875)

[Table S12. Sensitivity analysis of seroprevalence of antibodies to highly pathogenic avian influenza A(H5N1) virus by type of exposure in studies without ascertainment of influenza-like illness in participants 91](#_Toc53166876)

[Table S13. Sensitivity analysis of seroprevalence of antibodies to highly pathogenic avian influenza A(H5N1) virus by virus clade in studies without ascertainment of influenza-like illness in participants 93](#_Toc53166877)

[Table S14. Seroconversion rate and seroincidence estimates of human infection with highly pathogenic avian influenza A(H5N1) virus by type of exposure, using non-standardized antibody titer threshold 94](#_Toc53166878)

[Appendix Figures 96](#_Toc53166879)

[Figure S1. Quality score assigned to sixty-five serological studies by type of exposure to A(H5N1) virus, 1997–2020 96](#_Toc53166880)

[Figure S2. Pooled estimates of seroprevalence of antibodies to highly pathogenic avian influenza A(H5N1) virus by type of exposure, using modified WHO recommended antibody titer threshold 97](#_Toc53166881)

[Figure S3. Pooled estimates of seroprevalence of antibodies to highly pathogenic avian influenza A(H5N1) virus by type of exposure, using non-standardized antibody titer criteria 99](#_Toc53166882)

[Figure S4. Relative risk of human infection with highly pathogenic avian influenza A(H5N1) virus by type of exposure, using three antibody titer thresholds to define a seropositive result (World Health Organization recommended, modified World Health Organization recommended and non-standardized) 101](#_Toc53166883)

[Figure S5. Estimated seroprevalence of antibodies to highly pathogenic avian influenza A(H5N1) virus in asymptomatic persons by type of exposure and virus clade, using three antibody titer criteria to define a seropositive result (World Health Organization recommended, modified World Health Organization recommended and non-standardized) 102](#_Toc53166884)

[Figure S6. Estimated seroprevalence of antibodies to highly pathogenic avian influenza A(H5N1) virus in symptomatic persons by type of exposure and virus clade, using three antibody titer criteria to define a seropositive result (World Health Organization recommended, modified World Health Organization recommended and non-standardized) 103](#_Toc53166885)

[Figure S7. Subgroup analysis of seroprevalence of antibodies to highly pathogenic avian influenza A(H5N1) virus by type of exposure, using three antibody titer thresholds to define a seropositive result (World Health Organization recommended, modified World Health Organization recommended and non-standardized) 104](#_Toc53166886)

[Figure S8. Pooled analysis of seroconversion rates of human infection with highly pathogenic avian influenza A(H5N1) virus by type of exposure, using non-standardized antibody titer criteria. 106](#_Toc53166887)

[Figure S9. Pooled analysis of seroincidence of human infection with highly pathogenic avian influenza A(H5N1) virus among studies with A(H5N1) outbreaks, using non-standardized antibody titer criteria 107](#_Toc53166888)

[Figure S10. Pooled analysis of seroincidence of human infection with highly pathogenic avian influenza A(H5N1) virus among studies without A(H5N1) outbreaks, using non-standardized antibody titer criteria 108](#_Toc53166889)

[Figure S11. Estimated seroconversion rate and seroincidence of asymptomatic human infection with highly pathogenic avian influenza A(H5N1) virus by type of exposure, using non-standardized antibody titer criteria. 109](#_Toc53166890)

[Figure S12. Funnel plot (with pseudo 95% confidence limits) in studies of the seroprevalence of A(H5N1) virus-specific antibodies using three antibody titer criteria. 110](#_Toc53166891)

[Figure S13. Estimated seroprevalence of antibodies to highly pathogenic avian influenza A(H5N1) virus in all studies regardless of the availability of methodology, using non-standardized antibody titer criteria. 111](#_Toc53166892)

# Text 1-Materials and Methods

*Case definition*

Standardized criteria for serologic confirmation of human infection with clinically mild or asymptomatic A(H5N1) virus infections do not exist, although the World Health Organization (WHO) proposed recommended laboratory procedures for serologic confirmation of A(H5N1) cases with acute febrile illness and respiratory symptoms. The *WHO criteria* for retrospective serological confirmation of A(H5N1) virus infection in a symptomatic patient specify that paired sera, collected first during the acute phase of illness (within 7 days of symptom onset) and then during the convalescent phase of illness (at least 14 days after collection of the acute sample), should be tested simultaneously. Retrospectively, infection with A(H5N1) virus is confirmed when one of the following criteria are met:

- 4-fold or greater rise in neutralizing antibody titer for A(H5N1) virus in paired sera (acute and convalescent). Convalescent neutralizing antibody titer must be ≥1:80.
- Neutralizing antibody titer for A(H5N1) virus ≥1:80 in a single serum specimen collected at day 14 or later post symptom onset with a positive result using different serological assays (e.g. antibody titer of ≥1:160 in hemagglutination inhibition (HAI) assay using horse red blood cells or an H5-specific western blot assay).

Of note, given the potential cross-reactions between A(H5N1) virus and other antigens, three different antibody titer thresholds were set in this study to conservatively interpret the serological data drawn from previous studies. To detect “true positive” serological results, standard antibody titer threshold for seropositivity recommended by World Health Organization was first applied. WHO recommended antibody titer threshold to define a seropositive result in ill persons are a neutralizing (NT) antibody titer ≥1:80 with a positive result using a 2nd confirmatory assay [i.e. hemagglutination inhibition test (HAI) (HAI antibody titer ≥1:160), enzyme linked immunosorbent assay or western blot assay]. Then, a relatively lower antibody titer threshold (i.e. the modified WHO seropositive definition) was applied to maximize the detection probabilities of human A(H5N1) virus infections. The modified WHO seropositive definition refers to an NT antibody titer ≥1:80 with a positive result using a 2nd confirmatory assay (i.e. HAI antibody titer ≥1:40, ELISA or western blot assay). Also, the non-standardized seropositive definition in each original article was applied to evaluate the impact of such a lower antibody titer threshold on the estimates of seroprevalence of A(H5N1) virus-specific antibodies. The non-standardized antibody titer threshold is defined as the seropositive definition used in each original study rather than a neutralizing (NT) antibody titer ≥1:80 with a positive result confirmed by a 2nd assay (i.e. HAI antibody titer ≥1:40, ELISA or western blot assay). All participants involved in this systematic review were reclassified into three groups (i.e. participants who met WHO recommended, modified WHO, non-standardized seropositive definition) according to the results of each serological study.

*Data extraction and variable list*

We assessed included studies to determine: 1) study population and related types/levels of exposure to A(H5N1) virus; 2) antibody detection assays used; 3) predefined outcomes, i.e. A(H5N1) virus-specific antibody seroprevalence, seroconversion and seroincidence rates; 4) whether the detection of A(H5N1) virus-specific antibodies is attributed to the antibody response produced by prior infection with seasonal influenza A viruses or influenza vaccination.

A complete information list was extracted from qualified studies, including the author’s name, publication date, study design, study period, sampling period, location of participants, study population, exposure setting, frequency of exposure, type of A(H5N1) outbreak in study location, mode of transmission (i.e. poultry-to-human and human-to-human), type of exposures to A(H5N1) virus, days from exposure to serum collection , laboratory methodology for serological detection of A(H5N1) virus antibodies (including specific assay methods, red blood cells used, starting serum dilution, A(H5N1) virus antigen used, A(H5N1) virus clade/genotype involved and positive threshold), laboratory methodology for confirmation of cross-reactivity between A(H5N1) virus and other seasonal influenza A virus antigens (including antibody detection assay methods, red blood cells used, starting serum dilution, antigen used and seropositive antibody titer threshold), the number of participants with seasonal influenza vaccination, the number of participants with seasonal influenza virus infections tested by serological assays, and predefined outcomes (i.e. the total number of participants, the number of participants that provided single or paired sera, the number of seropositive participants, and the number of individuals who seroconverted during the study period).

*Quality assessment of serological studies*

Based on their overall score, the study quality was classified into four categories: A, B, C or D. Category A spanned studies with a scores ranging from 15 to 18, category B from 10 to 14, category C from 5 to 9, and category D from 0 to 4.

*Rationale for the classification of study period included in meta-analyses*

Considering the major replacement of the predominant genotype of A(H5N1) virus since 2003, [Li KS, et al. Nature 2004; Vijaykrishna D, et al. PLoS pathogens 2008; Le TH, et al. Clin Exp Vaccine Res 2014] the study period was classified into two phases: 1) 1997-2002 (dominant genotypes: A-E and their common precursor Gs/Gd), and 2) 2003-2017 (dominant genotype: Z and G).

*Rationale for changing scoring systems for antibody detection assays that focused on human infection with avian influenza A viruses*

Based on the scoring system developed by Sikkema, R. S., et al, we made some modifications to develop a more appropriate scoring system for A(H5N1) virus antibody serological studies that considered the sensitivity of serological assay methods in detecting A(H5N1) virus-specific antibodies. First, available evidence from the Consortium for the standardization of influenza seroepidemiology (CONSISE) and other studies focused on laboratory serological methods reveal that the sensitivity is similar among ELISA-based, HA-based, CPE-based, fluorescence-based microneutralization (MN) and plaque neutralization (PN) assays [Rowe T, et al. J Clin Microbiol 1999; Laurie KL, et al. Clin Vaccine Immunol 2015; Bo H DL, et al. Disease Surveillance 2016]. These assay methods are better than traditional virus neutralization (NT) or hemagglutination inhibition (HAI) assays. Thus, MN, PN and NT assays were assessed the highest score (3 points) while HAI (2 points) was downgraded. Second, pseudovirus particles, most of which were produced by lentivirus expression system, were assigned the same score (3 points) as an MN assay since they were thought to be as effective as live viruses to detect neutralizing antibodies. Third, compared to HAI assays that utilized chicken or turkey RBCs (1 points), HAI assays that used horse RBCs (2 points) were given a higher score since horse RBCs display a high proportion of sialic acid α2,3-Gal binding that is preferential for avian influenza A viruses [Wang X, et al. Clinical infectious diseases, 2014].

# Appendix Tables

Table S1. Search strategy for Medline/PubMed, CENTRAL, Embase, Web of science and other databases/websites.

| **Databases/Websites** | **Step** | **Searching strategy** | **Number of articles*** |
| --- | --- | --- | --- |
| Medline/PubMed | #1 | "Influenza in Birds" [MeSH Terms] | 7,256 |
|  | #2 | H5N1 | 6,612 |
|  | #3 | #1 AND #2 | 2,513 |
|  | #4 | Influenza A Virus | 11,445 |
|  | #5 | avian* OR bird* OR poultry* OR fowl* OR chicken* OR duck* OR geese* OR turkey* OR quail* OR wild bird* OR waterfowl* OR water bird* | 262,781 |
|  | #6 | "Birds" [MeSH Terms] OR "Poultry" [MeSH Terms] OR "Chickens" [MeSH Terms] OR "Ducks" [MeSH Terms] OR "Geese" [MeSH Terms] OR "Turkey" [MeSH Terms] OR "Quail" [MeSH Terms] | 259,480 |
|  | #7 | #5 OR #6 | 360,266 |
|  | #8 | #2 AND #4 AND #7 | 764 |
|  | #9 | "Influenza A Virus, H5N1 Subtype" [MeSH Terms] | 6,056 |
|  | #10 | #3 OR #8 OR #9 | 6,659 |
|  | #11 | human* OR persons OR people OR personnel* OR population OR individual* OR occupational group* OR working population OR working group* OR working-age group* OR working-age population OR worker* OR staff* OR professional* OR farm* OR owner* OR vendor* OR trader* OR slaughterer* OR butcher* | 31,566,919 |
|  | #12 | "Humans" [MeSH Terms] OR "Persons" [MeSH Terms] OR "Population" [MeSH Terms] OR "Occupational Groups" [MeSH Terms] OR "Farmers" [MeSH Terms] OR "Commerce" [MeSH Terms] | 18,925,550 |
|  | #13 | #11 OR #12 | 31,566,919 |
|  | #14 | #10 AND #13 | 6,659 |
|  | #15 | #7 AND #14 | 5,278 |
|  | #16 | swine* OR Suidae OR pig* OR hog* OR pork | 183,236 |
|  | #17 | "Swine" [MeSH Terms] OR "Pork Meat" [MeSH Terms] | 220,736 |
|  | #18 | #16 OR #17 | 316,401 |
|  | #19 | #14 AND #18 | 291 |
|  | #20 | healthcare worker* OR medical staff OR healthcare professional* OR healthcare personnel OR hospital personnel OR nurse* OR nursing OR nursing staff OR physician* OR surgeon* OR veterinarian* | 1,063,517 |
|  | #21 | "Health Personnel" [MeSH Terms] OR "Medical Staff" [MeSH Terms] OR "Personnel, Hospital" [MeSH Terms] OR "Nursing Staff" [MeSH Terms] OR "Nursing Staff, Hospital" [MeSH Terms] OR "Physicians" [MeSH Terms] OR "Surgeons" [MeSH Terms] OR "Veterinarians" [MeSH Terms] | 520,352 |
|  | #22 | #20 OR #21 | 1,373,248 |
|  | #23 | #10 AND #22 | 131 |
|  | #24 | #14 OR #15 OR #19 OR #23 | 6,659 |
|  | #25 | seroprevalence OR seroprevalent OR seronegative OR seropositive OR seropositivity OR seroepidemiology OR seroepidemiologic OR seroepidemiological OR serologic OR serological OR serology OR sero* OR subclinical OR asymptomatic | 608,965 |
|  | #26 | "seroepidemiologic studies" [MeSH Terms] OR "serology" [MeSH Terms] OR "serologic tests" [MeSH Terms] OR "asymptomatic infections" [MeSH Terms] | 202,004 |
|  | #27 | #25 OR #26 | 757,719 |
|  | #29 | #24 **AND** #27 (Best match) | 886 |
| CENTRAL | #1 | "Influenza in Birds" [MeSH Terms] | 92 |
|  | #2 | H5N1 | 348 |
|  | #3 | #1 AND #2 | 59 |
|  | #4 | Influenza A Virus | 3,189 |
|  | #5 | avian* OR bird* OR poultry* OR fowl* OR chicken* OR duck* OR geese* OR turkey* OR quail* OR wild bird* OR waterfowl* OR water bird* | 21,169 |
|  | #6 | "Birds" [MeSH Terms] OR "Poultry" [MeSH Terms] OR "Chickens" [MeSH Terms] OR "Ducks" [MeSH Terms] OR "Geese" [MeSH Terms] OR "Turkey" [MeSH Terms] OR "Quail" [MeSH Terms] | 1,016 |
|  | #7 | #5 OR #6 | 21,177 |
|  | #8 | #2 AND #4 AND #7 | 63 |
|  | #9 | "Influenza A Virus, H5N1 Subtype" [MeSH Terms] | 101 |
|  | #10 | #3 OR #8 OR #9 | 183 |
| Embase | #1 | "avian influenza" [Emtree Terms] | 8,308 |
|  | #2 | H5N1 | 7,251 |
|  | #3 | #1 AND #2 | 2,582 |
|  | #4 | Influenza A Virus | 45,402 |
|  | #5 | avian* OR bird* OR poultry* OR fowl* OR chicken* OR duck* OR geese* OR turkey* OR quail* OR wild bird* OR waterfowl* OR water bird* | 286,616 |
|  | #6 | "bird" [Emtree Terms] OR "poultry" [Emtree Terms] OR "chicken" [Emtree Terms] OR "duck" [Emtree Terms] OR "goose" [Emtree Terms] OR "waterfowl" [Emtree Terms]OR "turkey" [Emtree Terms] OR "quail" [Emtree Terms] | 268,693 |
|  | #7 | #5 OR #6 | 388,455 |
|  | #8 | #2 AND #4 AND #7 | 4,605 |
|  | #9 | "Influenza A virus (H5N1)" [Emtree Terms] | 6,578 |
|  | #10 | #3 OR #8 OR #9 | 8,173 |
|  | #11 | human* OR persons OR people OR personnel* OR population OR individual* OR occupational group* OR working population OR working group* OR working-age group* OR working-age population OR worker* OR staff* OR professional* OR farm* OR owner* OR vendor* OR trader* OR slaughterer* OR butcher* | 7,844,969 |
|  | #12 | "human" [Emtree Terms] OR "population" [Emtree Terms] OR "agricultural worker" [Emtree Terms] | 22,572,747 |
|  | #13 | #11 OR #12 | 8,008,992 |
|  | #14 | #10 AND #13 | 4,151 |
|  | #15 | #7 AND #14 | 4,249 |
|  | #16 | swine* OR Suidae OR pig* OR hog* OR pork | 479,928 |
|  | #17 | "pig" [Emtree Terms] OR "pork" [Emtree Terms] | 230,245 |
|  | #18 | #16 OR #17 | 559,276 |
|  | #19 | #14 AND #18 | 458 |
|  | #20 | healthcare worker* OR medical staff OR healthcare professional* OR healthcare personnel OR hospital personnel OR nurse* OR nursing OR nursing staff OR physician* OR surgeon* OR veterinarian* | 1,410,200 |
|  | #21 | "health care personnel" [Emtree Terms] OR "medical staff" [Emtree Terms] OR "hospital personnel" [Emtree Terms] OR "nursing staff" [Emtree Terms] OR "physician" [Emtree Terms] OR "surgeon" [Emtree Terms] OR "veterinarian" [Emtree Terms] | 1,604,065 |
|  | #22 | #20 OR #21 | 2,410,331 |
|  | #23 | #10 AND #22 | 291 |
|  | #24 | #14 OR #15 OR #19 OR #23 | 5,966 |
|  | #25 | seroprevalence OR seroprevalent OR seronegative OR seropositive OR seropositivity OR seroepidemiology OR seroepidemiologic OR seroepidemiological OR serologic OR serological OR serology OR sero* OR subclinical OR asymptomatic | 800,787 |
|  | #26 | seroprevalence [Emtree Terms] OR seroepidemiology [Emtree Terms] OR serology [Emtree Terms] OR asymptomatic infection [Emtree Terms] | 271,749 |
|  | #27 | #25 OR #26 | 969,623 |
|  | #29 | #24 **AND** #27 (Best match) | 679 |
| Web of Science core collection (topic search) | #1 | Influenza in Birds | 4,792 |
|  | #2 | H5N1 | 9,331 |
|  | #3 | #1 AND #2 | 2,149 |
|  | #4 | Influenza A Virus | 61,542 |
|  | #5 | avian* OR bird* OR poultry* OR fowl* OR chicken* OR duck* OR geese* OR turkey* OR quail* OR wild bird* OR waterfowl* OR water bird* | 536,441 |
|  | #6 | #2 AND #4 AND #5 | 6,108 |
|  | #7 | Influenza A Virus, H5N1 Subtype | 2,247 |
|  | #8 | #3 OR #6 OR #7 | 6,619 |
|  | #9 | human* OR persons OR people OR personnel* OR population OR individual* OR occupational group* OR working population OR working group* OR working-age group* OR working-age population OR worker* OR staff* OR professional* OR farm* OR owner* OR vendor* OR trader* OR slaughterer* OR butcher* | 10,121,200 |
|  | #10 | #8 AND #9 | 4,380 |
|  | #11 | #5 AND #10 | 4,160 |
|  | #12 | swine* OR Suidae OR pig* OR hog* OR pork | 632,217 |
|  | #13 | #10 AND #12 | 532 |
|  | #14 | healthcare worker* OR medical staff OR healthcare professional* OR healthcare personnel OR hospital personnel OR nurse* OR nursing OR nursing staff OR physician* OR surgeon* OR veterinarian* | 907,755 |
|  | #15 | #8 AND #14 | 83 |
|  | #16 | #10 OR #11 OR #13 OR #15 | 4,386 |
|  | #17 | seroprevalence OR seroprevalent OR seronegative OR seropositive OR seropositivity OR seroepidemiology OR seroepidemiologic OR seroepidemiological OR serologic OR serological OR serology OR sero* OR subclinical OR asymptomatic | 600,926 |
|  | #18 | #16 AND #17 | 491 |
| Open Grey | #1 | H5N1 | 64 |
| Grey Literature Report | #1 | H5N1 | 10 |
| Grey Matters | #1 | H5N1 | 18 |
| ClinicalTrials.gov | #1 | H5N1 | 194 |
| The British Library | #1 | H5N1 AND human AND sero* | 74 |

*Searches were conducted on 1 September 2020. Free text was used if MeSH or Emtree terms were not specified. All MeSH and Emtree search terms were exploded. Please note that no citation from Open Grey, Grey Literature Report, Grey Matters, ClinicalTrials.gov, and The British Library were eligible for this systematic review.

Table S2. Descriptive characteristics of serological studies included in the systematic review

| **Reference** | **Location (country)** | **Study period** | **Study type** | **Outbreak identification** | **Source of outbreak** | **Study population** | **No. of participants** | **Age of participants (Median, range/mean±SD)** | **Serology collected (No. of single serum/paired sera/participants)** | **Transmission mode evaluated** | **Comment** |
| --- | --- | --- | --- | --- | --- | --- | --- | --- | --- | --- | --- |
| Katz et al., 1999 | Hong Kong, China | 1997 | Retrospective cohort study | A(H5N1) confirmed patient | Zoonotic source unknown | Household^†^ and non-household contacts, tour group members, exposed and non-exposed co-workers | 51 household contacts, 9 non-household contacts, 26 tour group members, 23 exposed co-workers and 24 non-exposed co-workers | Household contacts: 32 (0.6–58) years-old; non-household contacts: 28 (6–50) years-old; tour group members: 32 (22–55) years-old; exposed co-workers: 36 (range: 22–58) years-old; non-exposed co-workers: 40 (25–58) years-old | Yes (household contacts: unk/unk/51; non-household contacts: unk/unk/9; tour group members: 0/26/26; exposed co-workers: 23/0/23; non-exposed co-workers: 24/0/24) | Possible human-to-human transmission | Paired sera were collected from household and non-household contacts.  Participants with PPE were not reported. |
| Bridges et al., 2000 | Hong Kong, China | 1997 | Retrospective cohort study | A(H5N1) confirmed patient | Zoonotic source unknown | Exposed and non-exposed HCWs | 217 exposed HCWs; 309 non-exposed HCWs | Exposed HCWs (median): 30 years-old; non-exposed HCWs (median): 29 years-old | Yes (exposed HCWs: 23/194/217; non-exposed HCWs: 309/0/309) | Animal-to-human transmission; possible human-to-human transmission | Adults aged ≥60 years and exposed HCWs without a blood sample collected >10 days after the last possible exposure to an index case were excluded from analysis. |
| Bridges et al., 2002 | Hong Kong, China | 1997-98 | Retrospective cohort study; Nested case-control study used to explore the risk factor for H5 antibody | Animal infection | Zoonotic source known | GWs involved in poultry culling operations, PWs exposed to poultry potentially infected with A(H5N1) virus | 293 GWs; 1525 PWs | GWs: 41 (22-58) years-old; PWs: 15-59 years-old | Yes (GWs: 64/229/293; PWs: 1525/0/1525) | Animal-to-human transmission | Adults aged ≥60 or ≤ 14 years were excluded from analysis. |
| Uyeki et al., 2012 | Hanoi, Viet Nam | 2001 | Cross-sectional study | Animal infection | Zoonotic source known | LPM workers and non-exposed population | 200 LPM workers and 200 non-exposed individuals | LPM workers (median):  32 years-old; non-exposed individuals (median): 22 years-old | Yes (LPM workers: 200/0/200; non-exposed individuals: 200/0/200) | Animal-to-human transmission | - |
| Kwon et al., 2012 | South Korea | 2003 | Retrospective cohort study | Animal infection | Zoonotic source known | Poultry farm workers and their household members, cullers (local government workers, soldiers, animal husbandrymen, and civilians), other persons ^**^ | 176 poultry farm workers and their household members, 1327 cullers, 70 other persons | - | Yes (poultry farm workers and their household members: unk/unk/176; cullers: unk/unk/1327, other persons: unk/unk/70) | Animal-to-human transmission | A total of 936 paired sera were collected from 2512 bird cullers. |
| Liem et al., 2005 | Hanoi, Vietnam | 2003-04 | Cross-sectional study | A(H5N1) confirmed patient | Zoonotic source unknown | Exposed HCWs | 83 exposed HCWs | 37.4 (22-55) years-old | Yes (83/0/83) | Possible human-to-human transmission | - |
| Schultsz et al., 2005 | Ho Chi Minh City, Viet Nam | 2004 | Retrospective cohort study | A(H5N1) confirmed patient | Zoonotic source unknown | HCWs | 62 exposed HCWs | 33 (22–54) years-old | Yes (46/14/62) | Possible human-to-human transmission | - |
| Lu et al., 2008 | Guangdong, China | 2004 | Cross-sectional study | Animal infection | Zoonotic source known | Occupational population (raising, selling, and slaughtering chicken and ducks), general citizen population in A(H5N1) outbreak areas | 231 occupational persons, 983 general citizens | - | Yes (occupational persons: 231/0/231; general citizens: 983/0/983) | Animal-to-human transmission | Participants with PPE were not reported. |
| Hinjoy et al., 2008 | Thailand | 2004 | Cross-sectional study | Animal infection | Zoonotic source known | Poultry farmers | 322 | 35 (5-50) years-old | Yes (322/0/322) | Animal-to-human transmission | - |
| Apisarnthanarak et al., 2004 | Thailand | 2004 | Retrospective cohort study | A(H5N1) confirmed patient | Zoonotic source known | Exposed and non-exposed HCWs | 25 exposed HCWs; 24 non-exposed HCWs | Exposed HCWs: 27 (22-33) years-old; non-exposed HCWs: 26 (22-33) years-old | Yes (exposed HCWs: 0/25/25; non-exposed HCWs: 0/24/24) | Animal-to-human transmission; possible human-to-human transmission | PPE was not used for the initial 48 h of exposure; Adults aged ≥60 years and exposed HCWs without a blood sample collected >10 days after the last possible exposure to an index case were excluded from analysis. |
| Khuntirat et al., 2015 | Kamphaeng Phet province, Thailand | 2004-07 | Prospective cohort study | A(H5N1) confirmed patient | Zoonotic source known | Children living in villages with documented A(H5N1) virus infection in poultry or people in 2004-2006 | Baseline: 251; 1-year follow-up: 251; 2-year follow-up: 251 | Range: 4-15 years-old | Yes (baseline: 251/0/251; 1-year follow-up: 0/251/251; 2-year follow-up: 0/251/251) | Animal-to-human transmission | - |
| Vong et al., 2006 | Kampot Province, Cambodia | 2005 | Cross-sectional study | Animal infection and A(H5N1) confirmed patient | Zoonotic source known | Rural residents living within a village where a A(H5N1) confirmed case occurred | 351 rural residents | 23 (1 month-81) years-old | Yes (351/0/351) | Animal-to-human transmission | A substantial proportion of the surveyed population had regular, high-intensity contact with these animals in the 12 months before the survey;  No known evidence of human-to-human transmission |
| Dejpichai et al., 2009 | Prachin Buri, Kamphaeng Phet, Sukhothai, Phetchabun Province, Thailand | 2005 | Cross-sectional study | A(H5N1) confirmed patient | Zoonotic source known | Rural residents living in the same villages as A(H5N1) confirmed or probable case | 901 rural residents in four provinces | 40 (2-101) years-old | Yes (901/0/901) | Animal-to-human transmission; possible human-to-human transmission | Participants were enrolled by convenience sampling, which biased the age distribution of the population in study locations. |
| Santhia et al., 2009 | Bali Province, Indonesia | 2005 | Cross-sectional study | - | - | Market stall operators and residents living in a region where A(H5N1) outbreaks in animals occurred | 841 residents and 87 market stall operators | Residents: mean (52% of respondents): 42 (26-45) years-old | Yes (market stall operators: 87/0/87; residents: 841/0/841) | Animal-to-human transmission | - |
| Schultsz et al., 2009 | Ho Chi Minh City, Viet Nam | 2005 | Cross-sectional study | Animal infection | Zoonotic source known | Poultry workers and cullers | 183 poultry workers and 317 cullers | Poultry workers (age missing for 2 poultry workers): 36 (15–78) years-old; poultry cullers: 42 (22–58) years-old | Yes (poultry workers: 183/0/183; poultry cullers: 317/0/317) | Animal-to-human transmission | - |
| Apisarnthanarak et al., 2006 | Pratumthani, Thailand | 2005-06 | Retrospective cohort study | - | - | ICU patients with community-acquired pneumonia | 115 | - | Yes (0/42/115) | Animal-to-human transmission | Adults ≥60 years of age were excluded from the serologic tests |
| Buchy et al., 2007 | Cambodia | 2005-06 | Retrospective cohort study | A(H5N1) confirmed case | Zoonotic source known | Household member, health care workers, close contact villager | 10 household members, 28 health care workers, 42 close contact villagers | - | Yes (household members: 10/0/10; health care workers: 28/0/28; close contact villagers: 42/0/42) | Possible human-to-human transmission | - |
| Liao et al., 2013 | China | 2005-08 | Retrospective cohort study | A(H5N1) confirmed patient | Zoonotic source known | Household contacts, social contacts | 87 household contacts, 332 social contacts | Household contacts: 32 (IQR: 17–43) years-old, social contacts: 11 (IQR: 9–41) years-old | Yes (household contacts: 35/52/87; social contacts: 136/196/332) | Animal-to-human transmission; possible human-to-human transmission | Adults aged ≥60 years were excluded from analysis. None of the 419 close contacts used appropriate PPE. |
| Ilyicheva et al., 2013 | Western Siberia, Russia | 2005-09 | Surveillance study | Animal infection | Zoonotic source known | Residents and poultry farm personnel living in regions where outbreaks among birds caused by A(H5N1) virus in 2005-2007 | 2230 residents, 51 poultry farm personnel | - | Yes (residents: 2230/0/2230; poultry farm personnel: 51/0/51) | Animal-to-human transmission | - |
| Ceyhan et al., 2010 | Van city, Turkey | 2006 | Cross-sectional study; retrospective cohort study | A(H5N1) confirmed patient; Animal infection | Zoonotic source known | Family contacts, staff members involved in poultry culling operations, exposed and non-exposed population, exposed HCWs | 28 family contacts; 95 culling staff members; 75 exposed individuals; 81 non-exposed individuals; 97 exposed HCWs; | Family contacts: 11 (1-55) years-old; Culling staff: 40 (16-64) years-old; Exposed individuals: 19 (2-70) years-old; Non-exposed individuals: 21 (1-65) years-old; HCWs: 33 (21-46) years-old | Yes (family contacts: 28/0/28; culling staff members: 95/0/95; exposed individuals: 75/0/75; non-exposed individuals: 81/0/81; exposed HCWs: 0/97/97) | Animal-to-human transmission; possible human-to-human transmission | PPE used in caring for patients, and PPE was not used in poultry culling operations |
| Ortiz et al., 2007 | Plateau State, Kano State, Nigeria | 2006 | Cross-sectional study | Animal infection | Zoonotic source known | Poultry workers, and laboratory workers who had been exposed to suspected A(H5N1) virus in culture or in poultry specimens | 295 poultry workers and 25 exposed laboratory workers | Poultry workers: 28 (12-58) years-old; laboratory workers: 42 (range: 28-58) years-old | Yes (poultry workers: 295/0/295; exposed laboratory workers: 25/0/25) | Animal-to-human transmission | Persons >59 years were excluded |
| Vong et al., 2009 | Cambodia | 2006 | Cross-sectional study; case-control  study | A(H5N1) confirmed patient | Zoonotic source known | Rural villagers living near households of A(H5N1) confirmed case | 674 | 21.5 (4 months–89) years-old | Yes (674/0/674) | Animal-to-human transmission | - |
| Wang et al., 2006 | Guangdong, China | 2006 | Cross-sectional study | A(H5N1) confirmed case | Zoonotic source known | Poultry purveyors | 121 | - | Yes (110/0/121) | Animal-to-human transmission | - |
| Cai et al., 2009 | Ruegen island, Germany | 2006 | Cross-sectional study | Animal infection | Zoonotic source known | Local auxiliary fire brigade, local government workers and veterinarians | 97 | 36 (18–60) years-old | Yes (78/0/97) | Animal-to-human transmission | - |
| Wang et al., 2008 | Jiangsu province, China | 2007 | Retrospective cohort study | A(H5N1) confirmed patient | Zoonotic source known | Household members, social contacts and health care workers | 9 household members, 5 social contacts and 77 health care workers | Household members: 25-75 years-old | Yes (household members: unk/unk/9; social contacts: unk/unk/5; health care workers: unk/unk/77) | Animal-to-human transmission; possible human-to-human transmission | A total of 30 paired sera were collected |
| Reed et al., 2014 | Alaska, United State | 2007 | Cross-sectional study | - | - | Rural subsistence bird hunters and their non-hunting family members, urban sport hunters, wildlife biologists and non-exposed population | 237 rural subsistence bird hunters and 229 non-hunting family members, 164 urban sport hunters, 82 wildlife biologists and 204 non-exposed individuals | Rural subsistence bird hunters: 23 (8-69) years-old; non-hunting family members: 20 (5-85) years-old; urban sport hunters: 47 (8-81) years-old; wildlife biologists: 43.5 (22-63) years-old; non-exposed population: 34.5 (6-66) years-old | Yes (rural subsistence bird hunters: 237/0/237; non-hunting family members: 229/0/229; urban sport hunters: 164/0/164; wildlife biologists: 82/0/82; non-exposed population: 204/0/204) | Animal-to-human transmission | Only rural subsistence bird hunters wear rubber gloves while hunting birds: 15 (6%) |
| Cavailler et al., 2010 | Kampong Cham, Cambodia | 2007 | Cross-sectional study | A(H5N1) confirmed patient | Zoonotic source known | Rural residents living in the same villages as confirmed A(H5N1) cases | 700 rural residents | Median: 18 years-old | Yes (rural residents: 700/0/700) | Animal-to-human transmission; possible human-to-human transmission | - |
| Robert et al., 2010 | Indonesia | 2007 | Cross-sectional study | - | - | Poultry farmers | 495 | 29 (IQR: 23-36) years-old | Yes (495/0/495) | Animal-to-human transmission | - |
| Wang et al., 2009 | Guangdong, China | 2007-08 | Cross-sectional study | - | - | Poultry retailer, wholesaler, workers in larger breeding enterprise, farmer, goods retailer (other than poultry) in LPM, swine workers and general population | 252 retailers, 244 wholesalers, 125 enterprise workers, 869 farmers, 182 swine farmers, 218 goods retailers, and 301 non-exposed persons | - | Yes (retailer: 252/0/252; wholesaler: 244/0/244; enterprise workers: 125/0/125; farmer: 869/0/869; swine workers: 182/0/182; goods retailers: 218/0/218; non-exposed person: 301/0/301) | Animal-to-human transmission | - |
| Wallensten et al., 2009 | Dorset, England | 2008 | Cross-sectional study | Animal infection | Zoonotic source known | Exposed populations who were euthanizing sick birds, de-ringing and bagging carcasses | 13 | - | Yes (11/0/13) | Animal-to-human transmission | Full PPE used when confirming A(H5N1) outbreaks in animals |
| Blair et al., 2013 and Gray et al., 2014 | Kampong Cham Province, Cambodia | 2008-10 | Prospective cohort study | A(H5N1) confirmed patient; Animal infection | Zoonotic source known | Rural villagers living in villages where highly pathogenic avian influenza (HPAI) A(H5N1) virus had been reported in humans and poultry from 2006 to 2008 | Baseline: 800; 1-year follow-up: 800; 2-year follow-up: 784 | Median: 39.6 years-old | Yes (baseline: 800/0/800; 1-year follow-up: 0/800/800; 2-year follow-up: 76^*^/708/784) | Animal-to-human transmission | No evidence of the incidence of disease in humans  . |
| Khuntirat et al., 2011 and Krueger et al., 2013 | Kamphaeng Phet province, Thailand | 2008-10 | Prospective cohort study | Animal infection | Zoonotic source known | Rural villagers living in the regions where HPAI poultry outbreaks occurred | Baseline: 800; 1-year follow-up: 768; 2-year follow-up: 784 | 49.6 (20–84) years-old | Yes (baseline: 800/0/800; 1-year follow-up: unk/unk/768; 2-year follow-up: unk/unk/784) | Animal-to-human transmission | At least 747 paired sera were collected from rural villagers during the 1-year and 2-year follow-up. |
| Ly et al., 2016 | Cambodia | 2008-10 | Cross-sectional study; Case-control study | A(H5N1) confirmed patient; Animal infection | Zoonotic source known | Military trainees and rural residents living in the same region as H5-positive poultry | 394 military trainees, 622 rural residents in 2009, and 366 rural residents in 2010 | Military trainees: mainly aged 18-35 years-old; rural residents in 2009: 26.5 (5-70) years-old | Yes (military trainees: 394/0/394; rural residents in 2009: 622/0/622; and rural residents in 2010: 366/0/366 ) | Animal-to-human transmission | 622 and 624 rural residents were reported in ref. 29 and ref. 30. |
| Okoye et al., 2013 | Nigeria | 2008-11 | Prospective cohort study | - | - | Poultry exposed and non-exposed population | 316 poultry exposed and 54 non-exposed individuals | ≥18 years-old | Yes (baseline: poultry exposed population: 316/0/316, non-exposed population: 54/0/54; follow-up visit 1: exposed population: 0/316/0, non-exposed population: 0/54/54; follow-up visit 2: exposed population: 0/316/0, non-exposed population: 0/54/54) | Animal-to-human transmission | - |
| Cao et al., 2013 | Guangdong, Zhejiang, Fujian, Jiangxi, China | 2008-12 | Cross-sectional study | - | - | Exposed swine farm residents (e.g. veterinarian, farm owners, farm employee, spouses and children), non-exposed urban residents | 1606 swine farm residents; 104 urban residents | Swine farm residents (range): 8-70 years-old;  Urban residents: 31 (9-87) years-old | Yes (swine farm residents: 1606/0/1606; urban residents: 104/0/104) | Animal-to-human transmission | - |
| Nasreen et al., 2013 | Dhaka  Bangladesh | 2009 | Cross-sectional study | Animal infection | Zoonotic source known | LPM workers and farm workers | 210 LPM workers and 212 farm workers | LPM workers: 30 (IQR: 24–38) years-old; farm workers: 33 (IQR: 24–45) years-old | Yes (LPM workers: 210/0/210; farm workers: 212/0/212) | Animal-to-human transmission | - |
| Zhang et al., 2011 | Guangdong, China | 2009 | Cross-sectional study | - | - | Blood donors | 200 | Range: 19-55 years-old | Yes (200/0/200) | Animal-to-human transmission | - |
| Nasreen et al., 2015 | Dhaka, Chittagong, Netrokona and Rajshahi, Bangladesh | 2009-10 | Prospective cohort study | Animal infection | Zoonotic source known | LPM workers and nonpoultry workers | 404 and 278 LPM workers at baseline and follow-up visit 1; 101 nonpoultry workers | LPM workers: 28 (IQR: 22–38) years-old  non poultry workers 36 (IQR: 32–40) years-old | Yes (LPM workers at baseline: 404/0/404; LPM workers at follow-up visit 1: 0/278/278; nonpoultry workers: 101/0/101) | Animal-to-human transmission | No evidence of A(H5N1) virus exposure for seropositive LPM workers; PPE was not used by seropositive LPM workers. |
| Coman et al., 2013 | Tulcea, Cluj-Napoca, Romania | 2009-10 | Cross-sectional study | - | - | Agriculture workers (swine workers in large commercial domestic farms and in small traditional backyard farm), non-exposed population | 149 modern swine workers, 163 small traditional backyard farm workers, 51 age-group matched non-exposed controls | ≥20 years-old | Yes (modern swine workers: 149/0/149; small traditional backyard farm workers: 163/0/163, non-exposed population: 51/0/51) | Animal-to-human transmission | Stronger biosecurity measures or better PPE was used in large commercial domestic farms. Only 33 agriculture workers had occupational exposure ^b^. |
| Yu et al., 2013 | Beijing, China | 2009-10 | Surveillance study | - | - | Duck keeper, chicken keeper, and chicken butcher | 155 duck keepers; 114 chicken keepers; 36 chicken butchers | 18-35 years-old: 76 persons; 36-45 years-old: 147 persons; >45 years-old: 82 persons | Yes (duck keeper: 155/0/155; chicken keeper: 114/0/114; chicken butcher: 36/0/36) | Animal-to-human transmission | PPE use was  not a routine practice among poultry workers. |
| Huo et al., 2012 | Jiangsu, China | 2010 | Cross-sectional study | - | - | Backyard poultry farmers | 306 poultry farmers | 58 (IQR: 45.75-64.25) years-old | Yes (306/0/306) | Animal-to-human transmission | Up to 40% of the poultry workers enrolled in study were ≥60 years old. |
| Chen et al., 2011 | Jiangsu, China | 2010 | Cross-sectional study | - | - | Residents in rural areas | 1039 serums collected from rural areas | - | Yes (1039/0/unk) | Animal-to-human transmission | - |
| Pawar et al., 2014 | India | 2009-2012 | Cross-sectional study | - | - | Poultry workers involved in cleaning and disinfection activities during HPAI A(H5N1) virus outbreaks in wild birds, general population | 466 poultry workers, 162 general population | Range: 15-75 years-old | Yes (poultry workers: 466/0/466; general population: 162/0/162) | Animal-to-human transmission | - |
| Ahad et al., 2014 | Khyber Pakhtunkhwa Province, the Federal Area, North, Central and South Punjab, Pakistan | 2010-11 | Cross-sectional study | - | - | Poultry farm workers who were feeding poultry, handling healthy, sick, and dying chickens, collecting eggs and cleaning poultry stalls | 354 poultry farm workers | > 12 years-old | Yes (poultry farm workers: 354/0/354) | Animal-to-human transmission | PPE was not reported; No evidence of A(H5N1) virus infection for subjects with exposure to infected poultry |
| Li et al., 2013 | Zhejiang province, China | 2010-12 | Cross-sectional study | Animal infection | Zoonotic source known | LBM workers, large scale poultry company workers, poultry slaughtering and processing plants workers, backyard poultry farmers | 241 LBM workers, 537 large scale poultry company workers, 36 poultry slaughtering and processing plants workers, 355 backyard poultry farmers | 48 (15-94) years-old | Yes (LBM workers: 241/0/241; large scale poultry company workers: 537/0/537; poultry slaughtering and processing plants workers: 36/0/36; backyard poultry farmers: 355/0/355) | Animal-to-human transmission | PPE use was not reported. |
| Gomaa et al., 2014 | Kafr El Sheikh, Gharbiya, Qalyubiya, Fayyoum, Sharkiya, Cairo, Egypt | 2010-12 | Prospective cohort study | Animal infection | Zoonotic source known | Exposed population from backyard, LPM and commercial farms, and non-exposed population | Baseline: 750 exposed and 250 non-exposed individuals; 1-year follow up: 682 exposed and 139 non-exposed individuals; 2-year follow up: 649 exposed and 104 non-exposed individuals | Exposed population: 27 (2-79) years-old; non-exposed population: 31 (2-76) years-old | Yes (baseline: exposed, 708/0/750, non-exposed, 224/0/250; 1-year follow-up: exposed, 0/682/682, non-exposed, 0/139/139; 2-year follow-up: exposed, 0/649/649, non-exposed, 0/104/104 | Animal-to-human transmission | No evidence of the incidence of disease in humans, or the rate of secondary human-to-human transmission. |
| Dung et al., 2014 | Hanoi, Thaibinh, Thanhhoa, Viet Nam | 2011 | Cross-sectional study | - | - | Poultry sellers, slaughterers, and other poultry workers (breeders, transporters, veterinarians, drivers, feather collectors, cleaners, market managers) | 380 poultry sellers and slaughterers, 227 other poultry workers | 42.3 (18-74) years-old | Yes (poultry sellers and slaughterers: 380/0/380; other poultry workers: 227/0/227) | Animal-to-human transmission | Participating poultry workers may have had more than one poultry-related occupation during their exposure period. No controls were enrolled in this study. PPE use in occupational populations was not reported. |
| Chea et al., 2014 | Cambodia | 2011 | Retrospective cohort study | A(H5N1) confirmed case | Zoonotic source known | Household member, neighbor, HCWs, social contacts | 11 household members, 11 neighbors, 26 HCWs and 15 social contacts | - | Yes (household members: 4/7/11; neighbors: 1/10/11; HCWs: 0/0/26; social contacts: 0/0/15) | Animal-to-human transmission; possible human-to-human transmission | - |
| Chakraborty et al., 2017 | Dhaka, Bangladesh | 2011 | Retrospective cohort study | A(H5N1) confirmed case | Zoonotic source known | Close contacts (including parents and caregivers) | 57 close contacts | - | Yes (unk/unk/57) | Animal-to-human transmission; possible human-to-human transmission | - |
| Su et al., 2013 | Guangdong  China | 2011-12 | Surveillance study | Animal infection | Zoonotic source known | Veterinarians | 406 | Range: 20-65 years-old | Yes (406/0/406) | Animal-to-human transmission | - |
| Shi et al., 2014 | Hunan Province, China | 2011-12 | Cross-sectional study | A(H5N1) confirmed patient | Zoonotic source known | Local staff of the Eastern Dongting Lake Wetland Nature Reserve and on duck farms, and individual duck breeder | 1050 | >18 years-old | Yes (1050/0/1050) | Animal-to-human transmission | - |
| Xiong et al., 2014 | China | 2012 | Surveillance study | - | - | General population (the staff and postgraduate students) | 394 participants | Range: 20–82 years-old | Yes (394/0/394) | Animal-to-human transmission | Serum samples were collected in an academic institute  campus |
| Shimizu et al., 2016 | East Java, Indonesia | 2012-16 | Surveillance study | - | - | LPM workers | 63 LPM workers in 2012, 101 LPM workers in 2014, 100 LPM workers in 2015 and 142 LPM workers in 2016 | - | Yes (63/0/63 in 2012; 76/25/101 in 2014; 36/64/100 in 2015; 84/58/142 in 2016) | Animal-to-human transmission | - |
| Horm et al., 2016 | Cambodia | 2013 | Prospective cohort study | - | - | LBM workers | Baseline: 125; 2^nd^ resampling mission: 117; 3^rd^ resampling mission: 105; 4^th^ resampling mission: 106 | All LBM workers are adult age | Yes (baseline: 125/0/125; 2^nd^ resampling mission: 0/117/117; 3^rd^ resampling mission: 0/105/105; 4^th^ resampling mission: 0/106/106) | Animal-to-human transmission | Participants with PPE use was not reported. |
| Wang et al., 2014 | Guangdong, China | 2013 | Serial cross-sectional study | - | - | Poultry market workers and general population | 876 (501 and 375 at 1st and 2nd survey) poultry market workers, 825 (417 and 408 at 1st and 2nd survey) non-exposed individuals | Poultry market workers: 41 (16–67) years-old | Yes (1st survey: poultry market workers: 501/0/501; non-exposed individuals: 417/0/417; 2nd survey: poultry market workers: 279/96/375; non-exposed individuals: 408/0/408) | Animal-to-human transmission | 96 poultry market workers were recruited for the 1st and 2nd survey. |
| Chen et al., 2015 | Guangdong, China | 2013-14 | Cross-sectional study | - | - | Swine workers ^a^, poultry farm workers ^a^, LPM workers ^a^, veterinarians ^a^, and volunteers | 171 swine workers, 150 poultry farm workers, 105 LPM workers, 120 veterinarians, and 264 volunteers | Swine workers (mean): 43.2 years-old; poultry farm workers (mean): 32.4 years-old; LPM workers (mean): 44.9 years-old; veterinarians (mean): 40.6 years-old; volunteers (mean): 36.7 years-old | Yes (swine workers: 171/0/171; poultry farm workers: 150/0/150; LPM workers: 105/0/105; veterinarians: 120/0/120; volunteers: 264/0/264) | Animal-to-human transmission | - |
| To et al., 2015 | Hong Kong, China | 2013-14 | Prospective cohort study | - | - | LPM workers and slaughterhouse workers | Baseline: 30 LPM workers and 69 slaughterhouse workers; Follow-up visit 1: 45 LPM workers and 27 slaughterhouse workers | Baseline:  LPM workers: 57 (IQR: 53-61) years-old, slaughterhouse workers: 52 (IQR: 49-57) years-old;  Follow-up visit 1:  LPM workers: 56 (IQR: 46-64) years-old, slaughterhouse workers: 52 (IQR: 49-59) years-old | Yes (baseline: LPM workers: 30/0/30, slaughterhouse workers: 69/0/69;  follow-up visit 1:  LPM workers: 35/10/45 slaughterhouse workers: 15/12/27) | Animal-to-human transmission | PPE use in occupational workers was not reported. |
| Ma et al., 2015 | Jiangsu, China | 2013-14 | Retrospective cohort study | A(H5N1) confirmed case | Zoonotic source known | Family members, HCWs, and other contacts in health care setting | 30 family member, 177 HCW, and 18 other contacts in health care setting | Family member: 48.03±17.79 years-old; HCW: 33.71±7.97 years-old; other contacts: 68.50 ±14.89 years-old | Yes (family members: 30/0/30; HCWs: 177/0/177; other contacts in health care setting: 18/0/18) | Possible human-to-human transmission | - |
| Yang et al., 2016 | Beijing, China | 2013-15 | Prospective cohort study | - | - | Poultry workers, swine workers and non-exposed general population | Baseline: 1258 poultry workers, 1332 swine workers and 1200 non-exposed individuals; follow-up visit 1: 1056 poultry workers, 1254 swine workers and 1188 non-exposed individuals; follow-up visit 2: 1123 poultry workers, 998 swine workers and 1135 non-exposed individuals | - | Yes (baseline: poultry workers: 1258/0/1258; swine workers: 1332/0/1332; non-exposed individual: 1200/0/1200; follow-up visit 1: poultry workers: 265/791/1056; swine workers: 392/862/1254; non-exposed individual: 278/910/1188; follow-up visit 2: poultry workers: 611/512/1123; swine workers: 430/568/998; non-exposed individual: 203/932/1135) | Animal-to-human transmission | No evidence of the incidence of disease in humans. |
| Ma et al., 2018 | Jiangsu Province, China | 2013-16 | Prospective cohort study | - | - | Poultry workers, swine workers, and general population control | Baseline: 511 poultry workers, 569 swine workers and 915 general population control; 1-year follow-up: 533 poultry workers, 589 swine workers and 881 general population controls; 2-year follow-up: 535 poultry workers, 501 swine workers, and 855 general population controls; 3-year follow-up: 491 poultry workers, 367 swine workers and 785 general population controls | Baseline:  Poultry workers: 46.8 ± 11.3 years-old; swine workers: 45.4 ± 10.4 years-old; and general population control: 41.6 ± 11.0 years-old;  1-year follow-up:  Poultry workers: 48.6 ± 11.1 years-old; swine workers: 46.5 ± 11.3 years-old; and general population control: 42.6 ± 10.8 years-old;  2-year follow-up:  Poultry workers: 47.9 ± 11.4 years-old; swine workers: 47.0 ± 11.6 years-old; and general population control: 42.9 ± 10.6 years-old;  3-year follow-up:  Poultry workers: 49.4 ± 10.9 years-old; swine workers: 48.0 ± 10.2 years-old; and general population control: 44.1 ± 10.5 years-old | Yes (baseline: poultry workers: 511/0/511, swine workers: 569/0/569, general population control: 915/0/915; 1-year follow-up: poultry workers: 239/294/533, swine workers: 295/294/589, general population control: 332/549/881; 2-year follow-up: poultry workers: 166/369/535, 569 swine workers: 161/340/501, general population control: 276/579/855; 3-year follow-up: poultry workers: 48/443/493, 569 swine workers: 54/313/367, general population control: 22/763/785) | Animal-to-human transmission | Cross-reactivity between A(H5N1) and seasonal influenza A viruses was not explored. |
| Ly et al., 2017 | Kratie Province, Kompong Cham Province, Cambodia | 2014 | Prospective cohort study | A(H5N1) confirmed patient | Zoonotic source known | Rural residents living in the same region as suspected and confirmed cases | 695 rural residents in Kratie, and 921 rural residents in Kompong Cham | - | Yes (0/881/1616) | Animal-to-human transmission; possible human-to-human transmission | No evidence of the incidence of disease in humans |
| Quan et al, 2019 | China | 2014-16 | Prospective cohort study | - | - | Poultry workers and general population | 1407 poultry workers and 216 general population (outpatients with noninfectious disease upon physical examination) | Baseline:  Poultry workers: 46 (IQR: 38-52) years-old; general population: 48 (IQR: 34-59) years-old;  Follow-up visit 1:  Poultry workers: 47 (IQR: 38-52) years-old;  Follow-up visit 2:  Poultry workers: 45 (IQR: 35-52) years-old;  Follow-up visit 3:  Poultry workers: 45 (IQR: 35-52) years-old | Yes (poultry workers: 755/652/1407; general population: 216/0/216) | Animal-to-human transmission | No evidence of the incidence of disease in humans |
| Sirawan et al., 2020 | Lebanon | 2017 | Cross-sectional study | - | - | Poultry worker | 69 farmer workers | >18 years-old | Yes (69/0/69) | Animal-to-human transmission | - |

Abbreviations: Exposed health care workers (HCWs) who were exposed to the A(H5N1) index case patient and who worked on the ward where the index case patient was hospitalized; Non-exposed HCWs, health care workers without exposure worked at the same hospital but had not worked on the ward where the index case patient was hospitalized; GWs, government workers who participated in wide slaughter of chickens and other fowls; PWs: poultry workers who routinely were expected to have the highest level of exposure to A(H5N1) virus-infected birds (e.g. poultry breeders, transporters and drivers, sellers, slaughterers veterinarians, feather collectors, cleaners, or market managers); PPE, personal protective equipment; LPM: live poultry market; LBM: live bird market; unk: unknown; IQR: inter-quantile range; ICU: intensive care unit.

^a^ Exposed participants indicated they worked in close contact with pigs, poultry, or dogs for more than 8 h per day, 5 days a week.

^b^ Exposure to domestic poultry, wild birds, or pigs as part of daily activities for ≥5 cumulative h/wk.

^*^77 replacement enrollments were added after 1-year follow-up.

^**^Epidemiologists, public health officials, and media reporters.

^***^Residents living in the region where A(H5N1) outbreaks in human, poultry, or both during the study periods.

^†^Individuals who lived with an A(H5N1) virus-infected person for part or during all of the case patient’s infectious period.

Table S3. Summary of antibody detection assays to identify human infection with highly pathogenic avian influenza A(H5N1) virus among sixty-five serological studies included in systematic review

| **Reference** | **Paired serums** | **Days from last possible exposure to sampling (median, range)** | **Laboratory test method used for confirmation of A(H5N1) virus infection** | | | | | | | | | **Laboratory test method for confirmation prior seasonal influenza A virus infections** | | | | | | | | **Comment** |
| --- | --- | --- | --- | --- | --- | --- | --- | --- | --- | --- | --- | --- | --- | --- | --- | --- | --- | --- | --- | --- |
|  |  |  | **Assay methods (screening methods/confirmatory methods)** | **Antibodies measured** | **RBCs type** | **Serum dilution** | **A(H5N1) virus antigens used** | **Clade(s)/Genotype** | **Current circulating strains/**  **clades** | **Reported positive cut-off value** | **Criteria for seroconversions** | **Antigens used for testing prior seasonal influenza A virus infections** | **Subtypes/lineages** | **Current circulating seasonal influenza virus strains** | **Assay methods used for testing prior seasonal influenza A virus infections** | **RBCs type used for testing prior seasonal influenza A virus infections** | **Positive cut-off**  **value to test prior seasonal influenza A virus infections** | **Criteria used to confirm prior seasonal influenza A virus infections** | **Cross- reactivity**  **(Yes/No)** |  |
| Katz et al., 1999 | Yes | 1^st^: <11;  2^nd^: >21 | MN, ELISA/ WB | Neutralizing antibodies (NT) | - | - | A/Hong Kong/156/97  A/Duck/Singapore-Q/F119-3/97 (H5N3) | 0/GsGD | - | NT≥1:80, confirmed by WB or ELISA (≥1600) | NTP | - | - | - | - | - | - | - | - | 1. WB H5 protein:  A/HongKong/156/97 virus  2. ELISA for age ≤14 years-old |
| Bridges et al., 2000 | Yes | 11-51 days | MN/WB | Neutralizing antibodies (NT) | - | - | A/Hong Kong/156/97  A/duck/Singapore/ -Q/F119-3/97 (H5N3) | 0/GsGD | - | NT≥1:80, confirmed by WB | - | - | - | - | - | - | - | - | - | WB H5 protein:  A/Hong Kong/156/97 virus; |
| Bridges et al., 2002 | Yes | - | MN/WB | Neutralizing antibodies (NT) | - | - | A/Hong Kong/156/97  A/Duck/Singapore/-Q/ F119-3/97 (H5N3) | 0/GsGD | - | NT≥1:80, confirmed by WB | - | - | - | - | - | - | - | - | - | Persons ≥60 or ≤14 years old and those who did not report their age were excluded for less specific of WB and MN. |
| Uyeki et al., 2012 | No | - | MN/WB | Neutralizing antibodies (NT) | - | 1:10 | A/Goose/Vietnam/113/2001^*^  A/HK/213/03^*^  A/HK/156/97^*^ | 0  1/Z  0/GsGD | - | NT≥1:40, confirmed by WB | - | - | - | - | - | - | - | - | - | WB H5 protein**^η^**:  A/HK/156/97 |
| Kwon et al., 2012 | unknown | The same day of culling operation | MN/HI, WB | Neutralizing antibodies (NT) and HAI antibodies | horse | - | A/chicken/Korea/ES/03* | 2.5/V | - | NT≥1:80, confirmed by HI or WB | NTP | - | - | - | - | - | - | - | - | WB H5 protein: A/Vietnam/1203/2004 |
| Liem et al., 2005 | No | - | MN/WB | Neutralizing antibodies (NT) | - | - | A/Vietnam/1194/2004  A/Vietnam/3212/2004 | 1/Z  1/Z | - | NT≥1:40, confirmed by WB | - | - | - | seasonal H1N1 | MN (absorption test) | - | - | >4-fold reduction | Yes | WB H5 protein:  A/HK/156/97 virus |
| Schultsz et al., 2005 | Yes | 1^st^: 7 (2-12);  2^nd^: 21 (17-26) | ELISA/MN | Neutralizing antibodies (NT) | - | - | - | - | - | unknown | NTP | Influenza A(H1N1) virus  Influenza A(H3N2) virus | unk  Seasonal influenza A(H3N2) virus | - | HAI  MN | - | - | >4-fold increase | No | - |
| Lu et al., 2008 | No | - | HAI/MN | Neutralizing antibodies (NT) and HAI antibodies | chicken | 1:5 | A/goose/Guangdong/1/96^#^ | 0/GsGD | - | HAI≥1:20, NT≥1:20 | - | A/New Caledonia/20/99  A/Panama/2007/99 | Seasonal influenza A(H1N1) virus  Seasonal influenza A(H3N2) virus | - | HAI | chicken | ≥1:20 | - | Yes | - |
| Hinjoy et al., 2008 | No | - | MN | Neutralizing antibodies | - | - | - | - | - | unknown | - | - | - | - | - | - | - | - | - | Persons >50 years of age were excluded from laboratory analysis |
| Apisarnthanarak et al., 2004 | Yes | 3 (2–3) | MN/WB | Neutralizing antibodies (NT) | - | 1:20 | A/Thailand/16/2004 | 1/Z | - | NT≥1:80, confirmed by WB | - | - | - | - | - | - | - | - | - | - |
| Khuntirat et al., 2015 | Yes | - | MN | Neutralizing antibodies (NT) | - | 1:10 | A/Thailand/676/2005^*^  A/Thailand/384/2006 | 1/Z  1/Z | - | NT≥1:10 | - | - | - | - | - | - | - | - | - | - |
| Vong et al., 2006 | No | ≈2 months | MN/WB | Neutralizing antibodies | - | - | - | 1 | 1 | NT≥1:80, confirmed by WB | - | - | - | - | - | - | - | - | - | Antigens used were similar to circulating virus (both antigenic and genetic) |
| Dejpichai et al., 2009 | No | - | MN/IF-based MN | Neutralizing antibodies | - | - | A/Thailand/1(KAN-1)/2004^*^ | 1/Z | - | NT≥1:40, confirmed by IF | - | - | - | - | - | - | - | - | - | - |
| Santhia et al., 2009 | No | 18 months | MN | Neutralizing antibodies | - | - | - | - | - | NT≥1:80 | - | - | - | - | - | - | - | - | - | - |
| Schultsz et al., 2009 | No | 164 (134-262 | MN/HAI | Neutralizing antibodies (NT) and HAI antibodies | horse | - | A/Vietnam/1194/2004  A/Vietnam/30850/05  A/Vietnam/3212/2004  A/VN/CL26/2004 | 1/Z  2.3.4/Z  1  1 | 1 | NT≥1:80,  HAI≥1:80 | - | Influenza A(H1N1) virus  Influenza A(H3N2) virus | unk  Seasonal influenza A(H3N2) virus | - | MN (absorption test) | - | - | >4-fold reduction | Yes | - |
| Apisarnthanarak et al., 2006 | Yes | 1^st^: <1 week  2^nd^: >14 days | MN/IF-based MN | Neutralizing antibodies (NT) | - | 1:20 | A/Thailand/1(KAN-1)/2004 | 1/Z | - | NT≥1:80, confirmed by IF | NTP | - | - | - | - | - | - | - | - | Adults ≥60 years of age were excluded from the serologic tests |
| Buchy et al., 2007 | No | - | MN/WB | Neutralizing antibodies (NT) | - | - | - | 1/Z | 1/Z | NT≥1:80, confirmed by WB | - | - | - | - | - | - | - | - | - | - |
| Liao et al., 2013 | Yes | Single:  43 (IQR: 29–70);  Paired:  1^st^: 4 (IQR: 2–7);  2^nd^: 57 (IQR: 30–89) | HAI/MN | Neutralizing (NT) antibodies and HAI antibodies | horse | - | A/Anhui/1/2005  A/CK/HN/21/05 | 2.3.4/Z  - | - | HAI≥1:40, NT (adult)≥1:80, NT (≤14 years-old)≥1:40 | ≥4-fold rise | - | - | seasonal A(H1N1) and seasonal A(H3N2) viruses | MN (absorption test) | - | - | >4-fold reduction | No | NT≥1:40 used for children aged ≤14 yrs |
| Ilyicheva et al., 2013 | No | - | HAI/MN | Neutralizing (NT) antibodies and HAI antibodies | horse | 1:10 | A/Commongull/Chany/06* | 2.2 | 2.2 | NT≥1:80,  HAI≥1:40 | - | - | - | - | - | - | - | - | - | - |
| Ceyhan et al., 2010 | Yes | - | HAI, ELISA/MN | Neutralizing (NT) antibodies and HAI antibodies | chicken | 1:10 | A/Turkey/13/06* | 2.2/Z | A/Turkey/Turkey/1/2005 | HAI≥1:20 or ELISA positive, then confirmed by NT (≥1:10) | - | - | - | - | - | - | - | - | - | - |
| Ortiz et al., 2007 | No | - | MN/HAI | Neutralizing (NT) antibodies and HAI antibodies | horse | - | A/chicken/Nigeria/246/06^*^  A/chicken/Nigeria/42/2006^*^ | 2.2/Z | - | NT≥1:80, confirmed by  HAI | - | A/New York/ 55/2005 | Seasonal influenza A(H3N2) virus | A/New York/55/2005 | MN | - | ≥1:80 | - | Yes | Persons >59 years were excluded for testing;  Positive cut-off value of HAI is unknown |
| Vong et al., 2009 | No | - | MN/HI, WB | Neutralizing (NT) antibodies and HAI antibodies | horse | - | A/Vietnam/JP/14/2005* | 1/Z | - | NT≥1:80, confirmed by WB | - | - | - | - | - | - | - | - | - | WB H5 protein:  clade 1 recombinant HA antigen: A/Vietnam/1203/2004 |
| Wang et al., 2006 | No | - | HAI/NT | Neutralizing (NT) antibodies and HAI antibodies | turkey | - | A/Hong Kong/486/97  A/Vietnam/1194/2004 | 0/GsGD  1/Z | A/Guangzhou/1/2006 | unknown | - | - | - | - | - | - | - | - | - | - |
| Cai et al., 2009 | No | - | PN/MN | Neutralizing (NT) antibodies | - | - | A/whooper swan/R652/Germany/2006^#^  A/bar-headed goose/Qinghai/1A/2005^#^  A/whooper swan/ Mongolia/244/2005^*^ | 2.2/Z  2.2.1/Z  2.2/Z | - | NT >1:20,  NT >1:20 | - | A/New Caledonia/20/99  A/Wisconsin/67/05 | Seasonal influenza A(H1N1) virus  Seasonal influenza A(H3N2) virus | - | MN | - | - | - | Yes | - |
| Wang et al., 2008 | No | - | MN/HAI | Neutralizing (NT) antibodies and HAI antibodies | horse | - | A/Jiangsu/1/2007^*^ | 2.3.4/Z | 2.3.4 | NT≥1:80, HAI: unknown | NT≥1:80 or ≥4-fold rise | - | - | - | - | - | - | - | - | - |
| Reed et al., 2014 | No | - | MN/HAI, WB | Neutralizing (NT) antibodies and HAI antibodies | horse | 1:10 | A/Whooper swan/Mongolia/244/2005 | 2.2.1/Z | - | NT≥1:40, confirmed by  HAI (≥1:80) and WB | - | - | - | - | - | - | - | - | - | WB H5 protein:  clade 2.1 recombinant HA protein based on A/Indonesia/05/2005 virus |
| Cavailler et al., 2010 | No | - | H5pp-based MN/HAI, MN | Neutralizing (NT) antibodies and HAI antibodies | horse | 1:10 | A/Cambodia/R0405050/2007^*^ | 1.1.1/Z | - | NT≥1:160,  HAI≥1:80, | - | - | - | - | - | - | - | - | - | - |
| Robert et al., 2010 | No | - | HAI/NT | Neutralizing (NT) antibodies and HAI antibodies | - | - | A/Ck/Banten/05-1116/05  A/H5N1/Indo/05/IBCDC-RG virus | 2.1/Z  2.1/Z | - | HAI≥1:160,  NT≥1:80 | - | - | - | - | - | - | - | - | - | - |
| Wang et al., 2009 | No | - | NT | Neutralizing (NT) antibodies | - | - | A/Vietnam/1194/2004  A/Hong Kong/486/1997 | 1/Z  0/GsGD | - | unknown | - | - | - | - | - | - | - | - | - | - |
| Wallensten et al., 2009 | No | After 30 days | MN/HAI | Neutralizing (NT) antibodies and HAI antibodies | horse | MN: 1:20;  HI: 1:8 | A/turkey/Turkey/1/2005 | 2.2 | A/mute swan/England  /26/2008 | unknown | - | - | - | - | - | - | - | - | - | - |
| Blair et al., 2013 | No | - | MN | Neutralizing (NT) antibodies | - | 1:10 | A/Cambodia/R0404050/2007 | - | - | NT≥1:80 | - | A/New Caledonia/20/99  A/Brisbane/59/2007  A/Panama/2007/99  A/Brisbane/10/2007  A/Pandemic/Mexico/4108/2009 | Seasonal influenza A(H1N1) virus  Seasonal influenza A(H1N1) virus  Seasonal influenza A(H3N2) virus  Seasonal influenza A(H3N2) virus  Influenza A(H1N1)pdm09 virus | - | HAI | guinea pig /turkey | ≥1:40 | - | Yes | - |
| Gray et al., 2014 | Yes | - | MN | Neutralizing (NT) antibodies | - | 1:10 | A/Cambodia/R0404050/2007 | - | - | NT≥1:80 | - | A/Brisbane/59/2007  A/Mexico/4108/2009  A/Brisbane/10/2007 | Seasonal influenza A(H1N1) virus  Influenza A(H1N1)pdm09 virus  Seasonal influenza A(H3N2) virus | - | HAI | guinea pig /turkey | ≥1:40 | - | Yes | - |
| Khuntirat et al., 2011 | No | - | MN | Neutralizing (NT) antibodies | - | 1:10 | A/Thailand/676/2005^*^  A/Thailand/384/2006 | 1/Z  1/Z | -  - | NT≥1:10 | - | A/New Caledonia/20/99  A/Brisbane/59/2007  A/Panama/2007/99  A/Brisbane/10/2007  A/Mexico/4108/2009 | Seasonal influenza A(H1N1) virus  Seasonal influenza A(H1N1) virus  Seasonal influenza A(H3N2) virus  Seasonal influenza A(H3N2) virus  Influenza A(H1N1)pdm09 virus | - | HAI | guinea pig /turkey | ≥1:40 | - | Yes | - |
| Krueger et al., 2013 | Yes | - | MN | Neutralizing (NT) antibodies | - | 1:10 | A/Thailand/676/2005^*^  A/Thailand/384/2006 | 1/Z  1/Z | - | NT≥1:10 | - | A/Mexico/4108/2009  A/Brisbane/59/2007  A/Brisbane/10/2007 | Influenza A(H1N1)pdm09 virus  Seasonal influenza A(H1N1) virus  Seasonal influenza A(H3N2) virus | - | HAI | - | ≥1:40 | ≥4-fold increase | Yes | - |
| Ly et al., 2016 | No | - | H5pp-based MN, MN/WB, HAI, MN | Neutralizing (NT) antibodies and HAI antibodies | horse | - | A/Cambodia/R0405050/2007^*^  unk  unk | 1.1.1/Z  1.1.1  1.1.2 reassortant | - | NT≥1:80,  HAI≥1:160, or confirmed by WB | - | - | - | - | - | - | - | - | - | Sera from 2006 was tested by MN, and confirmed by WB |
| Okoye et al., 2013 | No | - | MN | Neutralizing (NT) antibodies | - | 1:10 | A/Chicken/Nigeria/2007/1132123 | - | - | NT≥1:10 | - | A/Brisbane/59/2007  A/Mexico/4108/2009  A/Brisbane/10/2007 | Seasonal influenza A(H1N1) virus  Influenza A(H1N1)pdm09 virus  Seasonal influenza A(H3N2) virus | - | HAI | - | ≥1:40 | - | Yes | - |
| Okoye et al., 2014 | Yes | - | MN | Neutralizing (NT) antibodies | - | 1:10 | A/Chicken/Nigeria/2007/1132123 | - | - | HAI≥1:10 | NTP | A/Brisbane/59/2007  A/Mexico/4108/2009  A/Brisbane/10/2007 | Seasonal influenza A(H1N1) virus  Influenza A(H1N1)pdm09 virus  Seasonal influenza A(H3N2) virus | - | HAI | guinea pig /turkey | - | - | Yes | - |
| Cao et al., 2013 | No | - | HAI/MN | Neutralizing (NT) antibodies and HAI antibodies | horse | - | A/chicken/Guangdong/178/04^*^ | 2.3.2 | 2.3.2 | NT≥1:80,  HAI≥1:80 | - | unk | unk | unk | HAI | - | - | - | Yes | - |
| Nasreen et al., 2013 | No | 444 (22–543) | MN/HAI, WB | Neutralizing (NT) antibodies and HAI antibodies | horse | 1:10 | A/Bangladesh/207095/2008 | 2.2.2/Z | - | NT≥1:40, confirmed by HAI or WB | - | - | - | - | - | - | - | - | - | WB H5 protein:  clade 2.2 recombinant H5 virus;  Positive cut-off value of HI is unknown |
| Zhang et al., 2011 | No | - | MN/HAI, ELISA | Neutralizing (NT) antibodies and HAI antibodies | horse | - | - | 1/Z | - | NT≥1:40,  HAI≥1:40,  ELISA | - | A/California/07/2009  A/Hongkong/8/68 | Influenza A(H1N1)pdm09 virus  Seasonal influenza A(H3N2) virus | - | HAI  MN | - | HAI≥1:40  NT≥1:40 | - | Yes | A dilution of ≥20 was considered a positive result for ELISA. |
| Nasreen et al., 2015 | Yes | - | MN/WB | Neutralizing (NT) antibodies | - | 1:10 | A/Bangladesh/3233/2011^*^ | 2.2.2.1 | A/Bangladesh/3233/20112011 | NT≥1:40, confirmed by WB | ≥4-fold rise | A/ Mexico/4108/2009 | Influenza A(H1N1)pdm09 virus | pandemic A(H1N1) | MN, HAI | - | - | - | - | WB H5 protein:  A/bar-headed goose/Qinghai/1A/2005 |
| Coman et al., 2013 | No | - | MN | Neutralizing (NT) antibodies | - | 1:10 | A/Chicken/Romania/6059-1TS/2008 | - | - | NT≥1:10 | - | A/Brisbane/59/2007  A/New Caledonia/20/1999  A/Mexico/4108/2009  A/Brisbane/10/2007 | Seasonal influenza A(H1N1) virus  Seasonal influenza A(H1N1) virus  Influenza A(H1N1)pdm09 virus  Seasonal influenza A(H3N2) virus | - | HAI | guinea pig /turkey | ≥1:40 | - | Yes | - |
| Yu et al., 2013 | No | - | MN | Neutralizing (NT) antibodies | - | - | A/duck/Huabei/01/2007 | 2.3.4 | 2.3.4 | NT≥1:80 | - | - | - | - | - | - | - | - | - | - |
| Huo et al., 2012 | No | - | HAI | HAI antibodies | horse | 1:10 | A/Anhui/1/05^#^  A/Hubei/1/10^#^ | 2.3.4/Z  2.3.2.1/Z | - | HAI≥1:160 | - | - | - | - | - | - | - | - | - | - |
| Chen et al., 2011 | No | - | HAI | HAI antibodies | - | - | A/Shenzhen/406H/2006  A/BGs/Qinghai/15C/2005 | 2.3.4  2.2 | - | unknown | - | - | - | - | - | - | - | - | - | - |
| Pawar et al., 2014 | No | - | HAI/MN | Neutralizing (NT) antibodies and HAI antibodies | - | - | A/chicken/India/NIV33487/06RG-2008  A/crow/India/NIV1117307/2012  A/Hubei/1/2010/H5N1-RG30 | 2.2  2.3.2.1  2.3.2.1 | - | unknown | - | - | - | - | - | - | - | - | - | - |
| Ahad et al., 2014 | No | - | HAI | HAI antibodies | horse | 1:10 | A/Ck/Scot/59 | - | - | HAI≥1:160 | - | - | - | - | - | - | - | - | - | - |
| Li et al., 2013 | No | - | HAI/MN | Neutralizing (NT) antibodies and HAI antibodies | horse | 1:10 | A/Hubei/1/2010**^η^**  A/Anhui/1/2005**^η^** | 2.3.2.1/Z  2.3.4/Z | - | HAI≥1:160,  NT: unknown | - | - | - | - | - | - | - | - | - | - |
| Gomaa et al., 2014 | Yes | - | MN/HAI | Neutralizing (NT) antibodies and HAI antibodies | horse | 1:10 | A/duck/Egypt/M2583A/2010^*^ | 2.2.1 | 2.2.1 | NT≥1:80,  HAI≥1:80 | - | A/California/04/09  A/Brisbane/10/07 | Influenza A(H1N1)pdm09 virus  Seasonal influenza A(H3N2) virus | - | HAI | turkey | - | - | Yes | - |
| Dung et al., 2014 | No | - | HAI/MN | Neutralizing (NT) antibodies and HAI antibodies | horse | 1:10 | A/Viet Nam/HN30408/2005^*^  A/Viet Nam/HN31244/2007^*^  A/Viet Nam/CM32/2011^*^ | 1  2.3.4  2.3.2.1 | 1  2.3.4  2.3.2.1 | HAI≥1:80,  NT≥1:20 | - | - | - | - | - | - | - | - | - | - |
| Chea et al., 2014 | Yes | - | HAI/MN | Neutralizing (NT) antibodies and HAI antibodies | horse | - | A/Cambodia/V0219301/20111^*^ | 1.1/lineage6 | - | NT≥1:80,  HAI≥1:160 | - | - | - | - | - | - | - | - | - | - |
| Chakraborty et al., 2017 | Yes | Paired  15 days  3 weeks | MN/HAI | Neutralizing (NT) antibodies and HAI antibodies | horse | 1:10 | A/Bangladesh/3233/2011^*^  A/crow/Bangladesh/1061/2011^*^ | 2.2.2.1  2.3.2.1a | 2.2.2.1  2.3.2.1a | NT≥1:40,  HAI: unknown | ≥4-fold rise | - | - | - | - | - | - | - | - | - |
| Su et al., 2013 | No | - | HAI/MN | Neutralizing (NT) antibodies and HAI antibodies | horse | - | A/chicken/Guangdong/178/04**^η^** | 2.3.2 | 2.3.2 | HAI≥1:80,  NT≥1:80 | - | - | - | - | - | - | - | - | - | - |
| Shi et al., 2014 | No | - | HAI/MN | Neutralizing (NT) antibodies and HAI antibodies | horse | - | A/Anhui/1/2005  A/domestic duck/Yueyang/C0816/  2012 | 2.3.4/Z  2.3.2.1 | - | HAI≥1:20, confirmed by MN | - | - | - | - | - | - | - | - | - | Positive cut-off value of MN assay is unknown |
| Xiong et al., 2014 | No | - | HAI | HAI antibodies | chicken | 1:20 | A/Chicken/Hunan/246/2012* | 2.3.2.1 | - | HAI≥1:40 | - | A/New Caledonia/20/1999  A/California/07/2009 | Seasonal influenza A(H1N1) virus  Influenza A(H1N1)pdm09 virus | - | HAI | chicken | ≥1:40 | - | Yes |  |
| Shimizu et al., 2016 | Yes | - | HAI/MN | Neutralizing (NT) antibodies and HAI antibodies | chicken | - | A/turkey/East Java/Av154/2013^*^  A/chicken/East Java/Av240/2014 | 2.3.2.1  2.1.3.3 | 2.3.2.1 | HAI≥1:32,  MN | ≥4-fold rise | A/East Java/D264/2015;  A/Sydney/5/1997 | Influenza A(H1N1)pdm09 virus  Seasonal influenza A(H3N2) virus | pandemic A(H1N1)  seasonal A(H3N2) | HAI | - | - | - | No | Positive cut-off value of MN assay is unknown |
| Horm et al., 2016 | Yes | - | HAI/MN | Neutralizing (NT) antibodies and HAI antibodies | - | - | A/Cambodia/X0121311/2013^*^  A/Cambodia/X0125302/2013^*^ | 1.1.2 reassortant | - | NT≥1:40,  HAI≥1:80 | ≥positive cut-off for both HAI and MN | - | - | - | - | - | - | - | - | - |
| Wang et al., 2014 | Yes | - | HAI | HAI antibodies | horse | 1:20 | A/Shenzhen/01/2011 | - | - | HAI≥1:160 | - | - | - | - | - | - | - | - | - | - |
| Chen et al., 2015 | No | - | HAI | HAI antibodies | horse | 1:10 | A/duck/Anhui/1/2006 | 2.3.4 | 2.3.4 | HAI≥1:40 | - | A/Guangdong/1057/2010  A/California/04/2009  A/New Caledonia/20/99  A/Brisbane/10/2007 | Influenza A(H1N1)pdm09 virus  Influenza A(H1N1)pdm09 virus  Seasonal influenza A(H1N1) virus  Seasonal influenza A(H3N2) virus | - | HAI | horse | ≥1:40 | - | No | Clade 2.3.4 (circulated in mainland of China in recent years) |
| To et al., 2015 | Yes | - | HAI | HAI antibodies | turkey | 1:10 | A/Vietnam/1194/2004 | 1/Z | - | HAI≥1:40 | ≥4-fold rise | A/Hong Kong/415742/2009  A/HK/460611/2013 | A/California/7/2009 (H1N1) pdm09-like virus  A/Switzerland/9715293/2013 (H3N2)-like virus | - | HAI | guinea pig /turkey | - | >4-fold increase | No | - |
| Ma et al., 2015 | No | - | HAI | HAI antibodies | horse | - | A/Anhui/1/2005 | 2.3.4/Z | - | HAI≥1:80  NT: unknown | - | A/California/07/2009  A/Victoria/210/2009 | Influenza A(H1N1)pdm09 virus  Seasonal influenza A(H3N2) virus | - | HAI | turkey | - | - | Yes | - |
| Yang et al., 2016 | Yes | - | HAI | HAI antibodies | horse | 1:10 | A/Anhui/01/2005^#^  A/Hubei/1/2010 | 2.3.4/Z  2.3.2.1/Z | - | HAI≥1:80 | ≥4-fold rise and with a titer ≥ 1:40 for the 2^nd^ specimen | - | - | - | - | - | - | - | - | - |
| Ma et al., 2018 | Yes | - | HAI/MN | Neutralizing (NT) antibodies and HAI antibodies | - | 1:10 | A/chicken/Jiangsu/WX927/2013 | 2.3.2.1c | 2.3.2.1c | HAI: unk,  NT≥1:80 | ≥4-fold rise | - | - | - | - | - | - | - | - | Sera was first screened by HAI at 1:10 |
| Ly et al., 2017 | Yes | ≤1 month | HAI/MN | Neutralizing (NT) antibodies and HAI antibodies | - | - | - | 1.1.2 reassortant | - | HAI≥1:80, NT≥1:40 | NTP/≥4-fold rise | - | - | - | - | - | - | - | - | - |
| Quan et al, 2019 | Yes | - | HAI/MN | Neutralizing (NT) antibodies and HAI antibodies | chicken | 1:10 | A/chicken/Shanghai/02.12 HZ199-P/2015  A/pigeon/Sichuan/NCXN29/2014 | 2.3.2.1 c  2.3.4.4 | 2.3.2.1 c  2.3.4.4 | HAI≥1:20, NT≥1:20 | HAI ≥4-fold rise plus NT ≥1:20 | A/California/04/2009  A/Beijing/CAS0001/2007 | Influenza A(H1N1)pdm09 virus  Seasonal influenza A(H3N2) virus | - | HAI, MN | Chiken | HI≥1:40, MN≥1:80 | - | Yes | - |
| Sirawan et al., 2020 | No | - | MN | Neutralizing (NT) antibodies | - | - | - | 2.3.2.1 | 2.3.2.1 c | NT≥1:10 | - | - | - | - | - | - | - | - | - | - |

Abbreviations: HAI, Hemagglutination inhibition assay; MN, Microneutralization assay; NT, neutralization assay; PN, Plaque neutralization assay; WB, Western blot; IF, Immunofluorescence assay; ELISA, Enzyme-linked immunosorbent assay; IQR: inter-quantile range; WHO, World Health Organization.

NTP (negative to positive): the serum was converted from negative to positive between the 1^st^ and 2^nd^ sampling (refer to their own seropositive definition).

≥4-fold rise: Patients who had detectable antibodies in the first serum sample and a ≥4-fold rise in antibody titer for the second sample, with the second sample achieving a positive titer.

**^*^**Local A(H5N1) virus circulating in poultry (isolated from confirmed case or contaminated environment) or virus with similar antigenicity to local A(H5N1) virus circulating in poultry

**^#^**Reference antigen or vaccines seed virus or standard diagnostic antigen

**^η^**Provided by universities, institutions, CDC or purchased from company

Table S4. Data describing seroprevalence of antibodies to highly pathogenic avian influenza A(H5N1) virus, prior seasonal influenza vaccination and infections, risk factors for A(H5N1) virus infections

| **Reference** | **Study period** | **Study population** | **No. of positive/total no. of participants provided sera (seroprevalence rate, %)** | **Seropositive rate against pandemic or seasonal influenza A viruses (%)** | **Seasonal influenza vaccination (%)** | **Risk factors for A(H5N1) virus infections (OR, 95%CI)** |
| --- | --- | --- | --- | --- | --- | --- |
| Katz et al., 1999 | 1997 | Household contacts  Non-household contacts,  Tour group members  Exposed co-workers  Non-exposed co-workers | 6/51 (11.8)  0/9 (0)  1/26 (3.8)  0/23 (0)  0/24 (0) | - | - | - |
| Bridges et al., 2000 | 1997 | Exposed HCWs  Non-exposed HCWs | 8/217 (3.7)  2/309 (0.6) | - | - | - |
| Bridges et al., 2002 | 1997-98 | GWs  PWs | 9/293 (3.1)  81/1525 (5.3) |  | - | Work in retail vs. wholesale/ hatchery/farm/other poultry industry: 2.7 (1.5-4.9)^a^;  >10% Mortality among poultry: 2.2 (1.3-3.7)^a^;  Butchering poultry: 3.1 (1.6-5.9)^a^;  Feeding poultry: 2.4 (1.4-4.1)^a^;  Handling money: 1.6 (1.0-2.5)^a^;  Preparing poultry for restaurants: 1.7 (1.1-2.7)^a^ |
| Uyeki et al., 2012 | 2001 | LPM workers  Non-exposed population | 8/200 (4.0)  2/200 (1.0) | - | - | - |
| Kwon et al., 2012 | 2003 | Poultry farm workers and their household members  Poultry cullers^†^  Other persons ^**^ | 0/176 (0)  9/1327 (0.7)  0/70 (0) | - | - | - |
| Liem et al., 2005 | 2003-04 | Exposed HCWs | 0/83 | - | 68.3% reported receiving influenza vaccine in 2004 | - |
| Schultsz et al., 2005 | 2004 | HCWs | 0/60 (0) | None of the paired samples from 4 healthcare workers showed 4-fold or greater changes in titer in H1 and H3. | - | - |
| Lu et al., 2008 | 2004 | Occupational population^*^  General citizen population^*^ | Cut-off (1:20): 7/231 (3.0)  Cut-off (1:160): 0/231 (0)  Cut-off (1:20): 23/983 (2.3)  Cut-off (1:160): 0/983 (0) | Cut-off (1:40):  A(H1N1): 222/1214 (18.3)  A(H3N2): 722/1214 (59.5) | - | - |
| Hinjoy et al., 2008 | 2004 | Poultry farmers | 0/322 (0) | - | - | - |
| Apisarnthanarak et al., 2004 | 2004 | Exposed HCWs  Non-exposed HCWs | 0/25 (0)  0/24 (0) | - | - | - |
| Khuntirat et al., 2015 | 2004-07 | Children^*^ | Baseline: 0/251 (0);  1-year follow-up: 0/251 (0)  2-year follow-up: 0/251 (0) | - | - | - |
| Vong et al., 2006 | 2005 | Rural residents^*^ | 0/351 (0) | - | - | - |
| Dejpichai et al., 2009 | 2005 | Rural residents^*^ | 0/901 (0) | - | - | - |
| Santhia et al., 2009 | 2005 | Market stall operators  Residents^*^ | 0/87 (0)  0/841 (0) | - | - | - |
| Schultsz et al., 2009 | 2005 | Poultry workers  Cullers^†^ | 0/183 (0)  0/317 (0) | - | - | - |
| Apisarnthanarak et al., 2006 | 2005-06 | ICU patients with community-acquired pneumonia | 0/42 (0) | - | - | - |
| Buchy et al., 2007 | 2005-06 | Household members  Health care workers  Close contact villagers | 0/10 (0)  0/28 (0)  0/42 (0) | - | - | - |
| Liao et al., 2013 | 2005 | Household contacts  Social contacts | 2/87 (2.3)  0/332 (0) | - | 0/61 (0)  5/260 (1.9) | - |
| Ilyicheva et al., 2013 | 2005-09 | Residents  Poultry farm personnel | 8/2230 (0.4)  0/51 (0) | - | - | - |
| Ceyhan et al., 2010 | 2006 | Family contacts  Exposed HCWs  Poultry cullers^†^  Exposed population  Non-exposed population | 1/28 (3.6)  0/97 (0)  0/95 (0)  0/75 (0)  0/81 (0) | - | - | - |
| Ortiz et al., 2007 | 2006 | Poultry workers  Laboratory workers | 0/295 (0)  0/25 (0) | 97% of specimens had neutralizing antibody titers of ≥1:80 against a recently circulating influenza A(H3N2) virus. | - | - |
| Vong et al., 2009 | 2006 | Rural villagers^*^ | 7/674 (1.0) | - | - | Swim and/or bathe in ponds: 11.3 (1.25–102.18) ^c^;  Gathered poultry and placed in cages and/or poultry areas: 5.8 (0.98–34.12) ^c^ |
| Wang et al., 2006 | 2006 | Poultry purveyors | 1/110 (0.9) | - | - | - |
| Cai et al., 2009 | 2006 | Local auxiliary fire brigade, local government workers and veterinarians | 0/78 (0) | A possible cross-reaction of A(H1N1) antibodies in high concentrations with the A(H5N1) reference virus was found by MN assay. | 42/89 (47.2) |  |
| Wang et al., 2008 | 2007 | Household members  Social contacts  Health care workers | 0/9 (0)  0/5 (0)  0/77 (0) | - | - | - |
| Reed et al., 2014 | 2007 | Rural subsistence bird hunters  Non-hunting family members  Urban sport hunters  Wildlife biologists  Non-exposed population | 0/237 (0)  0/229 (0)  0/164 (0)  0/82 (0)  0/204 (0) | - | - | - |
| Cavailler et al., 2010 | 2007 | Rural residents^*^ | 18/700 (2.6) | - | - | Swam/bathed in pond: 2.52 (0.98-6.51)^b^, P=0.05 |
| Robert et al., 2010 | 2010 | Poultry farmers | 0/495 (0) | - | 11/495 (2.2) | - |
| Wang et al., 2009 | 2007-08 | Poultry retailers  Poultry wholesalers  Poultry workers in larger breeding enterprise  Poultry farmers  Goods retailer (other than poultry) in LPM  Swine workers  General population | 2/252 (0.8)  2/244 (0.8)  0/125 (0)  0/869 (0)  0/182 (0)  0/218 (0)  0/301 (0) | - | - | - |
| Wallensten et al., 2009 | 2008 | Exposed populations who were euthanizing sick birds, de-ringing and bagging carcasses | 0/11 (0) | - | - | - |
| Blair et al., 2013 and Gray et al., 2014 | 2008-10 | Rural villagers^*^ | Baseline: 0/800 (0)  1-year follow-up: 0/800 (0)  2-year follow-up: 0/784 (0) | A/Brisbane/59/2007(H1N1):  Baseline: 132/774 (17.1)  Follow-up 2: 103/784 (13.1)  A/Brisbane/10/2007(H3N2):  Baseline: 560/773 (72.5)  Follow-up 2: 326/784 (41.6)  A/Mexico/4108/2009[pandemic A(H1N1)]:  Follow-up 2: 53/784 (6.8) | 0/800 (0)  0/800 (0)  0/784 (0) | - |
| Khuntirat et al., 2011 and Krueger et al., 2013 | 2008-10 | Rural villagers^*^ | Baseline: 45/800 (5.6)  1-year follow-up: 0/768 (0)  2-year follow-up: 1/784 (0.1) | A/Brisbane/59/2007(H1N1):  Baseline: 33/793 (41.6)  A/New Caledonia/20/99(H1N1):  Baseline: 32/797 (40.2)  A/Panama/2007/99(H3N2):  Baseline: 250/799 (31.3)  Follow-up 2: 242/782 (30.9)  A/Brisbane/10/2007(H3N2):  Baseline: 539/797 (67.6) | 13/800 (1.6)  44/781 (5.6) | A/Thailand/676/2005(H5N1):  Persons aged ≥60 years-old: 31.2 (5.0-󠅈∞) ^b^, persons aged 40-59 years-old: 8.4 (1.3-354.8) ^b^, ref: persons aged 20-39 years-old  A/New Caledonia/20/99(H1N1) positive: 4.2 (1.4-12.9) ^b^  No indoor water: 3.2 (1.7-6.1) ^b^  A/Thailand/384/2006(H5N1):  Persons aged ≥60 years-old: 8.2 (1.9-75.2) ^b^, ref: persons aged 20-39 years-old  Any poultry exposure: 3.3 (1.1-13.1) ^b^  No indoor water: 3.1 (1.4-6.7) ^b^  Persons with chronic breathing problems: 4.0 (1.3-11.7) ^b^ |
| Ly et al., 2016 | 2008-10 | Military trainees^*^  Rural residents^*^ | 0/394 (0)  2009: 10/622 (1.6)  2010: 0/366 (0) | - | - | All age group:  Poultry cage or place located under or attached to the house: 6.7 (1.6-28.3) ^c^; transporting poultry to trade: 17.6 (1.6-193.7) ^c^  Age under 20 years-old:  Swimming or bathing in ponds also accessed by poultry: 4.6 (1.1-19.1) ^c^ |
| Okoye et al., 2013 | 2008-11 | Poultry exposed  Non-exposed population | Baseline: 0/316 (0)  1-year follow-up: 4/316 (1.3)  2-year follow-up: 5/316 (1.6)  Baseline: 1/54 (1.9)  1-year follow-up: 0/54 (0)  2-year follow-up: 0/54 (0) | A/Brisbane/59/2007(H1N1):  Baseline: 65/368 (17.7)  Follow-up 1: 77/370 (20.8)  Follow-up 2: 40/370 (10.8)  A/Brisbane/10/2007(H3N2):  Baseline: 113/369 (30.6)  Follow-up 1: 145/370 (39.2)  Follow-up 2: 97/370 (26.2)  A/Mexico/4108/2009(H1N1):  Baseline: 6/369 (1.6)  Follow-up 1: 18/370 (4.9)  Follow-up 2: 51/370 (13.8) | Baseline: 1/370 (0.3) | A/Brisbane/59/2007(H1N1)  Positive: unadjusted OR^b^=0.04 (0.02–0.08), ref: negative;  A/Brisbane/10/2007(H3N2)  Positive: unadjusted OR^b^=0.4 (0.2–0.9), ref: negative; |
| Cao et al., 2013 | 2008-12 | Exposed swine farm residents  Non-exposed urban residents | 10/1606 (0.6)  0/104 (0) | A(H1N1): 522/1710 (30.5)  A(H3N2): 293/1710 (17.1)  dual reactivity toward A(H5N1) and seasonal influenza: 3 samples - | - | - |
| Nasreen et al., 2013 | 2009 | LPM workers  Farm workers | 0/210 (0)  0/212 (0) | - | - | Slaughtered poultry: RR=11.0 (1.4–87.1); defeathered poultry: RR=7.2 (1.5–34.5); eviscerated poultry: RR=6.8 (1.4–33.0) |
| Zhang et al., 2011 | 2009 | Blood donors | 0/200 (0) | A(H1N1)pdm: 11% displayed activity, GMT=1.7;  A(H3N2): 86% displayed neutralizing activity, GMT=50.8 | - | - |
| Nasreen et al., 2015 | 2009-10 | LPM workers  Non-poultry workers | Baseline: 9/404 (2.2)  Follow-up visit 1: 17/278 (6.1)  Overall: 20/404 (5.0)  0/101 (0) | - | - | High risk behavior (feed poultry, clean feeding tray, clean water container, clean feces from poultry pen, do not wash hands after handling sick poultry): RR=7.6 (2.8–20.9);  medium risk behavior (slaughter poultry, defeather poultry, eviscerate poultry, collect or transport poultry feces, stuff poultry into bags): RR=5.1 (1.8–14.1) |
| Coman et al., 2013 | 2009-10 | Commercial swine workers  Traditional backyard farm workers  Non-exposed population | 0/149 (0)  0/163 (0)  0/51 (0) | A/Brisbane/59/2007(H1N1):  30/309^***^ (9.7), 9/51 (17.6)  A/New Caledonia/20/1999(H1N1):  37/311 ^***^ (11.9), 5/51 (9.8)  A/Mexico/4108/2009(H1N1):  8/300^***^ (2.6), 1/47 (2.1)  A/Brisbane/10/2007(H3N2):  50/312^***^ (16.0), 15/51 (29.4) | 118/306^***^ (37.8)  35/51 (68.6) | - |
| Yu et al., 2013 | 2009-10 | Duck keeper  Chicken keeper  Chicken butcher | 0/155 (0)  0/114 (0)  0/36 (0) | - | 0/155 (0)  0/114 (0)  0/36 (0) | - |
| Huo et al., 2012 | 2010 | Backyard poultry farmers | 8/306 (2.6) | - | - | Raising poultry number: 2.39 (1.00-5.69) ^b^ |
| Chen et al., 2011 | 2010 | Residents in rural areas | 0/1039 (0) | Positive threshold: 1:40:  A/Brisbane/59/2007 (H1N1): 14/1039  A/California/07/2009 (H1N1pdm): 32/1039  A/Perth/16/2009-like (H3N2):174/1032 | 0/1039 (0) | - |
| Pawar et al., 2014 | 2010  2012  2009 | Poultry workers  General population | 0/338 (0)  0/128 (0)  0/162 (0) | - | - | - |
| Ahad et al., 2014 | 2010-11 | Poultry farm workers | 0/354 (0) | - | 0/354 (0) | - |
| Li et al., 2013 | 2010-12 | LPM workers  Large scale poultry company workers  Poultry slaughtering and processing plants workers  Backyard poultry farmers | 13/241 (5.4)  25/537 (4.7)  0/36 (0)  17/355 (4.8) | - | - | Number of poultry bred >1,000: 3.77 (1.72, 8.73) ^b^;  Direct or close contact with poultry: 5.20 (1.53, 17.74) ^b^ |
| Gomaa et al., 2014 | 2010-12 | Occupational population  Non-exposed population | Baseline: 15/708 (2.1)  1-year follow-up: 3/682 (0.4)  2-year follow-up: 4/649 (0.6)  Baseline: 0/224 (0)  1-year follow-up: 0/139 (0)  2-year follow-up: 0/104 (0) | A/California/04/09 (H1N1):  Baseline: 185/721 (25.7), 25/229 (10.9);  Follow-up 1: 87/686 (12.7), 24/141 (17.0);  Follow-up 2: 335/651 (51.5), 58/104 (55.8)  A/Brisbane/10/07 (H3N2):  Baseline: 252/719 (35.0), 74/229 (32.3);  Follow-up 1: 42/686 (6.1), 1/141 (0.7);  Follow-up 2: 202/649 (31.1), 41/104 (39.4) | 17/749 (2.3)  14/249 (5.6) | Chronic lung problems: 12.6 (3.8-41.7)^b^, ref: no chronic lung problems; Persons aged 17-50 years-old: 5.2 (1.1-23.2) ^b^, ref: persons aged <6 years-old |
| Dung et al., 2014 | 2011 | Poultry sellers, slaughterers  Other poultry workers | 31/380 (8.2)  6/227 (2.6) | - | - | - |
| Chea et al., 2014 | 2011 | Household member  Neighbor  HCWs  Social contacts | 0/11 (0)  0/3 (0)  NA  NA | - | - | - |
| Chakraborty et al., 2017 | 2011 | Close contacts (including parents and caregivers) | 0/57 (0) | - | - | - |
| Su et al., 2013 | 2011-12 | Veterinarians | 0/406 (0) | - | - | - |
| Shi et al., 2014 | 2011-12 | Local staff of the Eastern Dongting Lake Wetland Nature Reserve and on duck farms, and individual duck breeder | 0/1050 (0) | - | - | - |
| Xiong et al., 2014 | 2012 | General population | 0/394 (0) | A/New Caledonia/20/1999 (H1N1):  326/394 (82.7)  A/California/07/2009 (H1N1):  165/394 (41.8) | - | - |
| Shimizu et al., 2016 | 2012-16 | LPM workers | 2012:  Cut-off (1:32): 30/63 (47.6)  Cut-off (1:80): 8/63 (12.7)  2014:  Cut-off (1:32): 85/101 (84.0)  Cut-off (1:80): 63/101 (62.4)  2015:  Cut-off (1:32): 59/100 (59.0)  Cut-off (1:80): 22/100 (22.0)  2016:  Cut-off (1:32): 37/142 (26.1)  Cut-off (1:80): 12/142 (8.5) | - | - | No cross-reactivity had been found between A(H5N1) and seasonal H1N1 and H3N2 using person correlation coefficient. |
| Horm et al., 2016 | 2013 | LPM workers | Baseline: 1/125 (0.8);  2^nd^ resampling mission: 0/117 (0)  3^rd^ resampling mission: 2/105 (1.9)  4^th^ resampling mission: 2/106 (1.9) | - | - | - |
| Wang et al., 2014 | 2013 | Poultry market workers  General population | 1st survey: 4/501 (0.8)  2nd survey: 3/375 (0.8)  1st survey: 0/417 (0)  2nd survey: 0/408 (0) | - | 197/485 (40.6) | - |
| Chen et al., 2015 | 2013-14 | Swine workers  Poultry farm workers  LPM workers  Veterinarians  Volunteers | 1/171 (0.6)  3/150 (2.0)  5/105 (4.8)  2/120 (1.7)  0/264 (0) | A/Guangdong/1057/2010(H1N1):  93/171 (54.4)  78/150 (52.0)  51/105 (48.6)  68/120 (56.7)  134/264 (50.8) | 0/171 (0)  0/150 (0)  0/105 (0)  0/120 (0)  0/264 (0) | LPM workers: 20.9 (1.1-399.1) ^a^, ref: volunteers;  Avian exposed: 4.5 (1.2-16.2) ^a^, ref: no exposure history;  Any animal exposure: 20.1 (1.2-333.7) ^a^, ref: no animal exposure history |
| To et al., 2015 | 2013-14 | LPM workers  Slaughterhouse workers | Baseline:  Cut-off (1:40): 0/30 (0)  Cut-off (1:160): 0/30 (0)  Follow-up visit 1:  Cut-off (1:40): 17/45 (37.8)  Cut-off (1:160): 1/45 (2.2)  Baseline:  Cut-off (1:40): 0/69 (0)  Cut-off (1:160): 0/69 (0)  Follow-up visit 1:  Cut-off (1:40): 1/27 (3.7)  Cut-off (1:160): 0/27 (0) | - | Any time since 2009: Baseline: 14/30 (46.7), 36/69 (52.2);  Follow-up visit 1: 26/45 (57.8), 19/27 (70.4) | - |
| Ma et al., 2015 | 2013-14 | Family members  HCWs  Other contacts in health care setting | 0/30 (0)  0/177 (0)  0/18 (0) | A/California/07/2009 (H1N1): 0/30 (0), 5/177 (2.8), 0/18 (0)  A/Victoria/210/2009 (H3N2): 10/30 (33.3), 88/177 (49.7), 9/18 (50.0) | - | - |
| Yang et al., 2016 | 2013-15 | Poultry workers  Swine workers  Non-exposed general population | Baseline:  A(H5N1) virus antigen 1: 0/1258 (0)  A(H5N1) virus antigen 2: 0/1258 (0)  Follow-up visit 1:  A(H5N1) virus antigen 1: 17/1056 (1.6)  A(H5N1) virus antigen 2: 2/1056 (0.2)  Follow-up visit 2:  A(H5N1) virus antigen 1: 2/1123 (0.2)  A(H5N1) virus antigen 2: 1/1123 (0.1)  Baseline:  A(H5N1) virus antigen 1: 1/1332 (0.1)  A(H5N1) virus antigen 2: 2/1332 (0.2)  Follow-up visit 1:  A(H5N1) virus antigen 1: 1/1254 (0.1)  A(H5N1) virus antigen 2: 0/1254 (0)  Follow-up visit 2:  A(H5N1) virus antigen 1: 1/998 (0.1)  A(H5N1) virus antigen 2: 2/998 (0.2)  Baseline:  A(H5N1) virus antigen 1: 0/1200 (0)  A(H5N1) virus antigen 2: 0/1200 (0)  Follow-up visit 1:  A(H5N1) virus antigen 1: 0/1188 (0)  A(H5N1) virus antigen 2: 0/1188 (0)  Follow-up visit 2:  A(H5N1) virus antigen 1: 0/1135 (0)  A(H5N1) virus antigen 2: 0/1135 (0) | - | - | A(H5N1) clade 2.3.4 (incidence rate ratio, IRR):  Poultry workers:  adjusted IRR=10.58 (95%CI: 4.43-25.30), ref: general population |
| Ma et al., 2018 | 2013-16 | Poultry workers  Swine workers  General population control | Baseline: 1/511 (0.2)  Follow-up visit 1: 0/533 (0)  Follow-up visit 2: 0/535 (0)  Follow-up visit 3: 17/491 (3.5)  Overall: 18/964 (1.9)  Baseline: 0/569 (0)  Follow-up visit 1: 0/589 (0)  Follow-up visit 2: 0/501 (0)  Follow-up visit 3: 0/367 (0)  Overall: 0/1079  Baseline: 0/915 (0)  Follow-up visit 1: 0/881 (0)  Follow-up visit 2: 0/855 (0)  Follow-up visit 3: 0/785 (0)  Overall: 0/1545 (0) | - | 10/502 (2.0)  2/527 (0.4)  9/521 (1.7)  5/482 (1.0)  16/557 (2.9)  3/587 (0.5)  2/499 (0.4)  2/367 (0.5)  14/892 (1.6)  3/878 (0.3)  1/848 (0.1)  1/783 (0.1) | Pigeon exposure history: 3.13 (1.2-8.0) ^b^; female: 5.5 (2.4-12.6) ^b^, ref: male |
| Ly et al., 2017 | 2014 | Rural residents^*^ | 1/238 (0.4) in Kratie  1/643 (0.2) in Kompong Cham | - | - | - |
| Quan et al, 2019 | 2014-16 | Poultry workers  General population | Baseline:  A(H5N1) virus antigen 1: 6/700 (0.9)  A(H5N1) virus antigen 2: 22/700 (3.1)  Follow-up visit 1:  A(H5N1) virus antigen 1: 6/506 (1.2)  A(H5N1) virus antigen 2: 17/506 (3.4)  Follow-up visit 2:  A(H5N1) virus antigen 1: 10/481 (2.1)  A(H5N1) virus antigen 2: 2/481 (0.4)  Follow-up visit 3:  A(H5N1) virus antigen 1: 6/437 (1.4)  A(H5N1) virus antigen 2: 3/437 (0.7)  Baseline:  A(H5N1) virus antigen 1: 3/216 (1.4)  A(H5N1) virus antigen 2: 3/216 (1.4) | A/California/04/2009 (H1N1): 348/2124 (16.4), 47/216 (21.8)  A/Beijing/CAS0001/2007 (H3N2): 772/2124 (36.6), 75/216 (34.7) | 23/700 (3.3)  8/506 (1.6)  20/481 (4.2)  8/437 (1.8)  - | - |
| Sirawan et al., 2020 | 2017 | Poultry workers | 0/69 (0) | - | - | - |

Abbreviations: LPM, live poultry market; unk, unknown; NA, not available; ref, reference group.

^a^ Unmatched odds ratio

^b^ Binary logistic regression (adjusted OR)

^c^ Conditional logistic regression

^d^ Matched odds ratio

^†^Persons who involved in poultry culling operations when A(H5N1) outbreaks in poultry occurred.

^*^Residents living in the region where A(H5N1) outbreaks in humans, poultry, or both occurred during the study periods.

^**^Epidemiologists, public health officials, and media reporters.

^***^Swine workers include commercial swine workers and traditional backyard farm workers.

Table S5. Summary of studies reporting seroconversion rate and seroincidence of human infections with highly pathogenic avian influenza A(H5N1) virus included in systematic review

| **Reference** | **Study period** | **Study population** | **No. of participants** | **No. of participants provided paired sera** | **No. of seroconversions** | **Participation during** **the follow-up visits** | **Seroconversion rate (%)** | **Seroincidence rate during the follow-up visits** |
| --- | --- | --- | --- | --- | --- | --- | --- | --- |
| Katz et al., 1999 | 1997 | Household contacts  Tour group members | 51  26 | unk  26 | 2  0 | unk  unk | unk  0/26 (0) | unk  0/26 (0) |
| Bridges et al., 2000 | 1997 | Exposed HCWs | 217 | 194 | 2 | unk | 2/194 (1.0) | 2/194 (1.0) |
| Bridges et al., 2002 | 1997-98 | GWs | 293 | 229 | 1 | unk | 1/229 (0.4) | 0.22 cases/100 person-weeks |
| Kwon et al., 2012 | 2003 | All bird workers | 2512 | 936 | 3 | unk | 3/936 (0.3) | 3/936 (0.3) |
| Schultsz et al., 2005 | 2004 | HCWs | 62 | 46 | 0 | unk | 0/46 (0) | 0/46 (0) |
| Apisarnthanarak et al., 2004 | 2004 | Exposed HCWs  Non-exposed HCWs | 25  24 | 25  24 | 0  0 | unk  unk | 0/25 (0)  0/24 (0) | 0/25 (0)  0/24 (0) |
| Apisarnthanarak et al., 2006 | 2005 | ICU patients with community-acquired pneumonia | 115 | 42 | 0 | unk | 0/42 (0) | 0/42 (0) |
| Liao et al., 2013 | 2005 | Household contacts  Social contacts | 87  332 | 35  196 | 0  0 | unk  unk | 0/35 (0)  0/396 (0) | 0/35 (0)  0/396 (0) |
| Ceyhan et al., 2010 | 2006 | Exposed HCWs | 97 | 97 | 0 | unk | 0/97 (0) | 0/97 (0) |
| Wang et al., 2008 | 2007 | Household members  Social contacts  Health care workers  overall | 9  5  77  91 | unk  unk  unk  30 | 0  0  0  0 | unk  unk  unk  unk | unk  unk  unk  0/30 (0) | unk  unk  unk  0/30 (0) |
| Blair et al., 2013 and Gray et al., 2014 | 2008-10 | Rural villagers^*^ | Baseline: 800  1-year follow-up: 800  2-year follow-up: 784 | 800  800  708 | unk  unk  unk | unk  unk  unk | unk  unk  unk | unk  unk  unk |
| Khuntirat et al., 2011 and Krueger et al., 2013 | 2008-10 | Rural villagers^*^ | Baseline: 800;  1-year follow-up: 768  2-year follow-up: 784 | -  unk  unk | 1 | unk  unk  unk | -  unk  unk | -  unk  unk |
| Okoye et al., 2013 | 2008-11 | Poultry exposed  Non-exposed population | Baseline: 316  1-year follow-up: 316  2-year follow-up: 316  Baseline: 54  1-year follow-up: 54  2-year follow-up: 54 | 316  316  54  54 | Cut-off (1:10): 4  Cut-off (1:10): 5  Cut-off (1:10): 0  Cut-off (1:10): 0 | 316 person-years  316 person-years  54 person-years  54 person-years | 4/316 (1.3)  5/316 (1.6)  0/54 (0)  0/54 (0) | 1.3 cases/100 person-years  1.6 cases/100 person-years  0 cases/100 person-years  0 cases/100 person-years |
| Nasreen et al., 2015 | 2009-10 | LPM workers | Overall: 404 | 278 | 6 | 30043 person-days (82 person-years) | 6/278 (2.2) | 7.3 cases/100 person-years |
| Gomaa et al., 2014 | 2010-12 | Occupational population  Non-exposed population | Baseline: 750  1-year follow-up: 682  2-year follow-up: 649  Baseline: 250  1-year follow-up: 139  2-year follow-up: 104 | -  682  649  -  139  104 | unk  unk  unk  unk | -  682 person-years  649 person-years  -  139 person-years  104 person-years | -  unk  unk  -  unk  unk | -  unk  unk  -  unk  unk |
| Chakraborty et al., 2017 | 2011 | Close contacts (including parents and caregivers) | 57 | unk | 0 | unk | unk | unk |
| Shimizu et al., 2016 | 2012-16 | LPM workers | 63 in 2012  101 in 2014  100 in 2015  142 in 2016 | 25  36  58 | 11  1  3 | 550 person-months  432 person-months  1450 person-months | 11/25 (44.0)  1/36 (2.8)  3/58 (5.2) | 2.0 cases/100 person-months  0.2 cases/100 person-months  0.2 cases/100 person-months |
| Horm et al., 2016 | 2013 | LPM workers | Baseline: 125;  2^nd^ resampling mission: 117  3^rd^ resampling mission: 105  4^th^ resampling mission: 106 | -  117  105  106 | -  0  2  2 | -  234 person-months  315 person-months  530 person-months | -  0/117 (0)  2/105 (1.9)  2/106 (1.9) | -  0 cases/100 person-months  0.6 cases/100 person-months  0.4 cases/100 person-months |
| Wang et al., 2014 | 2013 | LPM workers | 1st survey: 501  2nd survey: 375 | 96 | unk | 768 person-months | unk | unk |
| To et al., 2015 | 2013-14 | LPM workers  Slaughterhouse workers | Baseline: 30  Follow-up visit 1: 45  Baseline: 69  Follow-up visit 1: 27 | 10  12 | 6  1 | unk  unk | 6/10 (60.0)  1/12 (8.3) | 6/10 (60.0)  1/12 (8.3) |
| Yang et al., 2016 | 2013-15 | Poultry workers  Swine workers  Non-exposed general population | Overall: unk  Overall: unk  Overall: unk | unk  unk  unk | clade2.3.4: 38  clade 2.3.2.1: 3  clade2.3.4: 4  clade 2.3.2.1: 2  clade2.3.4: 6  clade 2.3.2.1: 2 | 10099 person-months  10099 person-months  11126 person-months  11126 person-months  15734 person-months  15734 person-months | unk  unk  unk  unk  unk  unk | 0.4 cases/100 person-months  0.03 cases/100 person-months  0.04 cases/100 person-months  0.02 cases/100 person-months  0.4 cases/100 person-months  0.01 cases/100 person-months |
| Ma et al., 2018 | 2013-16 | Poultry workers  Swine workers  General population control | Overall: 964  Overall: 1079  Overall: 1545 | 468  514  1030 | 7  0  0 | 1569 person-years  1558 person-years  2586 person-years | 7/468 (1.5)  0/514 (0)  0/1030 (0) | 4.5 cases/100 person-years  0 cases/100 person-years  0 cases/100 person-years |
| Ly et al., 2017 | 2014 | Rural residents^*^ | 695 in Kratie  921 in Kompong Cham | 238  643 | unk  unk | unk  unk | unk  unk | unk  unk |
| Quan et al, 2019 | 2014-16 | Poultry workers | Overall: 1407 | 652 | 2.3.2.1 c: 1  2.3.4.4: 5  Overall: 6 | unk  unk  unk | 1/652 (0.2)  5/652 (0.8)  6/652 (0.9) | unk  unk  unk |

Abbreviations: LPM, live poultry market; HCW, health care worker; GW, government worker; unk, unknown; NA, not available; ref, reference group.

Table S6. Definition of subjects included in meta-analysis

| **Type of exposure** | **Population** | **Definition** |
| --- | --- | --- |
| Only exposed to poultry or wild bird | Poultry workers^*^ | Occupational populations expected to have prolonged unprotected poultry exposures or high-level exposures to poultry (e.g. poultry breeders, transporters and drivers, sellers, slaughterers veterinarians, feather collectors, cleaners, or market managers) in backyard farms, commercial farms or live poultry markets (LPMs). |
|  | Poultry cullers | Occupational populations involved in culling poultry or wild birds, euthanizing sick poultry or birds, cleaning and bagging carcasses when A(H5N1) virus infections confirmed in poultry flocks or wild birds. These people occasionally had unprotected high-level exposures to domestic poultry or wild birds. |
|  | Other occupationally-exposed populations | Occupational population with occasional exposure to confirmed or suspected A(H5N1) virus in culture or in poultry specimens, or during an outbreak investigation, e.g. laboratory worker, epidemiologists, public health officials, and journalists. |
|  | Poultry-exposed residents | Residents who were living in a region where A(H5N1) outbreaks in poultry occurred, or had known exposure to poultry, wild birds or live poultry markets (LPMs). |
| Only exposed to human A(H5N1) case | Household contacts | A person or a group of people who resided in the same household as a confirmed A(H5N1) case during the case’s infectious period, and without any poultry/wild birds/LPM exposure history, i.e. household members and family contacts. |
|  | Social contacts | Persons (e.g. friends, neighbors, tour group members, visitors, colleagues and co-workers) not living in the same household who had close contact with a confirmed A(H5N1) case during the case’s infectious period, and had never been exposed to poultry/wild birds/LPMs. |
|  | Exposed healthcare workers | Occupational population who provided routine medical care to confirmed or probable A(H5N1) cases in a hospital setting, without any poultry/wild birds/LPMs exposure history. |
|  | Other close contacts | Persons living/working (being at) within a 1-3 km radius from where A(H5N1) virus infections occurred in humans. |
| Exposure to both human cases and poultry/wild bird | Confirmed mixed exposure population | A person or a group of people who were close contacts of A(H5N1) cases and had known exposure to poultry/wild birds. |
|  | Suspected mixed exposure population | A person or a group of people who were living/working within a 1-3 km radius from where A(H5N1) cases and poultry outbreaks occurred or were living in a same area as an A(H5N1) case and with known contact with diseased poultry/wild birds. |
| Neither exposed to a human case nor poultry/wild bird | General population | Residents who did not have known exposure to a confirmed A(H5N1) case, or to poultry, wild bird or LPMs. |

*A swine worker with high-level poultry exposure was included in this group.

Table S7. Scoring system used for evaluation of published reports describing seroevidence of human infection with highly pathogenic avian influenza A(H5N1) virus

| Parameter | Maximum score | Individual score | | | |
| --- | --- | --- | --- | --- | --- |
|  |  | 0 | 1 | 2 | 3 |
| Control group | 6 | No | Unmatched | Age-matched  Sex-matched  Area-matched | NA |
| Repeated sampling | 2 | No | Yes | Yes | NA |
| Correction for age or reporting of study participants’ age groups | 1 | No | Yes | NA | NA |
| Human influenza vaccination status reported | 1 | No | Yes | NA | NA |
| Testing included human influenza type(s) and influenza A virus subtypes | 1 | No | Yes | NA | NA |
| Other evidence | 3 | No | Serological evidence in  animals to which humans were exposed | Virological evidence of A(H5N1) virus infection in animals to which humans were exposed | Virological evidence of A(H5N1) virus infection in human study participants |
| Laboratory method | 5 | NA | NA | NA | NA |
| Total | 18 | NA | NA | NA | NA |

Table S8. Scores for antibody detection assays assigned to published studies on human infection with highly pathogenic avian influenza A(H5N1) virus

| **Confirmation method** | **Screening method** | | | | | |
| --- | --- | --- | --- | --- | --- | --- |
|  | **MN** | **Plaque neutralization assay** | **HAI**  **(horse RBCs)** | **ELISA** | **HAI**  **(other RBCs)** | **None** |
| MN^a^ | NA | 5 | 5 | 5 | 4 | 3 |
| NT | 5 | NA | 5 | 4 | 3 | 3 |
| HAI (horse RBCs) | 5 | NA | NA | 4 | NA | 2 |
| ELISA | 5 | NA | 4 | NA | 3 | 2 |
| Western blot | 5 | NA | 4 | 4 | 3 | 0 |
| HAI (other RBCs) | 4 | NA | NA | 3 | NA | 1 |
| None | 3 | 3 | 2 | 2 | 1 | NA |

Note: MN, microneutralization assay; NT, neutralization assay; HAI, hemagglutination inhibition assay; RBCs, red blood cells; ELISA, enzyme linked immunosorbent assay.

^a^ Including ELISA-based, HA/cytopathic effect (CPE)-based and H5 hemagglutinin pseudotyped particles (H5pp) based microneutralization assay

Table S9. Quality assessment of 66 serological studies describing subclinical and clinically mild human infection with highly pathogenic avian influenza A(H5N1) virus, 1997-2019

| Reference | Study population | Ascertainment of participants’ any febrile and respiratory illness (yes/no/not reported^*^) | Control group | Repeated sampling | Correction for age or reporting of study participants’ age groups | Human influenza vaccination status reported | Testing included human influenza type(s) | Other evidence | | | Confirmation method | Total | Grade |
| --- | --- | --- | --- | --- | --- | --- | --- | --- | --- | --- | --- | --- | --- |
|  |  |  |  |  |  |  |  | Serological evidence in animals to which humans were exposed | Virological evidence in animals to which humans were exposed | Virological evidence in human study participants |  |  |  |
| Katz et al., 1999 | Confirmed mixed exposure population,  Suspected exposure population | Confirmed mixed exposure population: yes;  Suspected exposure population: no | 0 | 2 | 0 | 0 | 0 | 0 | 0 | 0 | 5 | 7 | C |
| Bridges et al., 2000 | Confirmed mixed exposure population,  Suspected exposure population | Yes | 0 | 2 | 1 | 0 | 0 | 0 | 0 | 0 | 5 | 8 | C |
| Bridges et al., 2002 | Poultry workers, poultry cullers | Poultry workers: no;  poultry culler: yes | 0 | 2 | 1 | 0 | 0 | 0 | 0 | 0 | 5 | 8 | C |
| Uyeki et al., 2012 | Poultry workers, general population | No | 2 | 0 | 0 | 0 | 0 | 0 | 0 | 0 | 5 | 7 | C |
| Kwon et al., 2012 | Poultry workers,  poultry cullers, other occupationally-exposed population | Poultry workers: not reported;  poultry cullers: yes;  other occupationally-exposed population: not reported | 0 | 2 | 1 | 0 | 0 | 0 | 0 | 0 | 5 | 8 | C |
| Liem et al., 2005 | Confirmed mixed exposure population | Not reported | 0 | 0 | 1 | 1 | 1 | 0 | 0 | 0 | 5 | 8 | C |
| Schultsz et al., 2005 | Confirmed mixed exposure population | Yes | 0 | 2 | 0 | 0 | 1 | 0 | 0 | 3 | 5 | 11 | B |
| Lu et al., 2008 | Poultry workers,  poultry-exposed residents | Poultry workers: Yes;  poultry-exposed residents: no | 0 | 0 | 0 | 0 | 1 | 0 | 0 | 0 | 4 | 5 | C |
| Hinjoy et al., 2008 | Poultry workers | No | 0 | 0 | 1 | 0 | 0 | 0 | 0 | 0 | 5 | 6 | C |
| Apisarnthanarak et al., 2004 | Exposed healthcare workers, other close contacts | Yes | 2 | 2 | 0 | 0 | 0 | 0 | 0 | 0 | 5 | 9 | C |
| Khuntirat et al., 2015 | Poultry-exposed residents | Not reported | 0 | 2 | 0 | 0 | 0 | 0 | 0 | 0 | 3 | 5 | C |
| Vong et al., 2006 | Suspected exposure population | Yes | 0 | 0 | 0 | 0 | 0 | 1 | 2 | 0 | 5 | 7 | C |
| Dejpichai et al., 2009 | Confirmed mixed exposure population | Not reported | 0 | 0 | 1 | 0 | 0 | 0 | 0 | 0 | 5 | 6 | D |
| Santhia et al., 2009 | Poultry workers, poultry-exposed residents | No | 0 | 0 | 0 | 0 | 0 | 1 | 2 | 0 | 3 | 5 | C |
| Schultsz et al., 2009 | Poultry workers, poultry cullers | Yes | 0 | 0 | 0 | 0 | 1 | 0 | 0 | 0 | 5 | 6 | C |
| Apisarnthanarak et al., 2006 | General population | Not reported | 0 | 2 | 0 | 0 | 0 | 0 | 0 | 3 | 5 | 10 | C |
| Buchy et al., 2007 | Household contacts, exposed healthcare workers, social contacts | No | 0 | 0 | 0 | 0 | 0 | 0 | 0 | 3 | 5 | 8 | C |
| Liao et al., 2013 | Confirmed mixed exposure population | Yes | 0 | 2 | 0 | 1 | 1 | 0 | 2 | 0 | 5 | 11 | C |
| Ilyicheva et al., 2013 | Poultry-exposed residents | Yes | 0 | 0 | 0 | 0 | 0 | 0 | 0 | 0 | 5 | 5 | C |
| Ceyhan et al., 2010 | Poultry culler, exposed healthcare workers, confirmed mixed exposure population, poultry-exposed residents, general population | Yes | 2 | 2 | 0 | 0 | 0 | 0 | 0 | 0 | 5 | 9 | C |
| Ortiz et al., 2007 | Poultry workers, other occupationally-exposed population | Not reported | 0 | 0 | 0 | 0 | 1 | 0 | 2 | 0 | 5 | 8 | C |
| Vong et al., 2009 | Confirmed mixed exposure population | Yes | 0 | 0 | 1 | 0 | 0 | 1 | 2 | 0 | 5 | 8 | C |
| Wang et al., 2006 | Poultry workers | Yes | 0 | 0 | 0 | 0 | 0 | 0 | 2 | 0 | 5 | 7 | C |
| Cai et al., 2009 | Poultry workers | Yes | 0 | 0 | 0 | 1 | 1 | 0 | 0 | 0 | 5 | 7 | C |
| Wang et al., 2008 | Confirmed mixed exposure population | Yes | 0 | 2 | 0 | 0 | 0 | 0 | 0 | 3 | 5 | 10 | B |
| Reed et al., 2014 | Poultry workers, other occupationally-exposed population, general population | No | 2 | 0 | 1 | 0 | 0 | 0 | 0 | 0 | 5 | 8 | C |
| Cavailler et al., 2010 | Confirmed mixed exposure population | Yes | 0 | 0 | 0 | 0 | 0 | 0 | 0 | 0 | 5 | 5 | C |
| Robert et al., 2010 | Poultry workers | Not reported | 0 | 0 | 1 | 1 | 0 | 0 | 0 | 0 | 3 | 5 | C |
| Wang et al., 2009 | Poultry workers, poultry-exposed residents, general population | No | 2 | 0 | 0 | 0 | 0 | 0 | 0 | 0 | 4 | 6 | C |
| Wallensten et al., 2009 | Poultry workers | Yes | 0 | 0 | 0 | 0 | 0 | 0 | 0 | 0 | 5 | 5 | C |
| Blair et al., 2013 | Suspected mixed exposure population | Not reported | 0 | 0 | 1 | 1 | 1 | 0 | 0 | 0 | 3 | 6 | C |
| Gray et al., 2014 | Confirmed mixed exposure population | Not reported | 0 | 2 | 1 | 1 | 1 | 0 | 0 | 3 | 3 | 11 | B |
| Khuntirat et al., 2011 | Poultry-exposed residents | Not reported | 0 | 0 | 1 | 1 | 1 | 0 | 0 | 0 | 3 | 6 | C |
| Krueger et al., 2013 | Poultry-exposed residents | Not reported | 0 | 2 | 0 | 1 | 1 | 0 | 0 | 0 | 3 | 7 | C |
| Ly et al., 2016 | Poultry-exposed residents, suspected mixed exposure population | No | 0 | 0 | 1 | 0 | 0 | 1 | 2 | 0 | 5 | 8 | C |
| Okoye et al., 2013 | Poultry workers, general population | Not reported | 1 | 2 | 1 | 1 | 1 | 0 | 0 | 0 | 3 | 9 | C |
| Okoye et al., 2014 | Poultry workers, general population | Not reported | 1 | 2 | 0 | 0 | 1 | 0 | 0 | 0 | 3 | 7 | C |
| Cao et al., 2013 | Poultry workers, general population | No | 1 | 0 | 0 | 0 | 1 | 1 | 0 | 0 | 4 | 7 | C |
| Nasreen et al., 2013 | Poultry workers | Not reported | 0 | 0 | 0 | 0 | 0 | 0 | 0 | 0 | 5 | 5 | C |
| Zhang et al., 2011 | General population | No | 0 | 0 | 0 | 0 | 1 | 0 | 0 | 0 | 5 | 6 | C |
| Nasreen et al., 2015 | Poultry workers, general population | Poultry worker: not reported; general population: no | 2 | 2 | 0 | 0 | 1 | 0 | 0 | 0 | 5 | 10 | B |
| Coman et al., 2013 | Poultry workers, general population | Not reported | 6 | 0 | 1 | 1 | 1 | 0 | 0 | 0 | 3 | 12 | B |
| Yu et al., 2013 | Poultry workers | No | 0 | 0 | 1 | 1 | 0 | 0 | 0 | 0 | 3 | 5 | C |
| Huo et al., 2012 | Poultry workers | Not reported | 0 | 0 | 0 | 0 | 0 | 0 | 0 | 0 | 2 | 2 | D |
| Chen et al., 2011 | General population | No | 0 | 2 | 0 | 1 | 1 | 0 | 0 | 0 | 1 | 5 | C |
| Pawar et al., 2014 | Poultry cullers, general population | No | 1 | 0 | 0 | 0 | 0 | 0 | 0 | 0 | 4 | 5 | C |
| Ahad et al., 2014 | Poultry workers | No | 0 | 0 | 0 | 1 | 0 | 0 | 0 | 0 | 2 | 3 | D |
| Li et al., 2013 | Poultry workers | Yes | 0 | 0 | 1 | 0 | 0 | 1 | 2 | 0 | 5 | 8 | C |
| Gomaa et al., 2014 | Poultry workers, general population | Not reported | 1 | 2 | 1 | 1 | 1 | 0 | 0 | 0 | 5 | 11 | B |
| Dung et al., 2014 | Poultry workers | No | 0 | 0 | 1 | 0 | 0 | 0 | 0 | 3 | 5 | 9 | C |
| Chea et al., 2014 | Confirmed mixed exposure population | Yes | 0 | 2 | 0 | 0 | 0 | 0 | 0 | 3 | 5 | 10 | B |
| Chakraborty et al., 2017 | Confirmed mixed exposure population | Yes | 0 | 2 | 0 | 0 | 0 | 0 | 2 | 3 | 5 | 10 | B |
| Su et al., 2013 | Poultry workers | No | 0 | 0 | 0 | 0 | 0 | 0 | 0 | 0 | 5 | 5 | C |
| Shi et al., 2014 | Poultry workers | No | 0 | 0 | 1 | 0 | 0 | 0 | 2 | 0 | 5 | 8 | C |
| Xiong et al., 2014 | General population | No | 0 | 0 | 1 | 0 | 1 | 0 | 0 | 0 | 1 | 3 | D |
| Shimizu et al., 2016 | Poultry workers | Not reported | 0 | 2 | 0 | 0 | 1 | 0 | 2 | 3 | 5 | 11 | B |
| Horm et al., 2016 | Poultry workers | Yes | 0 | 2 | 0 | 0 | 0 | 0 | 2 | 0 | 4 | 8 | C |
| Wang et al., 2014 | Poultry workers, general population | Not reported | 2 | 2 | 1 | 1 | 1 | 0 | 0 | 0 | 2 | 9 | C |
| Chen et al., 2015 | Poultry workers, general population | No | 2 | 0 | 1 | 1 | 1 | 0 | 0 | 0 | 2 | 7 | C |
| To et al., 2015 | Poultry workers | No | 0 | 2 | 0 | 1 | 1 | 0 | 0 | 0 | 1 | 5 | C |
| Ma et al., 2015 | Household contacts, exposed healthcare workers, social contacts | Yes | 0 | 0 | 0 | 0 | 1 | 0 | 0 | 0 | 2 | 3 | D |
| Yang et al., 2016 | Poultry workers, general population | No | 2 | 2 | 1 | 0 | 0 | 0 | 0 | 0 | 2 | 7 | C |
| Ma et al., 2018 | Poultry workers, general population | Not reported | 1 | 2 | 0 | 1 | 0 | 0 | 2 | 0 | 4 | 10 | B |
| Ly et al., 2017 | Suspected mixed exposure population, confirmed exposure population | Yes | 0 | 2 | 0 | 0 | 0 | 0 | 0 | 0 | 4 | 6 | C |
| Quan et al, 2019 | Poultry workers, general population | Not reported | 1 | 2 | 1 | 1 | 1 | 0 | 2 | 0 | 4 | 12 | B |
| Sirawan et al., 2020 | Poultry workers | No | 0 | 0 | 0 | 0 | 0 | 0 | 0 | 0 | 3 | 3 | D |

*refers to the study that ascertained participants’ any febrile and respiratory illness but not reported the number of asymptomatic or symptomatic infections at a particular antibody titer threshold.

## Table S10. Apparent and estimated seroprevalence of antibodies to highly pathogenic avian influenza A(H5N1) virus by type of exposure, using three antibody titer thresholds (World Health Organization recommended, modified World Health Organization recommended, and non-standardized)

| **Study population** | **WHO recommended seropositive definition (%)** | | | | | | **Modified WHO seropositive definition (%)** | | | | | | **Non-standardized seropositive definition (%)** | | | | | |
| --- | --- | --- | --- | --- | --- | --- | --- | --- | --- | --- | --- | --- | --- | --- | --- | --- | --- | --- |
|  | **No. of studies** | **Total no. of positive** | **Total no. of participants** | **Apparent seroprevalence (median, range)** | **Estimated seroprevalence (95% confidence interval)** | **I^2^ (P)** | **No. of studies** | **Total no. of positive** | **Total no. of participants** | **Apparent seroprevalence (median, range)** | **Estimated seroprevalence (95% confidence interval)** | **I^2^ (P)** | **No. of studies** | **Total no. of positive** | **Total no. of participants** | **Apparent seroprevalence (median, range)** | **Estimated seroprevalence (95% confidence interval)** | **I^2^ (P)** |
| **All infections** | | | | | | | | | | | | | | | | | | |
| Poultry workers | 14 | 92 | 8537 | 0.0 [0.0-5.3] | 0.2 [0.0-0.5] | **86.3 (<0.001)** | 14 | 105 | 8537 | 0.0 [0.0-5.3] | 0.4 [0.1-0.6] | **87.9 (<0.001)** | 36 | 253 | 17601 | 0.0 [0.0-12.7] | 0.5 [0.3-0.7] | **86.3 (<0.001)** |
| Poultry cullers | 4 | 18 | 2032 | 0.3 [0.0-3.1] | 0.6 [0.0-1.4] | **75.2 (0.007)** | 4 | 18 | 2032 | 0.3 [0.0-3.1] | 0.6 [0.0-1.4] | **75.2 (0.007)** | 5 | 18 | 2498 | 0.0 [0.0-3.1] | 0.4 [0.0-0.9] | **73.8 (0.004)** |
| Other occupationally-exposed populations | 3 | 0 | 177 | 0.0 [0.0-0.0] | 0.0 [0.0-1.2] | 0 (1.000) | 3 | 0 | 177 | 0.0 [0.0-0.0] | 0.0 [0.0-1.2] | 0 (1.000) | 3 | 0 | 177 | 0.0 [0.0-0.0] | 0.0 [0.0-1.2] | 0 (1.000) |
| Poultry-exposed residents | 5 | 8 | 3937 | 0.0 [0.0-0.4] | 0.1 [0.0-0.3] | 36.3 (0.179) | 5 | 8 | 3937 | 0.0 [0.0-0.4] | 0.1 [0.0-0.3] | 36.3 (0.179) | 9 | 53 | 6047 | 0.0 [0.0-5.6] | 0.2 [0.0-0.5] | **85.1 (<0.001)** |
| Household contacts | 1 | 0 | 10 | 0.0 [0.0-0.0] | 0.0 [0.0-12.3] | - | 1 | 0 | 10 | 0.0 [0.0-0.0] | 0.0 [0.0-12.3] | - | 2 | 0 | 40 | 0.0 [0.0-0.0] | 0.0 [0.0-4.2] | 0 (1.000) |
| Social contacts | 1 | 0 | 42 | 0.0 [0.0-0.0] | 0.0 [0.0-3.2] | **-** | 1 | 0 | 42 | 0.0 [0.0-0.0] | 0.0 [0.0-3.2] | **-** | 2 | 0 | 60 | 0.0 [0.0-0.0] | 0.0 [0.0-2.9] | 0 (1.000) |
| Exposed health care workers | 3 | 0 | 150 | 0.0 [0.0-0.0] | 0.0 [0.0-1.3] | 0 (1.000) | 3 | 0 | 150 | 0.0 [0.0-0.0] | 0.0 [0.0-1.3] | 0 (1.000) | 4 | 0 | 327 | 0.0 [0.0-0.0] | 0.0 [0.0-0.7] | 0 (1.000) |
| Other close contacts | 1 | 0 | 24 | 0.0 [0.0-0.0] | 0.0 [0.0-5.5] | - | 1 | 0 | 24 | 0.0 [0.0-0.0] | 0.0 [0.0-5.5] | - | 1 | 0 | 24 | 0.0 [0.0-0.0] | 0.0 [0.0-5.5] | - |
| Confirmed mixed exposure population | 7 | 40 | 1813 | 1.0 [0.0-7.0] | 1.8 [0.5-3.1] | **62.1 (0.015)** | 8 | 41 | 2232 | 0.6 [0.0-7.0] | 1.4 [0.4-2.5] | **73.7 (<0.001)** | 13 | 44 | 3579 | 0.4 [0.0-7.0] | 0.8 [0.2-1.4] | **71.7 (<0.001)** |
| Suspected exposure population | 5 | 12 | 1672 | 0.0 [0.0-1.6] | 0.4 [0.0-0.9] | **62.7 (0.030)** | 5 | 12 | 1672 | 0.0 [0.0-1.6] | 0.4 [0.0-0.9] | **62.7 (0.030)** | 7 | 13 | 3115 | 0.0 [0.0-1.6] | 0.2 [0.0-0.4] | 50.4 (0.060) |
| General population | 6 | 0 | 926 | 0.0 [0.0-0.0] | 0.0 [0.0-0.3] | 0 (1.000) | 6 | 0 | 926 | 0.0 [0.0-0.0] | 0.0 [0.0-0.3] | 0 (1.000) | 18 | 3 | 6369 | 0.0 [0.0-1.9] | 0.0 [0.0-0.1] | 0 (1.000) |
| **Asymptomatic infections** | | | | | | | | | | | | | | | | | | |
| Poultry workers | 4 | 1 | 819 | 0.0 [0.0-0.9] | 0.0 [0.0-0.4] | 0 (0.807) | 4 | 1 | 819 | 0.0 [0.0-0.9] | 0.0 [0.0-0.4] | 0 (0.807) | 9 | 3 | 2713 | 0.0 [0.0-0.9] | 0.0 [0.0-0.1] | 0 (0.943) |
| Poultry cullers | 4 | 12 | 2032 | 0.3 [0.0-1.0] | 0.4 [0.0-0.9] | 51.1 (0.105) | 4 | 12 | 2032 | 0.3 [0.0-1.0] | 0.4 [0.0-0.9] | 51.1 (0.105) | 4 | 12 | 2032 | 0.3 [0.0-1.0] | 0.4 [0.0-0.9] | 51.1  (0.105) |
| Other occupationally-exposed populations | 1 | 0 | 25 | 0.0 [0.0-0.0] | 0.0 [0.0-5.3] | - | 1 | 0 | 25 | 0.0 [0.0-0.0] | 0.0 [0.0-5.3] | - | 1 | 0 | 25 | 0.0 [0.0-0.0] | 0.0 [0.0-5.3] | - |
| Poultry-exposed residents | 2 | 8 | 2356 | 0.2 [0.0-0.4] | 0.3 [0.1-0.6] | 0 (0.708) | 2 | 8 | 2356 | 0.2 [0.0-0.4] | 0.3 [0.1-0.6] | 0 (0.708) | 2 | 8 | 2356 | 0.2 [0.0-0.4] | 0.3 [0.1-0.6] | 0 (0.708) |
| Household contacts | - | - | - | - | - | - | - | - | - | - | - | - | 1 | 0 | 30 | 0.0 [0.0-0.0] | 0.0 [0.0-4.4] | - |
| Social contacts | - | - | - | - | - | - | - | - | - | - | - | - | 1 | 0 | 18 | 0.0 [0.0-0.0] | 0.0 [0.0-7.2] | - |
| Exposed health care workers | 2 | 0 | 122 | 0.0 [0.0-0.0] | 0.0 [0.0-1.4] | 0 (1.000) | 2 | 0 | 122 | 0.0 [0.0-0.0] | 0.0 [0.0-1.4] | 0 (1.000) | 3 | 0 | 299 | 0.0 [0.0-0.0] | 0.0 [0.0-0.7] | 0 (1.000) |
| Other close contacts | 1 | 0 | 24 | 0.0 [0.0-0.0] | 0.0 [0.0-5.5] | - | 1 | 0 | 24 | 0.0 [0.0-0.0] | 0.0 [0.0-5.5] | - | 1 | 0 | 24 | 0.0 [0.0-0.0] | 0.0 [0.0-5.5] | - |
| Confirmed mixed exposure population | 6 | 35 | 1730 | 1.4 [0.0-7.0] | 1.9 [0.6-3.1] | **55.5 (0.047)** | 7 | 36 | 2149 | 0.9 [0.0-7.0] | 1.4 [0.4-2.4] | **72.2 (0.001)** | 11 | 38 | 2595 | 0.5 [0.0-7.0] | 0.9 [0.2-1.6] | **61.2 (0.004)** |
| Suspected exposure population | 2 | 2 | 660 | 0.3 [0.0-0.6] | 0.2 [0.0-0.8] | 40.7 (0.194) | 2 | 2 | 660 | 0.3 [0.0-0.6] | 0.2 [0.0-0.8] | 40.7 (0.194) | 3 | 3 | 1303 | 0.2 [0.0-0.6] | 0.1 [0.0-0.4] | 0 (0.421) |
| General population | 1 | 0 | 81 | 0.0 [0.0-0.0] | 0.0 [0.0-1.7] | - | 1 | 0 | 81 | 0.0 [0.0-0.0] | 0.0 [0.0-1.7] | - | 2 | 0 | 996 | 0.0 [0.0-0.0] | 0.0 [0.0-0.2] | 0 (1.000) |
| **Symptomatic infections** | | | | | | | | | | | | | | | | | | |
| Poultry workers | 4 | 0 | 819 | 0.0 [0.0-0.0] | 0.0 [0.0-0.3] | 0 (1.000) | 4 | 0 | 819 | 0.0 [0.0-0.0] | 0.0 [0.0-0.3] | 0 (1.000) | 9 | 55 | 2713 | 0.0 [0.0-4.7] | 0.5 [0.0-1.2] | **85.8 (<0.001)** |
| Poultry cullers | 4 | 6 | 2032 | 0.0 [0.0-2.0] | 0.1 [0.0-0.5] | **50.8 (0.107)** | 4 | 6 | 2032 | 0.0 [0.0-2.0] | 0.1 [0.0-0.5] | **50.8 (0.107)** | 4 | 6 | 2032 | 0.0 [0.0-2.0] | 0.1 [0.0-0.5] | **50.8 (0.107)** |
| Other occupationally-exposed populations | 1 | 0 | 25 | 0.0 [0.0-0.0] | 0.0 [0.0-5.3] | **-** | 1 | 0 | 25 | 0.0 [0.0-0.0] | 0.0 [0.0-5.3] | **-** | 1 | 0 | 25 | 0.0 [0.0-0.0] | 0.0 [0.0-5.3] | **-** |
| Poultry-exposed residents | 2 | 0 | 2356 | 0.0 [0.0-0.0] | 0.0 [0.0-0.1] | 0 (1.000) | 2 | 0 | 2356 | 0.0 [0.0-0.0] | 0.0 [0.0-0.1] | 0 (1.000) | 2 | 0 | 2356 | 0.0 [0.0-0.0] | 0.0 [0.0-0.1] | 0 (1.000) |
| Household contacts | - | - | - | - | - | - | - | - | - | - | - | - | 1 | 0 | 30 | 0.0 [0.0-0.0] | 0.0 [0.0-4.4] | - |
| Social contacts | - | - | - | - | - | - | - | - | - | - | - | - | 1 | 0 | 18 | 0.0 [0.0-0.0] | 0.0 [0.0-7.2] | - |
| Exposed health care workers | 2 | 0 | 122 | 0.0 [0.0-0.0] | 0.0 [0.0-1.4] | 0 (1.000) | 2 | 0 | 122 | 0.0 [0.0-0.0] | 0.0 [0.0-1.4] | 0 (1.000) | 3 | 0 | 299 | 0.0 [0.0-0.0] | 0.0 [0.0-0.7] | 0 (1.000) |
| Other close contacts | 1 | 0 | 24 | 0.0 [0.0-0.0] | 0.0 [0.0-5.5] | - | 1 | 0 | 24 | 0.0 [0.0-0.0] | 0.0 [0.0-5.5] | - | 1 | 0 | 24 | 0.0 [0.0-0.0] | 0.0 [0.0-5.5] | - |
| Confirmed mixed exposure population | 6 | 5 | 1730 | 0.0 [0.0-1.8] | 0.1 [0.0-0.2] | 0 (0.476) | 7 | 5 | 2149 | 0.0 [0.0-1.8] | 0.0 [0.0-0.2] | 0 (0.591) | 11 | 6 | 2595 | 0.0 [0.0-1.8] | 0.1 [0.0-0.2] | 0 (0.862) |
| Suspected mixed exposure population | 2 | 0 | 660 | 0.0 [0.0-0.0] | 0.0 [0.0-0.3] | 0 (1.000) | 2 | 0 | 660 | 0.0 [0.0-0.0] | 0.0 [0.0-0.3] | 0 (1.000) | 3 | 0 | 1303 | 0.0 [0.0-0.0] | 0.0 [0.0-0.2] | 0 (1.000) |
| General population | 1 | 0 | 81 | 0.0 [0.0-0.0] | 0.0 [0.0-1.7] | - | 1 | 0 | 81 | 0.0 [0.0-0.0] | 0.0 [0.0-1.7] | - | 2 | 0 | 996 | 0.0 [0.0-0.0] | 0.0 [0.0-0.2] | 0 (1.000) |

The WHO recommended seropositive definition refers to a neutralizing (NT) antibody titer ≥1:80 with a positive result using a 2nd confirmatory assay [i.e. hemagglutination inhibition assay (HAI) (HAI antibody titer ≥1:160), enzyme linked immunosorbent assay or western blot assay]. The modified WHO seropositive definition refers to an NT antibody titer ≥1:80 with a positive result using a 2nd confirmatory assay (i.e. HAI antibody titer ≥1:40, ELISA or western blot assay). The non-standardized seropositive definition refers to criteria used to define a seropositive result other than the WHO or modified WHO definitions.

## Table S11. Sensitivity analysis of seroprevalence of antibodies to highly pathogenic avian influenza A(H5N1) virus, considering antigenic similarity between virus strains circulating among poultry and antigens used in laboratory assays

| **Study population** | **WHO recommended seropositive definition (%)** | | | | | **Modified WHO seropositive definition (%)** | | | | | **Non-standardized seropositive definition (%)** | | | | |
| --- | --- | --- | --- | --- | --- | --- | --- | --- | --- | --- | --- | --- | --- | --- | --- |
|  | **No. of studies** | **Total no. of positive** | **Total no. of participants** | **Apparent seroprevalence (median, range)** | **Estimated seroprevalence (95% confidence interval)** | **No. of studies** | **Total no. of positive** | **Total no. of participants** | **Apparent seroprevalence (median, range)** | **Estimated seroprevalence (95% confidence interval)** | **No. of studies** | **Total no. of positive** | **Total no. of participants** | **Apparent seroprevalence (median, range)** | **Estimated seroprevalence (95% confidence interval)** |
| **Studies with A(H5N1) virus antigens that were antigenically similar to A(H5N1) viruses circulating in poultry** | | | | | | | | | | | | | | |  |
| Poultry workers | 10 | 91 | 7525 | 0.0 [0.0-5.3] | 0.3 [0.0-0.6] | 10 | 104 | 7525 | 0.0 [0.0-5.3] | 0.5 [0.1-0.8] | 24 | 244 | 13177 | 0.1 [0.0-12.7] | 0.8 [0.5-1.1] |
| Poultry cullers | 3 | 9 | 705 | 0.0 [0.0-3.1] | 0.8 [0.0-2.3] | 3 | 9 | 705 | 0.0 [0.0-3.1] | 0.8 [0.0-2.3] | 3 | 9 | 705 | 0.0 [0.0-3.1] | 0.8 [0.0-2.3] |
| Other occupationally-exposed populations | 2 | 0 | 107 | 0.0 [0.0-0.0] | 0.0 [0.0-1.6] | 2 | 0 | 107 | 0.0 [0.0-0.0] | 0.0 [0.0-1.6] | 2 | 0 | 107 | 0.0 [0.0-0.0] | 0.0 [0.0-1.6] |
| Poultry-exposed residents | 3 | 8 | 2560 | 0.0 [0.0-0.4] | 0.3 [0.1-0.5] | 3 | 8 | 2560 | 0.0 [0.0-0.4] | 0.3 [0.1-0.5] | 5 | 53 | 3611 | 0.0 [0.0-5.6] | 1.0 [0.0-2.0] |
| Exposed healthcare worker | 1 | 0 | 97 | 0.0 [0.0-0.0] | 0.0 [0.0-1.4] | 1 | 0 | 97 | 0.0 [0.0-0.0] | 0.0 [0.0-1.4] | 1 | 0 | 97 | 0.0 [0.0-0.0] | 0.0 [0.0-1.4] |
| Confirmed mixed exposure population | 6 | 40 | 1802 | 1.8 [0.0-7.0] | 1.9 [0.5-3.2] | 7 | 41 | 2221 | 1.0 [0.0-7.0] | 1.5 [0.4-2.5] | 11 | 44 | 3508 | 0.5 [0.0-7.0] | 0.9 [0.2-1.5] |
| Suspected exposure population | 2 | 2 | 333 | 0.3 [0.0-0.6] | 0.6 [0.0-1.5] | 2 | 2 | 333 | 0.3 [0.0-0.6] | 0.6 [0.0-1.5] | 3 | 3 | 976 | 0.2 [0.0-0.6] | 0.2 [0.0-0.5] |
| General population | 5 | 0 | 726 | 0.0 [0.0-0.0] | 0.0 [0.0-0.4] | 5 | 0 | 726 | 0.0 [0.0-0.0] | 0.0 [0.0-0.4] | 12 | 2 | 4780 | 0.0 [0.0-1.0] | 0.0 [0.0-0.1] |
| **Studies without A(H5N1) virus antigens that were antigenically similar to A(H5N1) viruses circulating in poultry** | | | | | | | | | | | | | | |  |
| Poultry workers | 2 | 1 | 341 | 0.4 [0.0-0.9] | 0.1 [0.0-0.7] | 2 | 1 | 341 | 0.4 [0.0-0.9] | 0.0 [0.0-0.1] | 5 | 5 | 2173 | 0.0 [0.0-0.9] | 0.2 [0.0-0.4] |
| Poultry-exposed residents | 1 | 0 | 983 | 0.0 [0.0-0.0] | 0.0 [0.0-0.1] | 1 | 0 | 983 | 0.0 [0.0-0.0] | 0.1 [0.0-0.7] | 2 | 0 | 1201 | 0.0 [0.0-0.0] | 0.0 [0.0-0.1] |
| Household contacts | - | - | - | - | - | - | - | - | - | - | 1 | 0 | 30 | 0.0 [0.0-0.0] | 0.0 [0.0-4.4] |
| Social contacts | - | - | - | - | - | - | - | - | - | - | 1 | 0 | 18 | 0.0 [0.0-0.0] | 0.0 [0.0-7.2] |
| Exposed health care workers | - | - | - | - | - | - | - | - | - | - | 1 | 0 | 177 | 0.0 [0.0-0.0] | 0.0 [0.0-0.8] |
| Suspected exposure population | - | - | - | - | - | - | - | - | - | - | 1 | 0 | 800 | 0.0 [0.0-0.0] | 0.0 [0.0-0.2] |
| General population | - | - | - | - | - | - | - | - | - | - | 2 | 0 | 348 | 0.0 [0.0-0.0] | 0.0 [0.0-0.5] |

Note that the WHO recommended seropositive definition refers to a neutralizing (NT) antibody titer ≥1:80 with a positive result using a 2nd confirmatory assay [i.e. hemagglutination inhibition test (HAI) (HAI antibody titer ≥1:160), enzyme linked immunosorbent assay or western blot assay]. The modified WHO seropositive definition refers to an NT antibody titer ≥1:80 with a positive result using a 2nd confirmatory assay (i.e. HAI antibody titer ≥1:40, ELISA or western blot assay). The non-standardized seropositive definition refers to criteria used to define a seropositive result other than the WHO or modified WHO definitions.

## Table S12. Sensitivity analysis of seroprevalence of antibodies to highly pathogenic avian influenza A(H5N1) virus by type of exposure in studies without ascertainment of influenza-like illness in participants

| **Type of exposure** | **WHO recommended seropositive definition (%)** | | | | **Modified WHO seropositive definition (%)** | | | | **Non-standardized seropositive definition (%)** | | | |
| --- | --- | --- | --- | --- | --- | --- | --- | --- | --- | --- | --- | --- |
|  | **Reference** | **p_min_** | **p_max_** | **p_mean_** | **Reference** | **p_min_** | **p_max_** | **p_mean_** | **Reference** | **p_min_** | **p_max_** | **p_mean_** |
| **Asymptomatic infections** | | | | | | | | | | | | |
| Poultry workers | 0.0 [0.0-0.4] | 0.0 [0.0-0.0] | 0.3 [0.0-0.6] | 0.2 [0.0-0.5] | 0.0 [0.0-0.4] | 0.0 [0.0-0.0] | 0.5 [0.1-0.8] | 0.4 [0.1-0.7] | 0.0 [0.0-0.1] | 0.0 [0.0-0.0] | 0.5 [0.3-0.7] | 0.4 [0.2-0.6] |
| Poultry cullers | 0.4 [0.0-0.9] | - | - | - | 0.4 [0.0-0.9] | - | - | - | 0.4 [0.0-0.9] | 0.0 [0.0-0.3] | 0.0 [0.0-0.3] | 0.0 [0.0-0.3] |
| Other occupationally-exposed populations | 0.0 [0.0-5.3] | - | - | - | 0.0 [0.0-5.3] | - | - | - | 0.0 [0.0-5.3] | - | - | - |
| Poultry-exposed residents | 0.3 [0.1-0.6] | 0.0 [0.0-0.1] | 0.0 [0.0-0.1] | 0.0 [0.0-0.1] | 0.3 [0.1-0.6] | 0.0 [0.0-0.1] | 0.0 [0.0-0.1] | 0.0 [0.0-0.1] | 0.3 [0.1-0.6] | 0.2 [0.0-0.6] | 0.2 [0.0-0.6] | 0.2 [0.0-0.6] |
| Household contacts | - | 0.0 [0.0-12.3] | 0.0 [0.0-12.3] | 0.0 [0.0-12.3] | - | 0.0 [0.0-12.3] | 0.0 [0.0-12.3] | 0.0 [0.0-12.3] | 0.0 [0.0-4.4] | 0.0 [0.0-12.3] | 0.0 [0.0-12.3] | 0.0 [0.0-12.3] |
| Social contacts | - | 0.0 [0.0-3.2] | 0.0 [0.0-3.2] | 0.0 [0.0-3.2] | - | 0.0 [0.0-3.2] | 0.0 [0.0-3.2] | 0.0 [0.0-3.2] | 0.0 [0.0-7.2] | 0.0 [0.0-3.2] | 0.0 [0.0-3.2] | 0.0 [0.0-3.2] |
| Exposed health care workers | 0.0 [0.0-1.4] | 0.0 [0.0-4.7] | 0.0 [0.0-4.7] | 0.0 [0.0-4.7] | 0.0 [0.0-1.4] | 0.0 [0.0-4.7] | 0.0 [0.0-4.7] | 0.0 [0.0-4.7] | 0.0 [0.0-0.7] | 0.0 [0.0-4.7] | 0.0 [0.0-4.7] | 0.0 [0.0-4.7] |
| Other close contacts | 0.0 [0.0-5.5] | - | - | - | 0.0 [0.0-5.5] | - | - | - | 0.0 [0.0-5.5] | - | - | - |
| Confirmed mixed exposure population | 1.9 [0.6-3.1] | 0.0 [0.0-1.6] | 0.0 [0.0-1.6] | 0.0 [0.0-1.6] | 1.4 [0.4-2.4] | 0.0 [0.0-1.6] | 0.0 [0.0-1.6] | 0.0 [0.0-1.6] | 0.9 [0.2-1.6] | 0.0 [0.0-0.2] | 0.0 [0.0-0.2] | 0.0 [0.0-0.2] |
| Suspected mixed exposure population | 0.2 [0.0-0.8] | 0.7 [0.0-2.1] | 0.7 [0.0-2.1] | 0.7 [0.0-2.1] | 0.2 [0.0-0.8] | 0.7 [0.0-2.1] | 0.7 [0.0-2.1] | 0.7 [0.0-2.1] | 0.1 [0.0-0.4] | 0.3 [0.0-0.8] | 0.3 [0.0-0.8] | 0.3 [0.0-0.8] |
| General population | 0.0 [0.0-1.7] | 0.0 [0.0-0.3] | 0.0 [0.0-0.3] | 0.0 [0.0-0.3] | 0.0 [0.0-1.7] | 0.0 [0.0-0.3] | 0.0 [0.0-0.3] | 0.0 [0.0-0.3] | 0.0 [0.0-0.2] | 0.0 [0.0-0.1] | 0.0 [0.0-0.1] | 0.0 [0.0-0.1] |
| **Symptomatic infections** | | | | | | | | | | | | |
| Poultry workers | 0.0 [0.0-0.3] | 0.0 [0.0-0.0] | 0.3 [0.0-0.6] | 0.2 [0.0-0.4] | 0.0 [0.0-0.3] | 0.0 [0.0-0.0] | 0.5 [0.1-0.8] | 0.3 [0.0-0.6] | 0.5 [0.0-1.2] | 0.0 [0.0-0.0] | 0.5 [0.3-0.7] | 0.3 [0.1-0.5] |
| Poultry cullers | 0.1 [0.0-0.5] | - | - | - | 0.1 [0.0-0.5] | - | - | - | 0.1 [0.0-0.5] | 0.0 [0.0-0.3] | 0.0 [0.0-0.3] | 0.0 [0.0-0.3] |
| Other occupationally-exposed populations | 0.0 [0.0-5.3] | - | - | - | 0.0 [0.0-5.3] | - | - | - | 0.0 [0.0-5.3] | - | - | - |
| Poultry-exposed residents | 0.0 [0.0-0.1] | 0.0 [0.0-0.1] | 0.0 [0.0-0.1] | 0.0 [0.0-0.1] | 0.0 [0.0-0.1] | 0.0 [0.0-0.1] | 0.0 [0.0-0.1] | 0.0 [0.0-0.1] | 0.0 [0.0-0.1] | 0.0 [0.0-0.1] | 0.2 [0.0-0.6] | 0.1 [0.0-0.4] |
| Household contacts | - | 0.0 [0.0-12.3] | 0.0 [0.0-12.3] | 0.0 [0.0-12.3] | - | 0.0 [0.0-12.3] | 0.0 [0.0-12.3] | 0.0 [0.0-12.3] | 0.0 [0.0-4.4] | 0.0 [0.0-12.3] | 0.0 [0.0-12.3] | 0.0 [0.0-12.3] |
| Social contacts | - | 0.0 [0.0-3.2] | 0.0 [0.0-3.2] | 0.0 [0.0-3.2] | - | 0.0 [0.0-3.2] | 0.0 [0.0-3.2] | 0.0 [0.0-3.2] | 0.0 [0.0-7.2] | 0.0 [0.0-3.2] | 0.0 [0.0-3.2] | 0.0 [0.0-3.2] |
| Exposed health care workers | 0.0 [0.0-1.4] | 0.0 [0.0-4.7] | 0.0 [0.0-4.7] | 0.0 [0.0-4.7] | 0.0 [0.0-1.4] | 0.0 [0.0-4.7] | 0.0 [0.0-4.7] | 0.0 [0.0-4.7] | 0.0 [0.0-0.7] | 0.0 [0.0-4.7] | 0.0 [0.0-4.7] | 0.0 [0.0-4.7] |
| Other close contacts | 0.0 [0.0-5.5] | - | - | - | 0.0 [0.0-5.5] | - | - | - | 0.0 [0.0-5.5] | - | - | - |
| Confirmed mixed exposure population | 0.1 [0.0-0.2] | 0.0 [0.0-1.6] | 0.0 [0.0-1.6] | 0.0 [0.0-1.6] | 0.0 [0.0-0.2] | 0.0 [0.0-1.6] | 0.0 [0.0-1.6] | 0.0 [0.0-1.6] | 0.1 [0.0-0.2] | 0.0 [0.0-0.2] | 0.0 [0.0-0.2] | 0.0 [0.0-0.2] |
| Suspected mixed exposure population | 0.0 [0.0-0.3] | 0.0 [0.0-0.2] | 0.7 [0.0-2.1] | 0.2 [0.0-0.7] | 0.0 [0.0-0.3] | 0.0 [0.0-0.2] | 0.7 [0.0-2.1] | 0.2 [0.0-0.7] | 0.0 [0.0-0.2] | 0.0 [0.0-0.1] | 0.3 [0.0-0.8] | 0.1 [0.0-0.3] |
| General population | 0.0 [0.0-1.7] | 0.0 [0.0-0.3] | 0.0 [0.0-0.3] | 0.0 [0.0-0.3] | 0.0 [0.0-1.7] | 0.0 [0.0-0.3] | 0.0 [0.0-0.3] | 0.0 [0.0-0.3] | 0.0 [0.0-0.2] | 0.0 [0.0-0.1] | 0.0 [0.0-0.1] | 0.0 [0.0-0.1] |

Note that p_min_, p**_mean_** and p_max_ refer to the minimum, mean and maximum values of the proportion of asymptomatic cases in studies that ascertained influenza-like illness in participant. The WHO recommended seropositive definition refers to a neutralizing (NT) antibody titer ≥1:80 with a positive result using a 2nd confirmatory assay [i.e. hemagglutination inhibition test (HAI) (HAI antibody titer ≥1:160), enzyme linked immunosorbent assay or western blot assay]. The modified WHO seropositive definition refers to an NT antibody titer ≥1:80 with a positive result using a 2nd confirmatory assay (i.e. HAI antibody titer ≥1:40, ELISA or western blot assay). The non-standardized seropositive definition refers to criteria used to define a seropositive result other than the WHO or modified WHO definitions.

## Table S13. Sensitivity analysis of seroprevalence of antibodies to highly pathogenic avian influenza A(H5N1) virus by virus clade in studies without ascertainment of influenza-like illness in participants

| **Virus clade** | **WHO recommended seropositive definition (%)** | | | | **Modified WHO seropositive definition (%)** | | | | **Non-standardized seropositive definition (%)** | | | |
| --- | --- | --- | --- | --- | --- | --- | --- | --- | --- | --- | --- | --- |
|  | **Reference** | **p_min_** | **p_max_** | **p_mean_** | **Reference** | **p_min_** | **p_max_** | **p_mean_** | **Reference** | **p_min_** | **p_max_** | **p_mean_** |
| **Asymptomatic infections** | | | | | | | | | | | | |
| Clade 0 | 0.9 [0.1-1.7] | 0.8 [0.0-2.4] | 2.0 [0.0-6.4] | 1.7 [0.0-5.4] | 0.9 [0.1-1.7] | 0.8 [0.0-2.4] | 2.0 [0.0-6.4] | 1.7 [0.0-5.4] | 0.9 [0.1-1.7] | 0.3 [0.0-0.7] | 1.0 [0.3-1.7] | 0.9 [0.2-1.5] |
| Clade 1 | 0.5 [0.0-1.1] | 0.0 [0.0-0.2] | 0.5 [0.0-1.3] | 0.5 [0.0-1.3] | 0.5 [0.0-1.1] | 0.0 [0.0-0.2] | 0.5 [0.0-1.3] | 0.5 [0.0-1.3] | 0.3 [0.0-0.7] | 0.0 [0.0-0.1] | 0.6 [0.3-1.0] | 0.6 [0.2-1.0] |
| Clade 2.2 | 0.3 [0.0-0.5] | 0.0 [0.0-0.1] | 0.0 [0.0-0.1] | 0.0 [0.0-0.1] | 0.3 [0.0-0.5] | 0.1 [0.0-0.5] | 0.1 [0.0-0.5] | 0.1 [0.0-0.5] | 0.3 [0.0-0.5] | 0.1 [0.0-0.3] | 0.1 [0.0-0.3] | 0.1 [0.0-0.3] |
| Clade 2.3 | 0.0 [0.0-0.4] | 0.0 [0.0-0.1] | 0.0 [0.0-0.1] | 0.0 [0.0-0.1] | 0.1 [0.0-0.4] | 0.0 [0.0-0.1] | 0.0 [0.0-0.1] | 0.0 [0.0-0.1] | 0.0 [0.0-0.1] | 0.0 [0.0-0.0] | 0.1 [0.0-0.2] | 0.1 [0.0-0.2] |
| Clade 2.5 | 0.7 [0.2-1.1] | 0.0 [0.0-0.7] | 0.0 [0.0-0.7] | 0.0 [0.0-0.7] | 0.7 [0.2-1.1] | 0.0 [0.0-0.7] | 0.0 [0.0-0.7] | 0.0 [0.0-0.7] | 0.7 [0.2-1.1] | 0.0 [0.0-0.7] | 0.0 [0.0-0.7] | 0.0 [0.0-0.7] |
| **Symptomatic infections** | | | | | | | | | | | | |
| Clade 0 | 0.3 [0.0-0.9] | 0.0 [0.0-0.1] | 2.0 [0.0-6.4] | 0.9 [0.0-2.7] | 0.3 [0.0-0.9] | 0.0 [0.0-0.1] | 2.0 [0.0-6.4] | 0.9 [0.0-2.7] | 0.3 [0.0-0.9] | 0.0 [0.0-0.1] | 1.0 [0.3-1.7] | 0.4 [0.0-0.7] |
| Clade 1 | 0.0 [0.0-0.2] | 0.0 [0.0-0.2] | 0.5 [0.0-1.3] | 0.4 [0.0-0.9] | 0.0 [0.0-0.2] | 0.0 [0.0-0.2] | 0.5 [0.0-1.3] | 0.4 [0.0-0.9] | 0.0 [0.0-0.1] | 0.0 [0.0-0.1] | 0.6 [0.3-1.0] | 0.4 [0.1-0.7] |
| Clade 2.2 | 0.0 [0.0-0.1] | 0.0 [0.0-0.1] | 0.0 [0.0-0.1] | 0.0 [0.0-0.1] | 0.0 [0.0-0.1] | 0.0 [0.0-0.1] | 0.1 [0.0-0.5] | 0.1 [0.0-0.4] | 0.0 [0.0-0.1] | 0.0 [0.0-0.1] | 0.1 [0.0-0.3] | 0.1 [0.0-0.3] |
| Clade 2.3 | 0.0 [0.0-0.4] | 0.0 [0.0-0.1] | 0.0 [0.0-0.1] | 0.0 [0.0-0.1] | 0.0 [0.0-0.2] | 0.0 [0.0-0.1] | 0.0 [0.0-0.1] | 0.0 [0.0-0.1] | 0.3 [0.0-0.8] | 0.0 [0.0-0.0] | 0.1 [0.0-0.2] | 0.1 [0.0-0.2] |
| Clade 2.5 | 0.0 [0.0-0.1] | 0.0 [0.0-0.7] | 0.0 [0.0-0.7] | 0.0 [0.0-0.7] | 0.0 [0.0-0.1] | 0.0 [0.0-0.7] | 0.0 [0.0-0.7] | 0.0 [0.0-0.7] | 0.0 [0.0-0.1] | 0.0 [0.0-0.7] | 0.0 [0.0-0.7] | 0.0 [0.0-0.7] |

Note that p_min_, p**_mean_** and p_max_ refer to the minimum, mean and maximum values of the proportion of asymptomatic cases in studies that ascertained participant’s influenza-like illness. Note that the WHO recommended seropositive definition refers to a neutralizing (NT) antibody titer ≥1:80 with a positive result using a 2nd confirmatory assay [i.e. hemagglutination inhibition test (HAI) (HAI antibody titer ≥1:160), enzyme linked immunosorbent assay or western blot assay]. The modified WHO seropositive definition refers to an NT antibody titer ≥1:80 with a positive result using a 2nd confirmatory assay (i.e. HAI antibody titer ≥1:40, ELISA or western blot assay). The non-standardized seropositive definition refers to criteria used to define a seropositive result other than the WHO or modified WHO definitions.

## Table S14. Seroconversion rate and seroincidence estimates of human infection with highly pathogenic avian influenza A(H5N1) virus by type of exposure, using non-standardized antibody titer threshold

| **Study population** | **No. of studies** | **No. of positive** | **Person-years** | **No. of participants provided paired serums** | **Observed seroprevalence (median, range)** | **Estimated seroprevalence (95% confidence interval)** | **I^2^ (P)** |
| --- | --- | --- | --- | --- | --- | --- | --- |
| Seroconversion rate (%) | | | | | | | |
| All infections | | | | | | | |
| Poultry workers | 6 | 34 | - | 1856 | 1.4 [0.0-44.0] | 1.3 [0.1-2.5] | **79.4 (<0.001)** |
| Exposed healthcare workers | 2 | 0 | - | 122 | 0.0 [0.0-0.0] | 0.0 [0.0-1.4] | 0.0 (1.000) |
| Other close contacts | 1 | 0 | - | 24 | 0.0 [0.0-0.0] | 0.0 [0.0-5.5] | - |
| Mixed-exposed population | 4 | 2 | - | 497 | 0.0 [0.0-1.0] | 0.1 [0.0-0.7] | 0.0 (0.630) |
| General population | 2 | 0 | - | 1084 | 0.0 [0.0-0.0] | 0.0 [0.0-0.1] | 0.0 (1.000) |
| Asymptomatic infections |  |  |  |  |  |  |  |
| Poultry workers | 1 | 0 | - | 117 | 0.0 [0.0-0.0] | 0.0 [0.0-1.2] | - |
| Exposed healthcare workers | 2 | 0 | - | 122 | 0.0 [0.0-0.0] | 0.0 [0.0-1.4] | 0.0 (1.000) |
| Other close contacts | 1 | 0 | - | 24 | 0.0 [0.0-0.0] | 0.0 [0.0-5.5] | - |
| Confirmed mixed exposure population | 4 | 2 | - | 497 | 0.0 [0.0-1.0] | 0.1 [0.0-0.6] | 0.0 (0.687) |
| Seroincidence rate (cases/100 person-years) | | | | | | | |
| Studies with A(H5N1) epidemics in human or animal | | | | | | | |
| All infections | | | | | | | |
| Poultry workers | 3 | 17 | 148 | 420 | 0.1 [0.0-0.2] | 9.1 [0.0-19.7] | **82.7 (0.003)** |
| Asymptomatic infections | | | | | | | |
| Poultry workers | 1 | 0 | 20 | 117 | 0.0 [0.0-0.0] | 0.0 [0.0-6.5] | - |
| Studies without A(H5N1) outbreaks in humans or poultry | | | | | | | |
| All infections | | | | | | | |
| Poultry worker | 2 | 11 | 1885 | 784 | 0.0 [0.0-0.0] | 0.6 [0.0-1.3] | 0.369 (0.208) |
| General population | 2 | 0 | 2640 | 1084 | 0.0 [0.0-0.0] | 0.0 [0.0-0.1] | 0.0 (1.000) |

Note that the non-standardized seropositive definition refers to criteria to define seropositive results other than the World Health Organization recommended or modified World Health Organization recommended criteria [i.e. a neutralizing (NT) antibody titer ≥1:80 with a positive result confirmed by a 2nd assay (i.e. HAI antibody titer ≥1:40, ELISA or western blot assay)]. Appendix Figures

# Appendix Figures

## **Figure S1. Quality score assigned to sixty-five serological studies by type of exposure to A(H5N1) virus, 1997–2020**

(**A**) The median quality score and the quality score range from 66 serological studies by type of exposure. (**B**) Quality of studies by grade category (i.e. A, B, C and D). Category A spanned studies with a scores ranging from 15 to 18, category B from 10 to 14, category C from 5 to 9, and category D from 0 to 4. The abbreviation “OC” refers to occupationally exposed population and “MP” refers to mixed exposure population. The point in panel A refers to the overall score for each serological study.


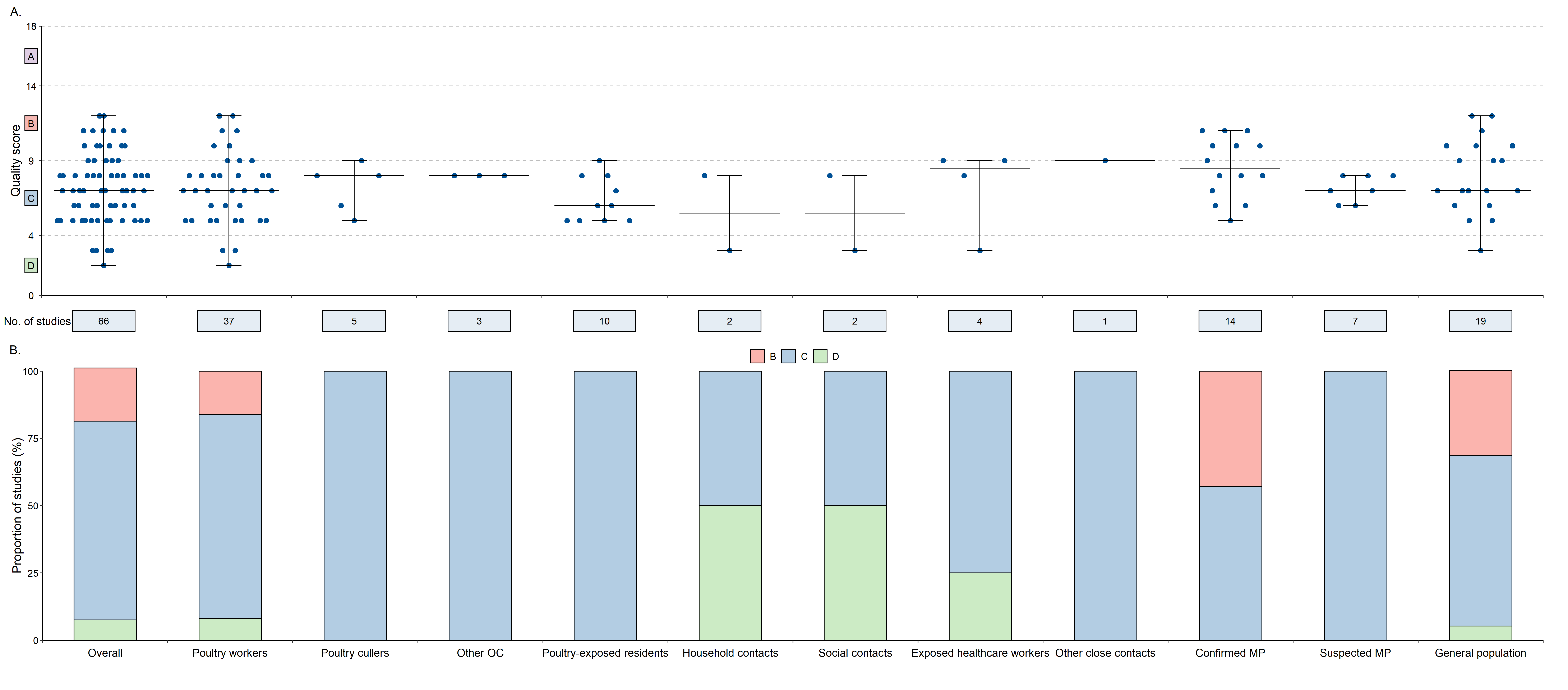


## Figure S2. Pooled estimates of seroprevalence of antibodies to highly pathogenic avian influenza A(H5N1) virus by type of exposure, using modified WHO recommended antibody titer threshold

The modified WHO recommended antibody titer threshold refers to an NT antibody titer ≥1:80 with a positive result using a 2nd confirmatory assay (i.e. HAI antibody titer ≥1:40, ELISA or western blot assay).


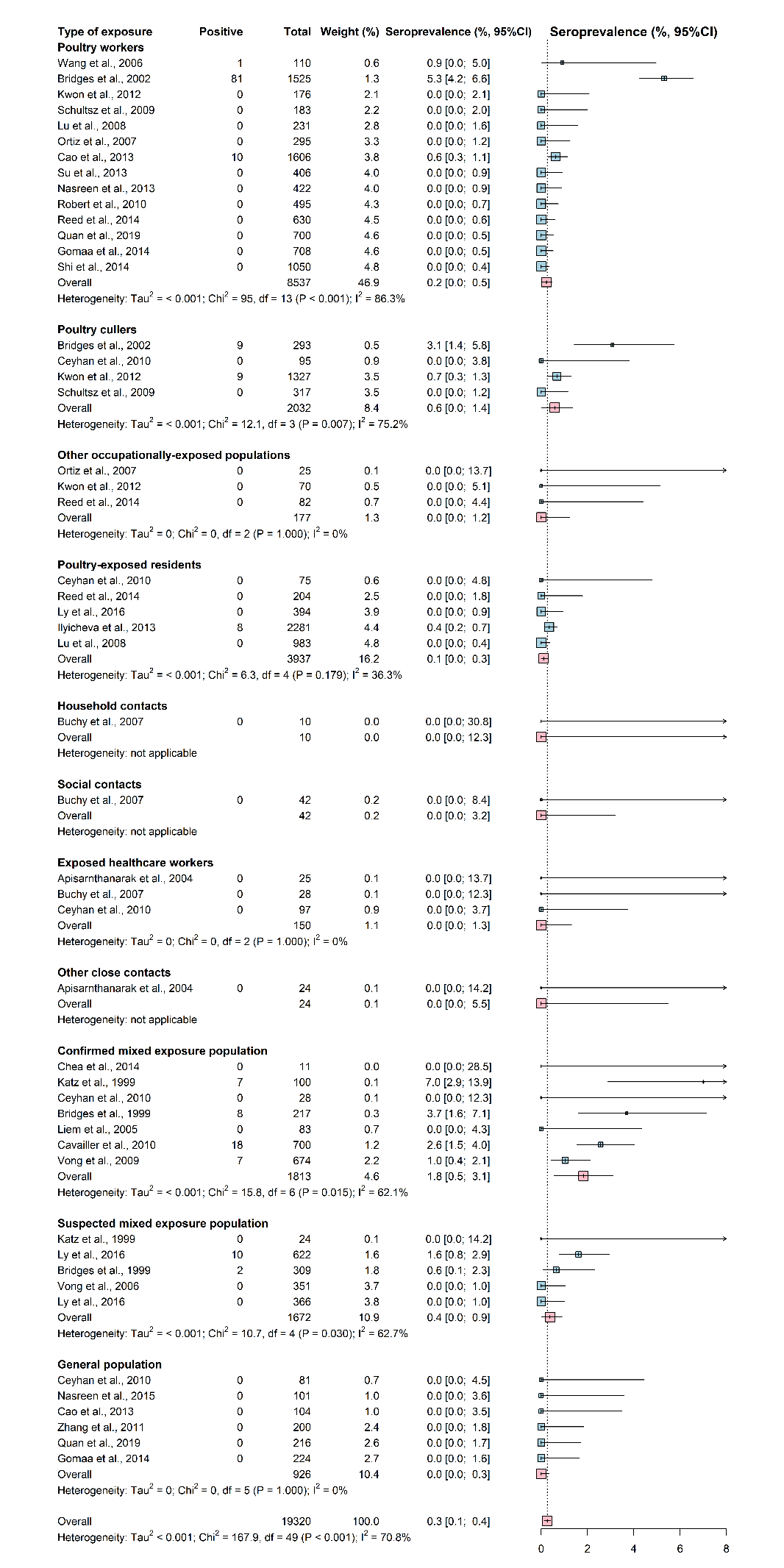


## Figure S3. Pooled estimates of seroprevalence of antibodies to highly pathogenic avian influenza A(H5N1) virus by type of exposure, using non-standardized antibody titer criteria

The non-standardized antibody titer criteria refers to different antibody titer threshold defined by each original study rather than a neutralizing (NT) antibody titer ≥1:80 with a positive result confirmed by a 2nd assay (i.e. HAI antibody titer ≥1:40, ELISA or western blot assay).

**

**

## Figure S4. Relative risk of human infection with highly pathogenic avian influenza A(H5N1) virus by type of exposure, using three antibody titer thresholds to define a seropositive result (World Health Organization recommended, modified World Health Organization recommended and non-standardized)

Panel A, B and C refer to the relative risk of human infection with highly pathogenic avian influenza A(H5N1) virus by type of exposure, using the World Health Organization recommended, modified World Health Organization recommended and non-standardized criteria to define a seropositive result. The WHO recommended seropositive definition in ill persons is a neutralizing (NT) antibody titer ≥1:80 with a positive result using a 2nd confirmatory assay [i.e. hemagglutination inhibition test (HAI) (HAI antibody titer ≥1:160), enzyme linked immunosorbent assay or western blot assay]. The modified WHO seropositive definition refers to an NT antibody titer ≥1:80 with a positive result using a 2nd confirmatory assay (i.e. HAI antibody titer ≥1:40, ELISA or western blot assay). The non-standardized seropositive definition refers to criteria used to define a seropositive result other than the WHO or modified WHO definitions. The abbreviations “PWs”, “PCs”, ”OP”, “PRs”, “”HCs, “SCs”, “HCWs”, “OCs”, “CMP”, “SMP” and “GP” represents poultry workers, poultry cullers, other occupationally-exposed populations, poultry-exposed residents, exposed healthcare workers, household contacts, social contacts, other close contacts, confirmed mixed exposure population, suspected mixed exposure population and general population.


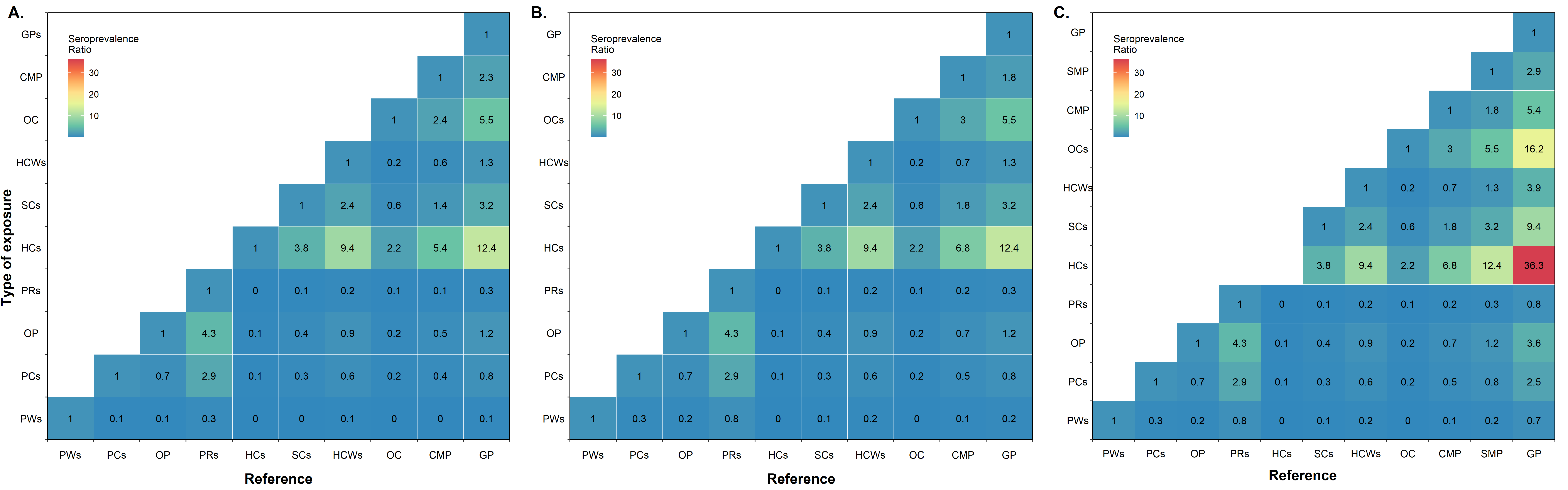


## Figure S5. Estimated seroprevalence of antibodies to highly pathogenic avian influenza A(H5N1) virus in asymptomatic persons by type of exposure and virus clade, using three antibody titer criteria to define a seropositive result (World Health Organization recommended, modified World Health Organization recommended and non-standardized)

Data are presented for the mean seroprevalence of A(H5N1) virus-specific antibodies with 95% confidence interval. The black point represents the mean seroprevalence of A(H5N1) virus-specific antibodies if no antibody was detected. Note that the WHO recommended seropositive definition in ill persons refers to a neutralizing (NT) antibody titer ≥1:80 with a positive result using a 2nd confirmatory assay [i.e. hemagglutination inhibition test (HAI) (HAI antibody titer ≥1:160), enzyme linked immunosorbent assay or western blot assay]. The modified WHO seropositive definition refers to an NT antibody titer ≥1:80 with a positive result using a 2nd confirmatory assay (i.e. HAI antibody titer ≥1:40, ELISA or western blot assay). The non-standardized seropositive definition refers to criteria used to define a seropositive result other than the WHO or modified WHO definitions.


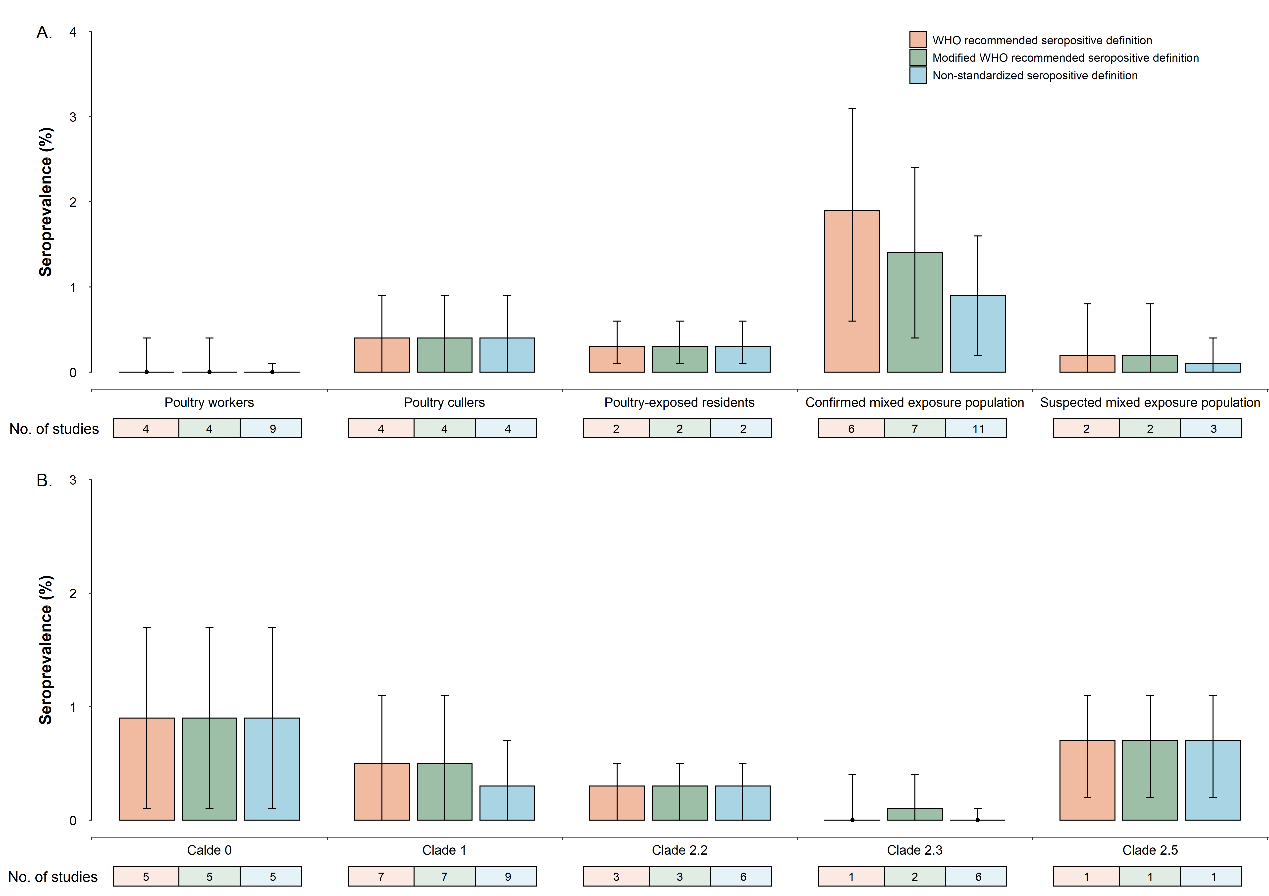


## Figure S6. Estimated seroprevalence of antibodies to highly pathogenic avian influenza A(H5N1) virus in symptomatic persons by type of exposure and virus clade, using three antibody titer criteria to define a seropositive result (World Health Organization recommended, modified World Health Organization recommended and non-standardized)

Data are presented for the mean seroprevalence of A(H5N1) virus-specific antibodies with 95% confidence interval. The black point represents the mean seroprevalence of A(H5N1) virus-specific antibodies if no antibody was detected. Note that the WHO recommended seropositive definition in ill persons refers to a neutralizing (NT) antibody titer ≥1:80 with a positive result using a 2nd confirmatory assay [i.e. hemagglutination inhibition test (HAI) (HAI antibody titer ≥1:160), enzyme linked immunosorbent assay or western blot assay]. The modified WHO seropositive definition refers to an NT antibody titer ≥1:80 with a positive result using a 2nd confirmatory assay (i.e. HAI antibody titer ≥1:40, ELISA or western blot assay). The non-standardized seropositive definition refers to criteria used to define a seropositive result other than the WHO or modified WHO definitions.


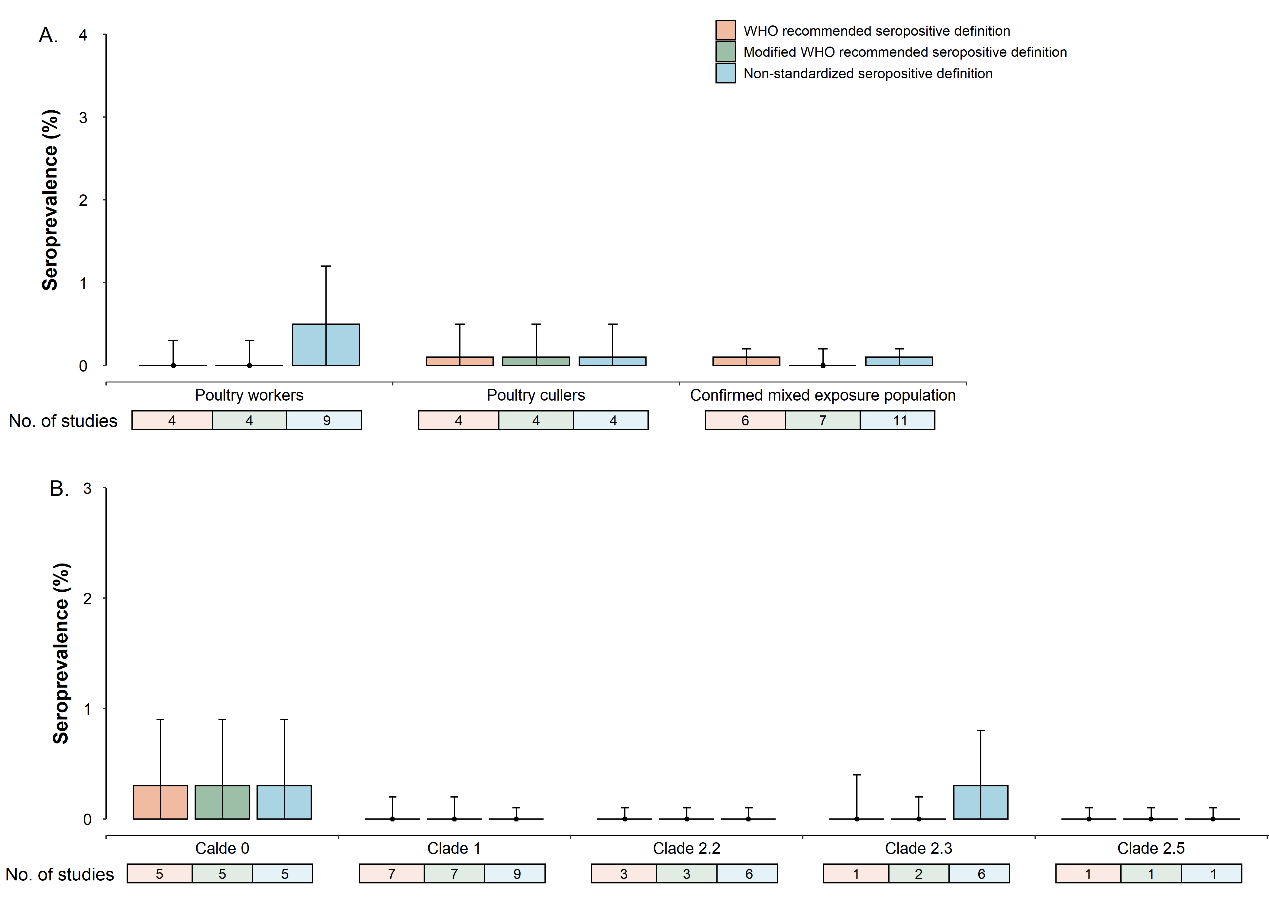


## Figure S7. Subgroup analysis of seroprevalence of antibodies to highly pathogenic avian influenza A(H5N1) virus by type of exposure, using three antibody titer thresholds to define a seropositive result (World Health Organization recommended, modified World Health Organization recommended and non-standardized)

Data are presented for poultry workers (A), poultry cullers (B), poultry-exposed residents **(C)**, and mixed exposure population **(D)** that are presented with high heterogeneity. The abbreviations “AFR”, “EMR”, “EUR”, “AMR”, “SEAR” and “WPR” refer to the African Region, Eastern Mediterranean Region, European Region, Region of the Americas, South-East Asian Region, and Western Pacific Region. Note that the WHO recommended seropositive definition in ill persons refers to a neutralizing (NT) antibody titer ≥1:80 with a positive result using a 2nd confirmatory assay [i.e. hemagglutination inhibition test (HAI) (HAI antibody titer ≥1:160), enzyme linked immunosorbent assay or western blot assay]. The modified WHO seropositive definition refers to an NT antibody titer ≥1:80 with a positive result using a 2nd confirmatory assay (i.e. HAI antibody titer ≥1:40, ELISA or western blot assay). The non-standardized seropositive definition refers to criteria used to define a seropositive result other than the WHO or modified WHO definitions.

**
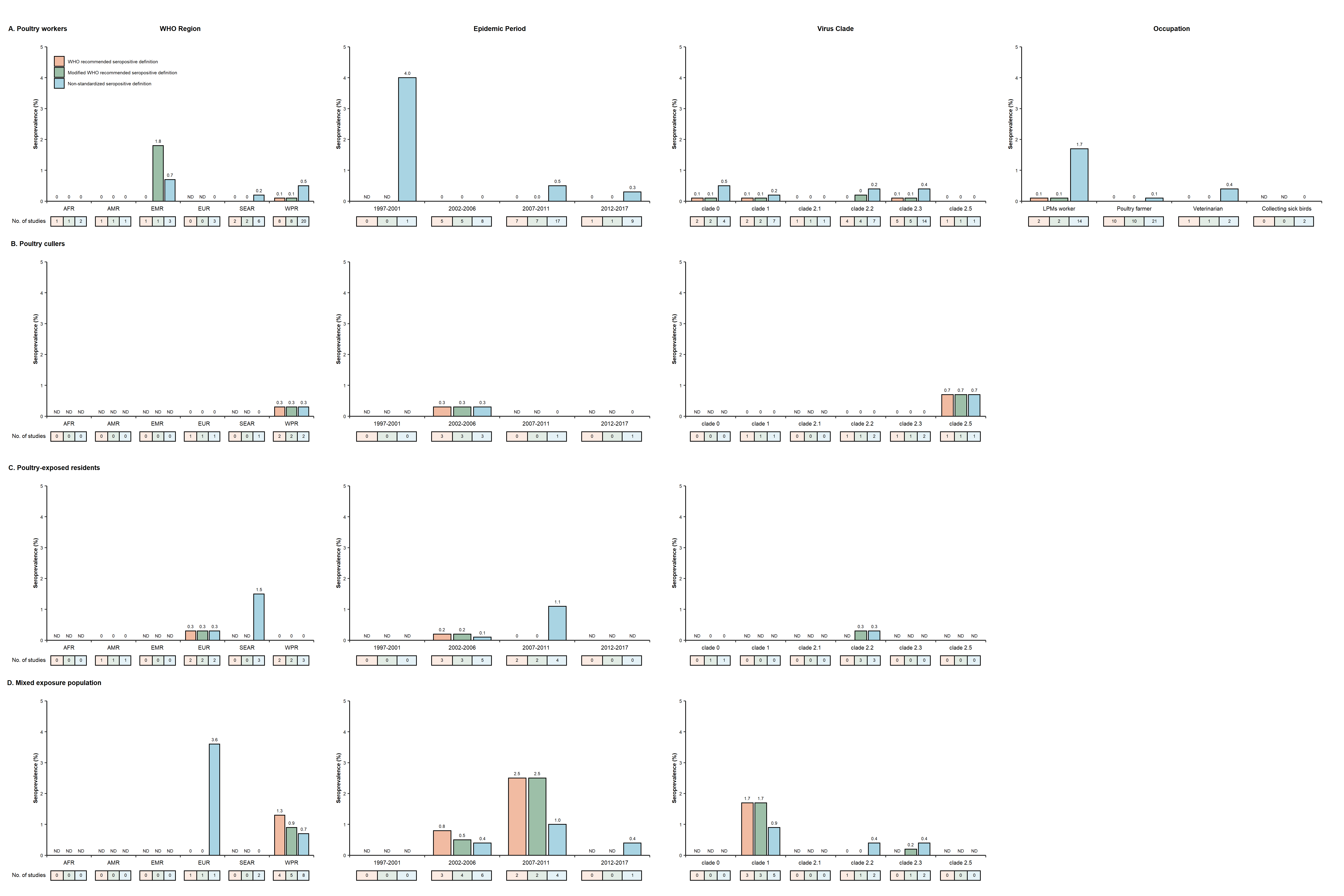
**

## Figure S8. Pooled analysis of seroconversion rates of human infection with highly pathogenic avian influenza A(H5N1) virus by type of exposure, using non-standardized antibody titer criteria.

The non-standardized antibody titer criteria refers to different antibody titer thresholds to define seropositive results other than the World Health Organization recommended or modified World Health Organization recommended criteria [i.e. a neutralizing (NT) antibody titer ≥1:80 with a positive result confirmed by a 2nd assay (i.e. HAI antibody titer ≥1:40, ELISA or western blot assay)].


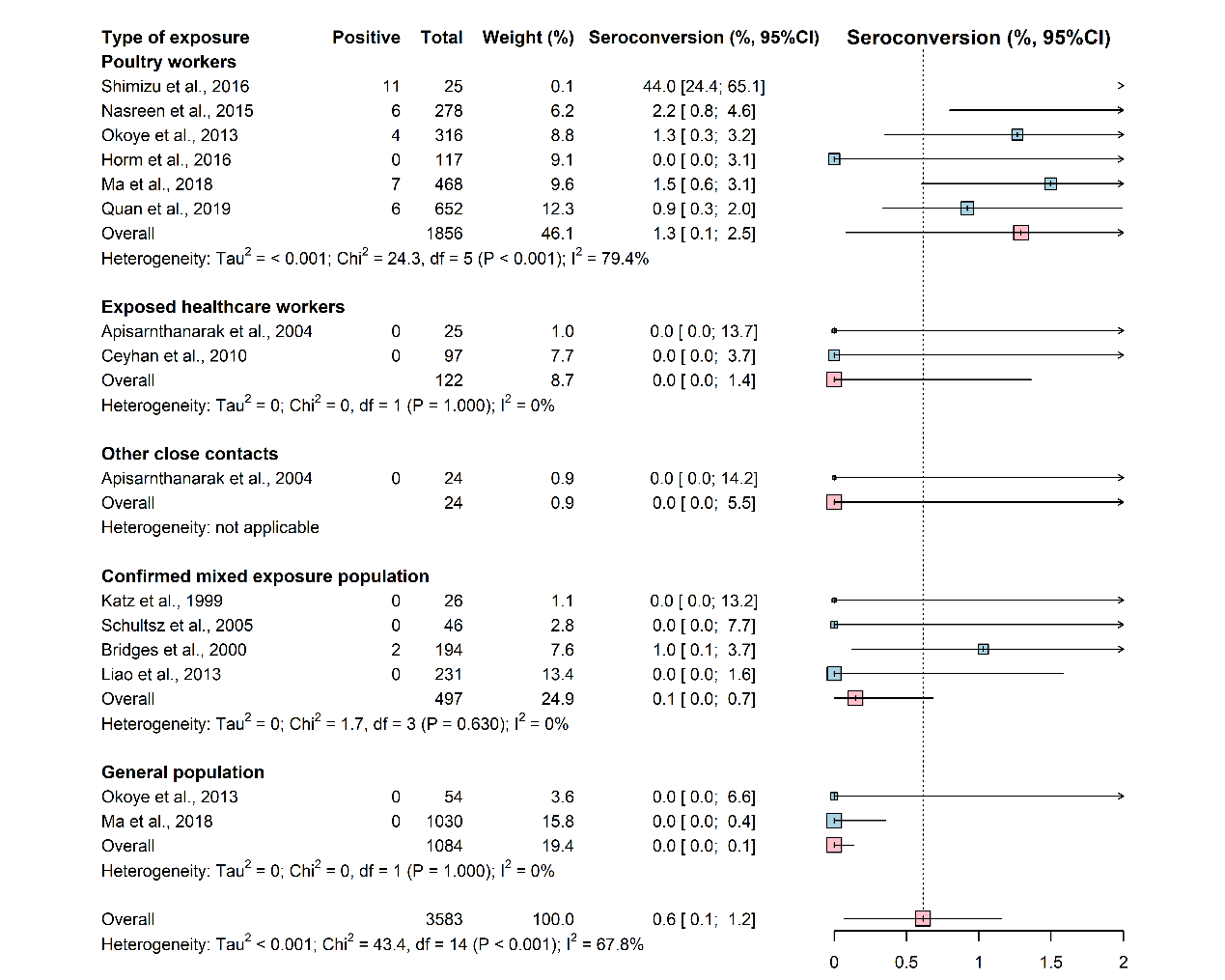


## Figure S9. Pooled analysis of seroincidence of human infection with highly pathogenic avian influenza A(H5N1) virus among studies with A(H5N1) outbreaks, using non-standardized antibody titer criteria

The non-standardized antibody titer criteria refers to different antibody titer thresholds to define seropositive results other than the World Health Organization recommended or modified World Health Organization recommended criteria [i.e. a neutralizing (NT) antibody titer ≥1:80 with a positive result confirmed by a 2nd assay (i.e. HAI antibody titer ≥1:40, ELISA or western blot assay)].


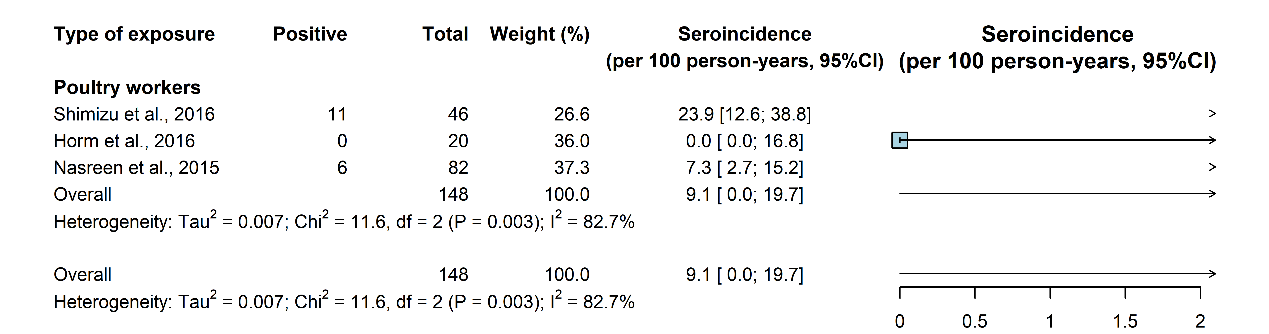


## Figure S10. Pooled analysis of seroincidence of human infection with highly pathogenic avian influenza A(H5N1) virus among studies without A(H5N1) outbreaks, using non-standardized antibody titer criteria

The non-standardized antibody titer criteria refers to different antibody titer thresholds to define seropositive results other than the World Health Organization recommended or modified World Health Organization recommended criteria [i.e. a neutralizing (NT) antibody titer ≥1:80 with a positive result confirmed by a 2nd assay (i.e. HAI antibody titer ≥1:40, ELISA or western blot assay)].


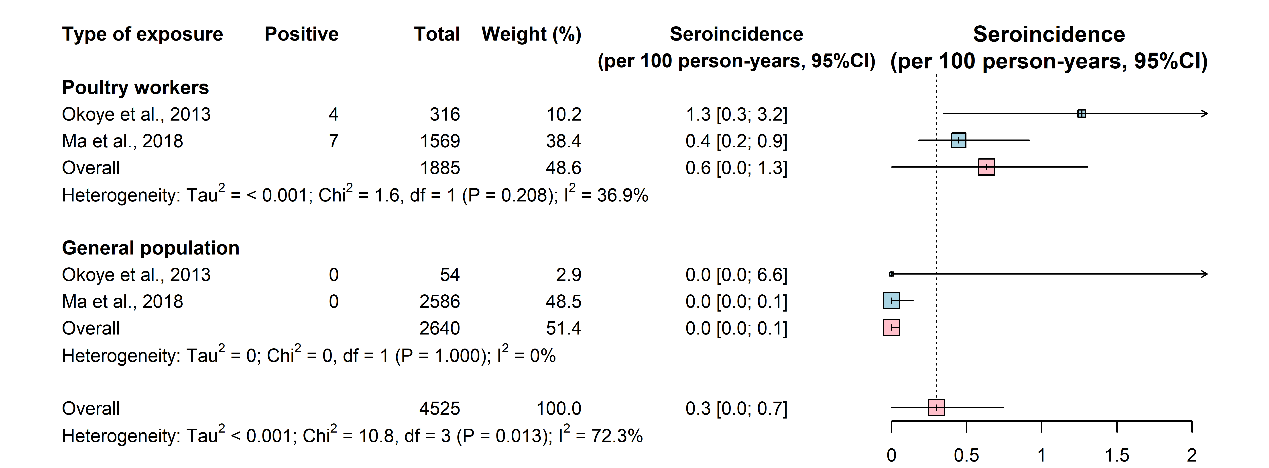


## Figure S11. Estimated seroconversion rate and seroincidence of asymptomatic human infection with highly pathogenic avian influenza A(H5N1) virus by type of exposure, using non-standardized antibody titer criteria.

Data are presented for seroconversion rate for asymptomatic human infection with A(H5N1) virus (**A**), and seroincidence of asymptomatic human infection with A(H5N1) virus considering A(H5N1) outbreaks in human or animal reservoirs occurred (**B**). The non-standardized antibody titer criteria refers to different antibody titer thresholds to define seropositive results other than the World Health Organization recommended or modified World Health Organization recommended criteria [i.e. a neutralizing (NT) antibody titer ≥1:80 with a positive result confirmed by a 2nd assay (i.e. HAI antibody titer ≥1:40, ELISA or western blot assay)].


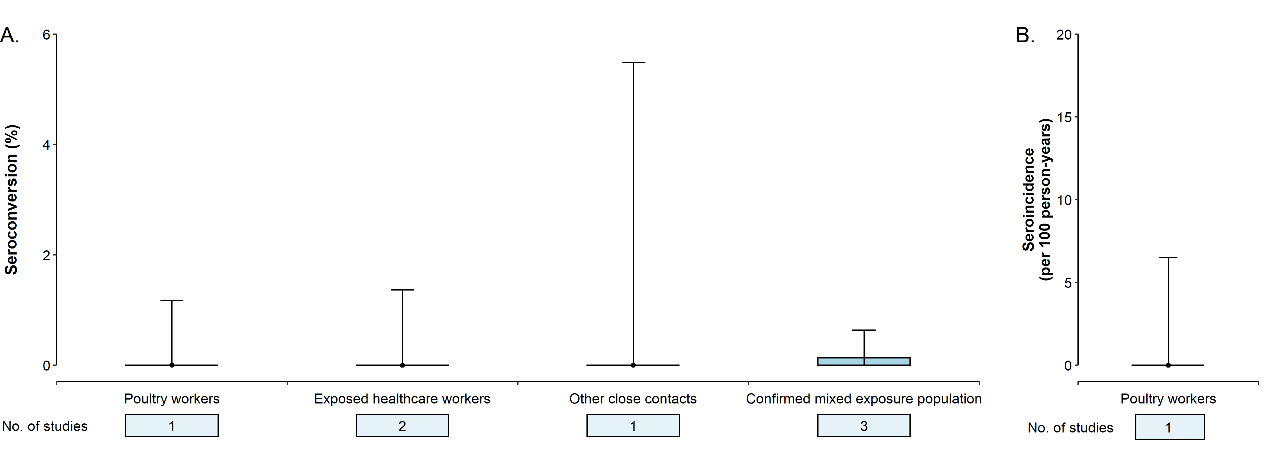


## Figure S12. Funnel plot (with pseudo 95% confidence limits) in studies of the seroprevalence of A(H5N1) virus-specific antibodies using three antibody titer criteria.

Panels A, B and C refer to the assessment of publication bias in studies using A - the World Health Organization recommended, B - modified World Health Organization recommended, and C - non-standardized criteria to define a seropositive result for antibodies to A(H5N1) virus, indicating evidence of virus infection. The WHO recommended seropositive definition in ill persons is a neutralizing (NT) antibody titer ≥1:80 with a positive result using a 2nd confirmatory assay [i.e. hemagglutination inhibition test (HAI) (HAI antibody titer ≥1:160), enzyme linked immunosorbent assay or western blot assay]. The modified WHO seropositive definition refers to an NT antibody titer ≥1:80 with a positive result using a 2nd confirmatory assay (i.e. HAI antibody titer ≥1:40, ELISA or western blot assay). The non-standardized seropositive definition refers to criteria used to define a seropositive result other than the WHO or modified WHO definitions. Note that each circle represents a separate publication for the indicated association.


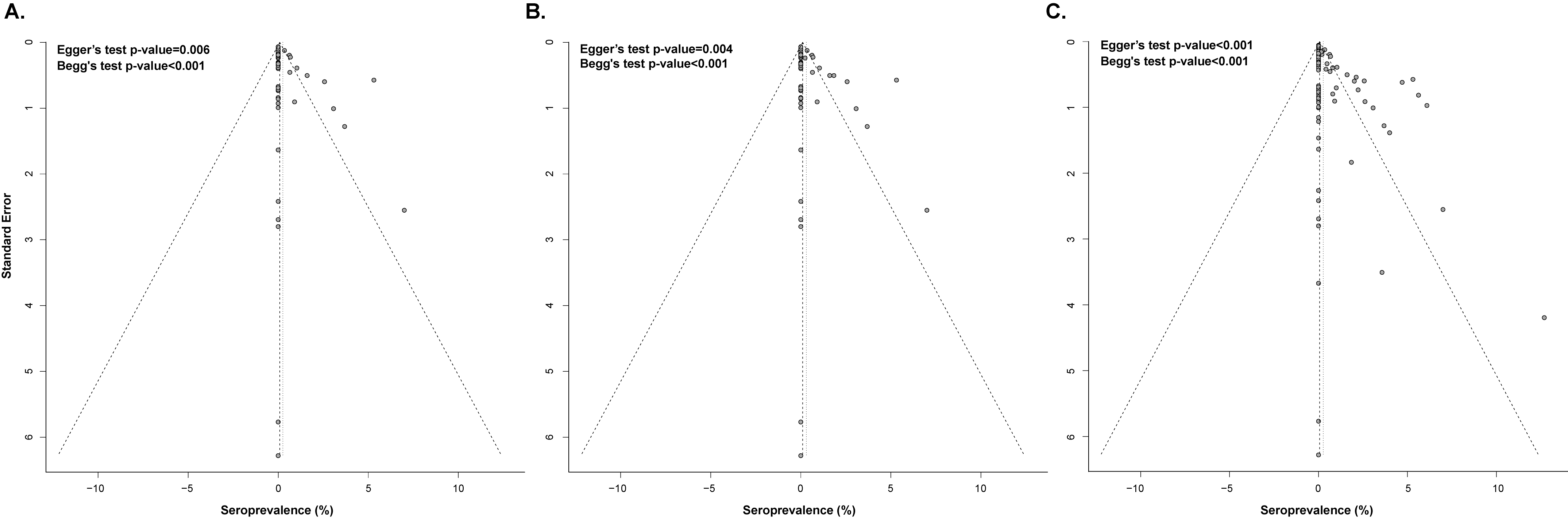


## Figure S13. Estimated seroprevalence of antibodies to highly pathogenic avian influenza A(H5N1) virus in all studies regardless of the availability of methodology, using non-standardized antibody titer criteria.
